# Impact of government policies on the COVID-19 pandemic unraveled by mathematical modelling

**DOI:** 10.1038/s41598-022-21126-2

**Published:** 2022-10-10

**Authors:** Agata Małgorzata Wilk, Krzysztof Łakomiec, Krzysztof Psiuk-Maksymowicz, Krzysztof Fujarewicz

**Affiliations:** 1 Department of Systems Biology and Engineering, Silesian University of Technology, 44-100 Gliwice, Poland; 2 Department of Biostatistics and Bioinformatics, Maria Sklodowska-Curie National Research Institute Gliwice Branch, 44-100 Gliwice, Poland; 3 Biotechnology Center, Silesian University of Technology, 44-100 Gliwice, Poland

**Keywords:** Infectious diseases, Computational science

## Abstract

Since the very beginning of the COVID-19 pandemic, control policies and restrictions have been the hope for containing the rapid spread of the virus. However, the psychological and economic toll they take on society entails the necessity to develop an optimal control strategy. Assessment of the effectiveness of these interventions aided with mathematical modelling remains a non-trivial issue in terms of numerical conditioning due to the high number of parameters to estimate from a highly noisy dataset and significant correlations between policy timings. We propose a solution to the problem of parameter non-estimability utilizing data from a set of European countries. Treating a subset of parameters as common for all countries and the rest as country-specific, we construct a set of individualized models incorporating 13 different pandemic control measures, and estimate their parameters without prior assumptions. We demonstrate high predictive abilities of these models on an independent validation set and rank the policies by their effectiveness in reducing transmission rates. We show that raising awareness through information campaigns, providing income support, closing schools and workplaces, cancelling public events, and maintaining an open testing policy have the highest potential to mitigate the pandemic.

## Introduction

Over two years, more than 320 million confirmed cases and 5.5 million deaths^1^ into the COVID-19 pandemic, health systems are now better equipped with knowledge and means to suppress the SARS-CoV-2 virus transmission, most importantly by mass vaccination. However, optimal control strategy remains a major issue, and was even more challenging before the development of specialized measures. The highly contagious virus spread rapidly throughout the world, gaining the status of a global pandemic less than four months after the first reported case^2^. Governing bodies were faced with the task of containing the pandemic using means generally deemed effective against other contagious diseases.

For detected cases and known exposed individuals, isolation and quarantine were generally implemented. Given the route of infection and high number of asymptomatic cases, considerable efforts have been focused on minimizing non-essential human contact. This resulted in a variety of social distancing policies, including business and school closing, cancellation of public events and restrictions on gatherings, as well as limiting mobility through international and internal travel controls. In extreme cases, emergency states and complete lock-downs were imposed^3^. Due to the enormous socio-economic impact of these interventions and growing controversy surrounding, for example, mandatory facial coverings, it is crucial to determine their effectiveness in mitigating the spread of COVID-19. With the traditional, case–control study design being infeasible for a pandemic happening in real time, the solution must be found through mathematical modelling.

The ability of non-pharmaceutical interventions to reduce coronavirus spread has been the subject of many studies harnessing a wide range of methodologies. The most common approaches include compartmental models^4–11^, agent-based models^12–15^, mobility or social networks^4,7,8,16^, as well as mechanistic models^17^, particle physics^18^ and regression^19,20^. Usually, the research is focused on a specific policy or a small set of policies, such as mask use^12,14,20^, school closing^13,15,19,21^, travel controls^8,14,16^, and social distancing/lockdown^5,7,17,18,20^. The main factor standing in the way of including more restrictions in a single model is parameterization and numerical conditioning; as noted by Castex et al.^10^, timings of control policies are highly correlated. Jorge et al.^11^ solve this issue by constructing a synthetic, time-dependent stringency index. Köhler et al.^9^ balance the complexity of their model against the size of the dataset by taking advantage of prior knowledge and introducing certain constraints on parameter values. These solutions, although effective, are not applicable in cases of poorly known systems where it is impossible to formulate reasonable assumptions.

Here we propose a workflow for prediction of the efficiency of different policies in reducing transmission rates of SARS-CoV-2 infections using a SEIR model incorporating 13 different pandemic control interventions. We demonstrate a multi-step method of parameter estimation without any prior assumptions through individualized modelling of a cohort of European countries, based on adjoint sensitivity analysis, non-linear least squares and coordination. We confirm the satisfying predictive ability of our approach compared to classical modelling strategies over a separate validation time period. Using the developed algorithm we rank control policies by their efficiency in reducing virus transmission rates.

## Methods

### Epidemic model

We simulated the COVID-19 pandemic in the *k*th country using a version of the Susceptible-Exposed-Infectious-Removed (SEIR) model^22^, described by the following system of ordinary differential equations:

1 $$\begin{aligned} {\left\{ \begin{array}{ll} \dot{S_k}(t) = \frac{-\beta _k(t) S_k(t) I_k(t)}{N_k}\\ \\ \dot{E_k}(t) = \frac{\beta _k(t) S_k(t) I_k(t)}{N_k} - k_{EI} E_k(t)\\ \\ \dot{I_k}(t) = k_{EI} E_k(t) - k_{IR} I_k(t) \\ \\ \dot{R_k}(t)= k_{IR} I_k(t) \end{array}\right. }; \quad k=1, ... , K \end{aligned}$$

with initial conditions $S_k(0) = N_k - I_0$, $E_k(0) = 0$, $I_k(0) = I_0$, $R_k(0) = 0$.

**Figure 1:**
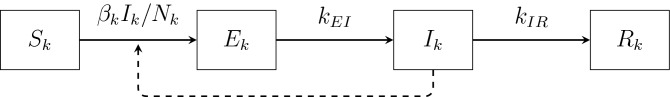
Structure of the SEIR model describing a single country. Susceptible individuals ($S_k$) are exposed ($E_k$) to the virus at a rate $\beta _kI_k/N_k$. They become infectious ($I_k$) at a rate $k_{EI}$ and recover/are removed ($R_k$) at a rate $k_{IR}$.

In the above equations the variables $S_k$, $E_k$, $I_k$, and $R_k$ represent the numbers of individuals who are susceptible, exposed, infectious and removed from compartments for the *k*th country, respectively (Fig. 1). $N_k$ is equal to the sum of all compartments of the SEIR model (1) for the *k*th country

2 $$\begin{aligned} N_k = S_k(t)+E_k(t)+I_k(t)+R_k(t)=\text {const.} \end{aligned}$$

The coefficients $k_{EI}$ and $k_{IR}$ are parameters of the SEIR model (1) which stand for the inverse of times of viral latency (defined as the time to becoming contagious, not to symptom onset) and of recovery from infection, respectively. The function $\beta _k(t)$ represents the time-dependent virus transmission intensity for country *k*.

### Data

The statistics on reported COVID-19 cases were taken from the JHU CSSE data repository^1^. Specifically, we considered two manners of reporting cases in the *k*th country: daily infections on the *i*th day, denoted as $D_k(t_i)$ ($D_{dk}(t_i)$ where it is necessary to indicate observed data, as opposed to $D_{mk}(t_i)$ estimated from modelling) or cumulative cases $C_k(t_i)$ ($C_{dk}(t_i)$ or $C_{mk}(t_i)$ where necessary). Of course, $D_k(t_i)$ and $C_k(t_i)$ satisfy the relation

$$\begin{aligned} C_k(t_i) = \sum _{j = 1}^{i}D_k(t_j) \; . \end{aligned}$$

Although the data contained obvious artifacts resulting from policy changes in reporting cases and retrospective updates, these were not considered exclusion criteria. We considered a time frame from the beginning of the pandemic in Europe to the end of January 2021 when vaccinations (which are not included in the model) started to take effect. To prevent information leakage (evaluating a model using the same data it was trained on), the time period between 1 February 2020 and 31 November 2020 was the basis for parameter estimation, and the final two months (between 1 December 2020 and 31 January 2021) were used to validate the models.

We used publicly available pandemic control information provided by the Oxford COVID-19 Government Response Tracker^3^, focusing on European countries. We selected $r=13$ policies which may potentially influence the spread of SARS-CoV-2, with the exception of restrictions on international travel, not accounted for in the model. We included income support and debt relief, since economic measures, while not directly limiting virus transmission, affect the observance of restrictions.

**Table 1:** 13 government policies to mitigate the pandemic used in the mathematical model. Numbers in brackets represent the original value ranges of each policy.

Containment and closure	Economic	Health system
School closing [0–3]	Income support [0–2]	Public information campaigns [0–2]
Workplace closing [0–3]	Debt/contract relief [0–2]	Testing policy [0–3]
Cancelling public events [0–2]		Contact tracing [0–2]
Restrictions on gatherings [0–4]		Facial coverings [0–4]
Close public transport [0–2]		
Stay at home requirements [0–3]		
Restrictions on internal movement [0–2]		

For increased interpretability, we scaled all values to the range [0–1], with varying degrees of policies denoted by fractions. Countries with incomplete data were excluded from the analysis, resulting in a total of $K = 42$ countries to which the model was applied.

**Figure 2:**
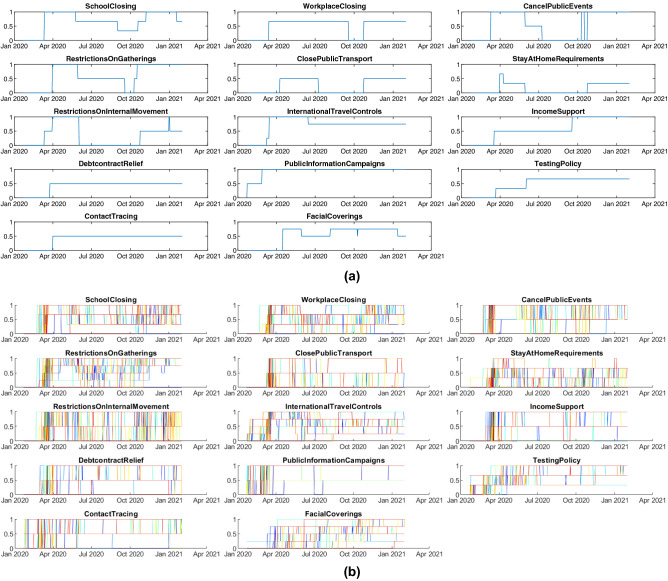
(**a**) Moments of enabling/disabling specific actions to prevent the spread of the COVID-19 pandemic in Poland. The enabling times are the same for debt contract relief and contact tracing, making them indistinguishable for the model. Several policies never reach their maximum level, which makes interpretation difficult. (**b**) Restriction functions for all European countries (each color represents a distinct country). Due to the large number of countries, the figure shown is only illustrative to demonstrate that the problem of indistinguishability of the impact of restrictions, observed for a single country, is now no longer relevant. Separate charts for individual countries can be seen in the Supplementary File.

The extents of particular government policies in a given country can be treated as time-dependent functions, and for policy *i* and country *k* we denote them as $o_{ki}(t)$. Since the policy levels are discrete, $o_{ki}(t)$ takes the form of step functions, examples of which can be seen in Fig. 2a.

### Impact of restrictions

The time-dependent virus transmission intensity $\beta (t)$ varies for each country depending on the implemented restrictions and individual factors including temperature, humidity, population density etc. We describe it as a function of restrictions incorporated during the pandemic with a generic form:

3 $$\begin{aligned} \beta (t) = b (1 - a_1 o_1(t) - a_2 o_{2}(t) - \dots - a_r o_{r}(t)), \end{aligned}$$

where time functions $o_{i}(t)$ for $i=1,2,\dots ,r$ are governments’ policies (Table 1), coefficients $a_i$ are weights reflecting their efficiency, and *b* is a constant value representing the native unaffected virus transmission intensity (possibly influenced by other factors). When all government policies are inactive then, according to the formula (3), the virus transmission rate is unchanged and constant $\beta (t)=b$. In addition, taking into account that functions $o_i(t)$ takes values from 0 to 1 (1 means the strongest level of the particular restriction), it is possible to interpret estimated parameters $a_i$ as effectiveness of the policy $o_i(t)$. For example, if $a_i=0.1$ then the strongest level of the corresponding policy $o_i(t)$ will reduce the virus transmission rate $\beta (t)$ by 10%.

### Modelling approaches for a cohort of entities

The Eq. (3), substituted into the SEIR model (1), means that for each country it is necessary to estimate 14 parameters ($a_1,a_2,\dots ,a_{13}, b$), in addition to the parameters $k_{EI}$ and $k_{IR}$. However, looking at the moments of enabling or disabling selected actions for a single country (in the present case, Poland) presented in Fig. 2a, it is easy to notice that, for example, the actions “Debt / contract relief” and “Contact tracing” were activated practically at the same time. As a result, an appropriately parameterized model will not be able to distinguish the impact of these two actions, and the two parameters responsible for these actions will be a pair of non-estimable^23^ parameters (an increase in one can be compensated by a decrease in the other).

We countered this problem by incorporating data from the entire cohort of countries (Fig. 2b) and developing an individualized approach to modeling.

Consider *K* real objects, systems, or processes. Each of them is described using the same model *M* (mathematical or computational), and the differences in their behavior result from: different initial conditions and different signals affecting the objects (control signals). Each of the models is additionally described by the *m*-element vector $P_k$. Parameters are estimated on the basis of data sets obtained independently for each object: ${\mathscr {U}}_1, {\mathscr {U}}_2, ..., {\mathscr {U}}_K$, which form a common data set ${\mathscr {U}} = \{{\mathscr {U}}_1 \, \cup \, {\mathscr {U}}_2 \, \cup \, ... \, \cup \, {\mathscr {U}}_K\}$.

The task of fitting *K* models to data ${\mathscr {U}}$, can be solved by creating:

(A) a common model,
(B) independent models,
(C) the proposed individualized models.

#### A. Common model

This consists of estimating a common vector of parameters, the same for each of the *K* models, in the form:

4 $$\begin{aligned} P_1 = P_2 = \dots = P_K = [a_1, a_2,..., a_m]^T \end{aligned}$$

based on the common dataset of ${\mathscr {U}}$. The number of estimated unique parameters for the entire set of *K* models is relatively small and is equal to *m*.

This modeling method is typical, for example, in statistics (e.g. linear regression), where one common model is built based on a sample drawn from the population, then applied to each element of the population.

#### B. Independent models

This approach is based on the estimation of the parameter vector in the form

5 $$\begin{aligned} P_k = [b_ {k1}, b_ {k2}, ..., b_ {km}]^T \end{aligned}$$

on the basis of the ${\mathscr {U}}_k$ independently for each of the *K* models, $k = 1,2, ..., K$. The number of estimated parameters in this case is very large and is equal to *Km*.

Such an approach is often used for technical objects and where experimental data is cheap and readily available.

#### C. Individualized models

In this case, we assume that among the *m* parameters characterizing a single model, *r* parameters $a_1, a_2, ..., a_r$ are common parameters and the remaining *q* parameters $b_1, b_2, ..., b_q$ are individual parameters. Of course, $r + q = m$. The entire parameter vector for the *k*th model has the form

6 $$\begin{aligned} P_k = [a_1, a_2, \dots , a_r, b_ {k1}, b_ {k2}, \dots , b_ {kq}] ^ T \end{aligned}$$

The number of estimated parameters in this case is a compromise and equals $r + qK$. Basic differences between approaches A, B and C are presented in Table 2.

**Table 2:**
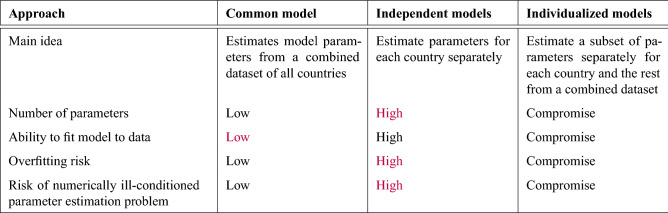
Comparison of approaches to modeling a set of entities. Undesired characteristics are indicated in red.

For individualized simulation of the pandemic, we assumed that parameters $a_{i}$ representing the efficiency of individual policies were common. Biases $b_k$, which also serve as scaling factors for policy efficiencies, were estimated separately for each country. Therefore, based on Eq. (3), the formula for virus transmission intensity in *k*th country can be written as:

7 $$\begin{aligned} \beta _k(t) = b_k (1 - a_{1} o_{k1}(t) - a_2 o_{k2}(t) - \dots - a_r o_{kr}(t))\;. \end{aligned}$$

**Figure 3:**
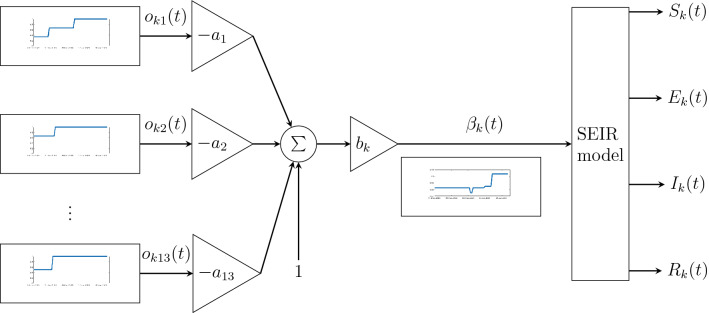
Block diagram of the entire COVID-19 mathematical model for the *k*th country. The non-stationary parameter $\beta _k(t)$ is calculated as a function of government policies $o_{ki}(t)$. The diagram presents *individualized* version of the model (7), where parameters $a_i$ are common for all countries and the parameter $b_k$ is an individual parameter for the *k*th country. The structure of the *common* and the *independent* models are the same—the only difference is related to parameters of the function calculating $\beta _k(t)$.

The full individualized model, containing the static part (7) calculating $\beta _k(t)$ followed by the SEIR model (1), is presented in Fig. 3.

In addition to $k_{EI}$ and $k_{IR}$ which can be viewed as virus properties, the numbers of estimated parameters were: 14 for the common model, 588 for independent models, and 55 for individualized models.

### Parameter estimation

Our goal was to fit the daily infections predicted by the model $D_{mk}(t_i)$ to the observed data $D_{dk}(t_i)$ by minimization of the quadratic objective function:

8 $$\begin{aligned} MSE = \sum _{k = 1}^{K}\sum _{i = 1}^{M} (D_{mk}(t_i) - D_{dk}(t_i))^2. \end{aligned}$$

It is worth explaining at this point how the SEIR model (1) is used to predict daily infections $D_{mk}(t_i)$. The cumulative cases can be expressed as a sum of *I*(*t*) and *R*(*t*) compartments of the model (1):

9 $$\begin{aligned} C_{mk}(t_i) = I_k(t_i) + R_k(t_i) \;. \end{aligned}$$

On the other hand, the daily cases $D_{mk}(t_i)$ may be estimated as a rate of changes of the sum from the above equation, which, taking into account that the time unit is equal to one day, gives

10 $$\begin{aligned} D_{mk}(t_i) \approx \frac{\hbox {d}(I_k(t_i) + R_k(t_i))}{\hbox {d}t} = k_{EI}E_k(t_i)\;. \end{aligned}$$

**Figure 4:**
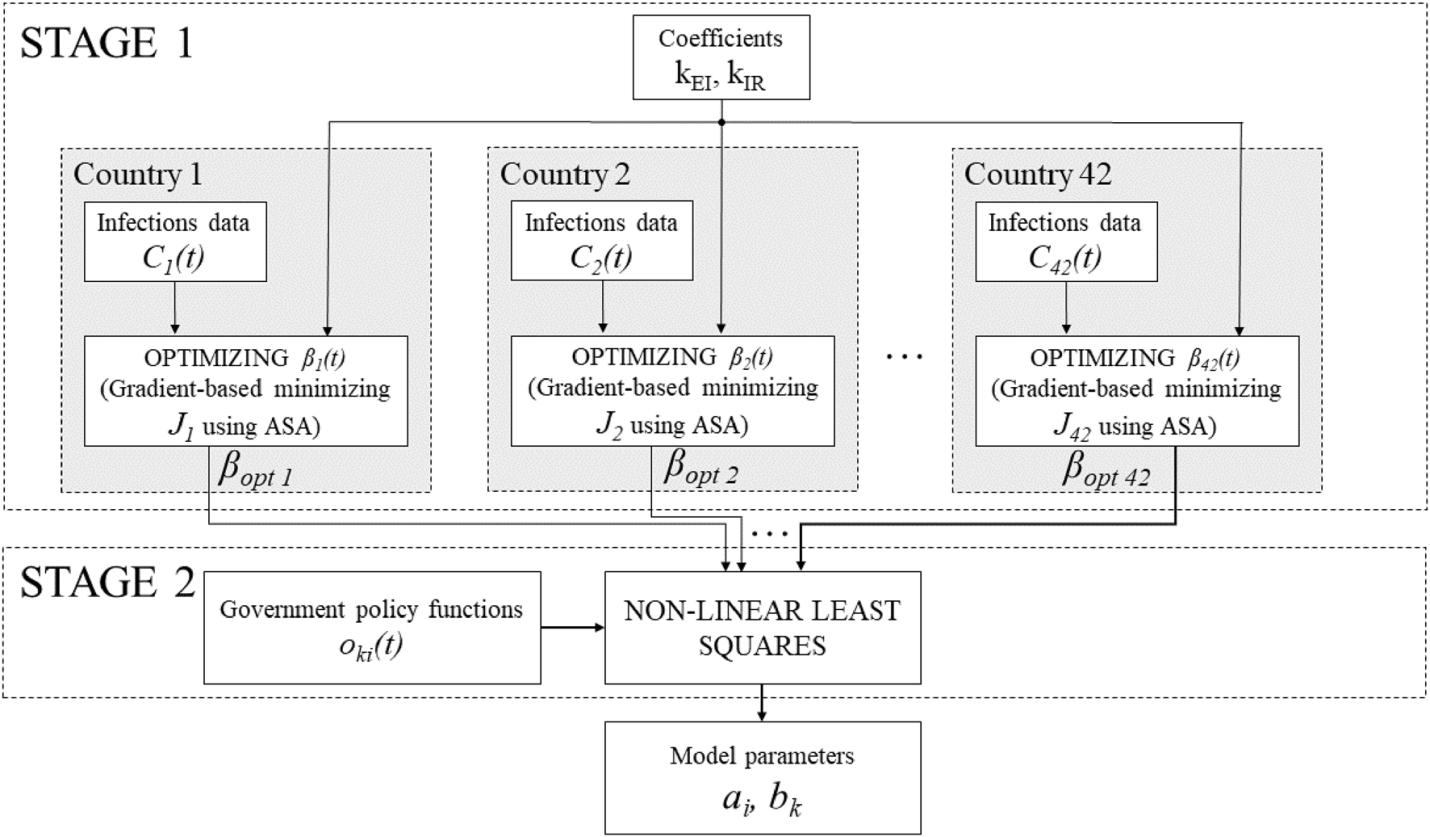
Two-stage parameter estimation workflow for a single set of $k_{EI}$ and $k_{IR}$ values. For each country, optimal $\beta (t)$ is estimated based on cumulative cases using adjoint sensitivity analysis. Based on all the $\beta _{opt}(t)$ functions and restriction functions $o_{ki}(t)$, model parameters are estimated with non-linear least squares.

To reduce computation time we divided the parameter estimation process into two stages (Fig. 4).

#### Two-stage procedure

**Figure 5:**
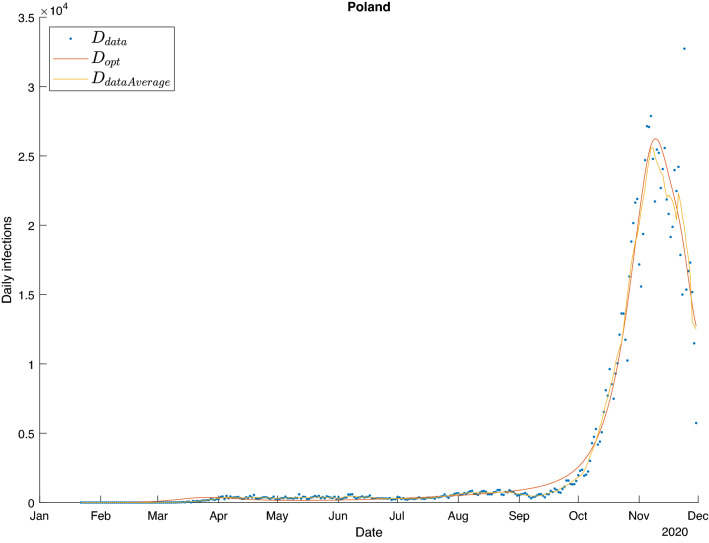
Example fit (Poland) based on $\beta _{opt}$ over the training time period. Blue points represent daily infections, the yellow line the seven-day moving average of daily infections, and the red line is the fit obtained by substituting the $\beta _{opt}(t)$ into the SEIR model. The red and yellow lines practically overlap.

First, in *STAGE 1*, we found the function $\beta _{opt}(t)$ separately for each country by minimizing the following objective function:

11 $$\begin{aligned} J_k = \sum _{i=1}^{M}\Big (C_{mk}(t_i)-C_{dk}(t_i)\Big )^2 \; , \end{aligned}$$

where $C_{mk}(t_i)$ and $C_{dk}(t_i)$ are the numbers of cumulative infections for country *k* at time $t_i$ predicted by the SEIR model and obtained from data, respectively. The reason why we used here the numbers of cumulative cases was the high noise in data of daily cases (Fig. 5).

To minimize the function $J_k$ we used gradient descent method in which the gradient $\nabla _{\beta (t)} J$ was computed using adjoint sensitivity analysis (ASA)^24–30,31^. This stage was independent from government policies (and consequently, from the modelling approach) and produced a near-perfect fit (Fig. 5).

Next, in *STAGE 2*, we employed the non-linear least squares method to obtain the values of parameters *P*, based on the set of functions $\beta _{opt}(t)$ and restriction functions $o_{ki}(t)$.

We repeated this procedure for different values of parameters $k_{EI}$ and $k_{IR}$. The values for which we obtained the best global model performance then served as a starting point for their additional fine-tuning together with optimizing $\beta _{k}(t)$ for all countries. This optimization has been done using two-level direct method of coordination^32^ where $k_{EI}$ and $k_{IR}$ were treated as upper level (coordination) decision variables and $\beta _{k}(t)$ were the bottom level (local) optimized signals. Effectively, while this process can be considered an extension of *STAGE1* in which coefficients $k_{EI}$ and $k_{IR}$ are not entirely arbitrary, it remains consistent with the general notion of a two-stage parameter estimation. The first stage remains independent from government policies.

Analyses were performed using the Matlab environment, version 2021a.

### Model evaluation

For a quantitative comparison of models, the root-mean-square error was calculated for the validation period ($M_v = 62$ days, from 1 December 2020 to 31 January 2021) against daily infections (estimated and observed, denoted as $D_m$ and $D_d$, respectively).

12 $$\begin{aligned} RMSE = \sqrt{\frac{\sum _{i = M+1}^{M+M_v} (D_m(t_i) - D_d(t_i))^2}{M_v}} \; . \end{aligned}$$

Due to varying number of infections in different countries, RMSE values were normalized by the average number of infections in the validation period.

13 $$\begin{aligned} NRMSE = \frac{RMSE}{\frac{1}{M_v} \sum _{i = M+1}^{M+M_v} D_d(t_i)}. \end{aligned}$$

The mean of NRMSE over all countries was considered a general measure of model quality.

## Results

### Adjusting $k_{EI}$ and $k_{IR}$ parameters

The predictive ability of the model depends heavily on the values of parameters $k_{EI}$ and $k_{IR}$ (Table 3) with overall best performance achieved by the individualized model for values $k_{EI} = 0.25$ and $k_{IR} = 0.1$. It is worth noting that for common and individualized approaches, the lowest errors coincide with $k_{EI}$ and $k_{IR}$ corresponding to latency and recovery times within ranges estimated for SARS-CoV-2^33^. This is, however, not the case for independent models.

**Table 3:** Average NRMSE for different values of parameters $k_{EI}$ and $k_{IR}$ parameters. The lowest error for each modelling approach is shown in bold. The lowest error overall was achieved for the individualized approach for $k_{EI} = 0.25$ and $k_{IR} = 0.1$.

$k_{EI}$	$k_{IR}$	Common model	Independent models	Individualized models
0.05	0.1	2.579	**3.712**	2.762
0.15	0.1	2.376	4.599	1.876
0.20	0.1	2.400	4.232	1.722
0.25	0.1	**2.356**	5.091	**1.675**
0.15	0.01	4.983	5.669	5.225

The final estimates, obtained with the coordination method, are $k_{EI} = 0.2605$ and $k_{IR} = 0.1020$, which corresponds to approximately 3.8 days of latency period and 9.8 days of infectious period. These parameter values were used in the subsequent analyses.

### Predictive ability of the model

**Figure 6:**
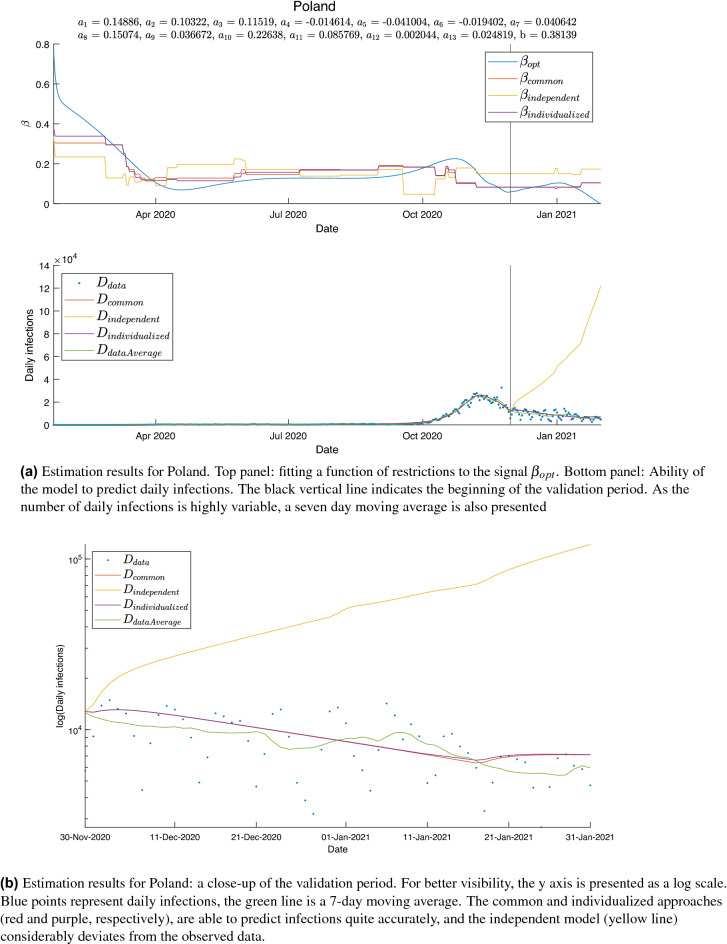
Prediction results.

The estimation results for Poland, presented in Fig. 6, are a representative example of a general tendency observed in the behavior of our models. With few exceptions, while predictions obtained with common and individualized approaches are reasonably close to the observed daily infection numbers, the independent model displays typical signs of overfitting. The results for all countries are presented in the Supplementary Material.

Performances of all the models are outlined in Table 4. Common and individualized approaches are similar in terms of the number of countries where they are the best fit. Nevertheless, errors of the individualized approach where it proved less efficient tend to be smaller, which is reflected in the average NRMSE of 1.682, as compared to 2.374 for the common model, as well as the standard deviations. The independent modelling approach appears inferior in all respects.

**Table 4:** Performances of the final models (with optimized values of coefficients $k_{EI} = 0.2605$ and $k_{IR} = 0.1020$). For each country, lowest error values are in bold. Average NRMSE and standard deviation for each approach is also presented.

Country	Common	Independent	Individualized	Country	Common	Independent	Individualized
**NRMSE by country**							
Albania	4.09	**0.62**	0.93	Latvia	**0.70**	8.13	0.88
Andorra	**0.71**	8.94	0.90	Lithuania	0.70	15.90	**0.42**
Austria	**0.50**	0.80	0.55	Luxembourg	2.96	9.65	**1.75**
Belarus	24.39	2.96	**0.81**	Malta	**0.39**	10.39	0.69
Belgium	1.75	**0.76**	5.06	Moldova	4.97	19.14	**1.27**
Bosnia and Herzegovina	17.32	** 2.34**	11.86	Monaco	**0.93**	1.06	1.03
Bulgaria	3.78	3.93	**1.62**	Netherlands	0.51	4.70	**0.43**
Croatia	**3.01**	22.97	3.67	Norway	1.45	1.97	**0.34**
Cyprus	**0.89**	0.97	1.01	Poland	**0.36**	6.59	**0.36**
Czech Rep.	**0.54**	0.58	0.66	Portugal	0.87	**0.55**	0.59
Denmark	0.70	0.87	**0.66**	Romania	**0.33**	0.53	0.40
Estonia	1.27	15.52	**0.46**	Russia	0.35	**0.24**	1.14
Finland	**0.37**	1.29	0.39	San Marino	**0.83**	0.87	0.93
France	**0.68**	0.97	1.40	Serbia	1.33	2.11	**0.47**
Germany	1.09	**0.66**	3.81	Slovenia	**0.67**	13.27	0.90
Greece	0.68	0.94	**0.37**	Spain	1.14	**1.10**	**1.10**
Hungary	3.15	**1.31**	2.56	Sweden	1.45	4.34	**1.44**
Iceland	1.27	15.73	**1.11**	Switzerland	1.15	**0.88**	1.05
Ireland	1.32	**1.27**	1.34	Turkey	**3.47**	3.84	3.54
Italy	**2.14**	4.28	8.42	Ukraine	**1.32**	2.09	2.67
Kosovo	3.53	10.58	**1.17**	UK	0.70	0.96	**0.48**

		Common model		Independent models		Individualized models	
**Summary**							
Average NRMSE		2.374		4.920		1.682	
Standard deviation		4.414		5.906		2.222	

### Impact of control policies

**Figure 7:**
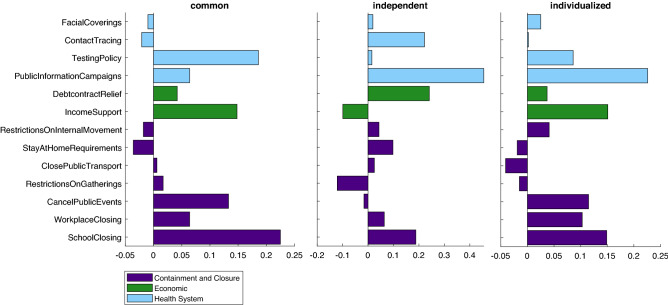
Estimated weights for control policies using different approaches. High positive values correspond to effective policies. Since in independent models each country has its own parameter set, for demonstrative purposes values for Poland are presented.

The weights $a_i$ representing the effectiveness of different interventions varied between approaches, although the general inference is similar for common and individualized approaches (Fig. 7). For the common model they were in the range between $-0.04$ and 0.22, for the independent models between $-2.41$ and 1.66, and for the individualized models between $-0.04$ and 0.23.

For the individualized models, Table 5 shows the ranking of policies from the most to the least effective.

**Table 5:** Ranking of policies according to effectiveness.

Rank	Policy	${a_i}$
1	Public information campaigns	0.226
2	Income support	0.151
3	School closing	0.149
4	Cancel public events	0.115
5	Workplace closing	0.103
6	Testing policy	0.086
7	Restrictions on internal movement	0.041
8	Debt/contract relief	0.037
9	Facial coverings	0.025
10	Contact tracing	0.002
11	Restrictions on gatherings	− 0.015
12	Stay at home requirements	− 0.019
13	Close public transport	− 0.041
	Sum of all $a_i$	0.859

## Discussion

Correct model parametrization and numerical conditioning is a non-trivial task, particularly in complex systems with sparse data available for estimation. This issue became a major obstacle in determining the optimal strategy for non-pharmaceutical interventions dedicated to mitigating the spread of the COVID-19 pandemic, as the coincidence of several policies rendered the impact of individual ones indistinguishable in terms of mathematical modelling. Furthermore, highly noisy data may cause difficulties to obtain a converged solution.

The perhaps most intuitive approach, which is modelling each country independently, may prove inadequate for estimation of a large number of parameters, particularly when no constraints on their values are imposed. Poor numerical conditioning may result in unrealistic estimations. This became apparent for the parameters $k_{EI}$ and $k_{IR}$, for which good estimates can be found in literature. The mean latent period for COVID-19 has been estimated as 3.3 days^34^, and the mean incubation period (which is reported to be up to 2 days longer than the latent period) as 5.5 days^35^, 5.8 days^33^ or 5.6 days^36^. The latent period (calculated as $\frac{1}{k_{EI}}$) for which the lowest errors were obtained, was approximately 4 days for the common and individualized models, and 20 days for the independent models. While the common and individualized approach produce the best results for latency times close to the actual values reported for COVID-19, the independent approach worked best for an unrealistic value. Moreover, NRMSE values for the independent approach vary considerably even for small changes of $k_{EI}$ within a realistic range (0.15, 0.2 and 0.25), reinforcing the expectation of its numerical instability.

We developed a workflow for assessing the effect of pandemic control policies in Europe utilizing data from the entire cohort of countries. Our individualized approach yields satisfactory results with no assumptions regarding parameter values. Meanwhile, for independent models poor numerical conditioning was evident in that the estimated weights $a_i$ often reached negative values (even as low as $-2.41$), which in practice would suggest that the interventions increased virus transmission rates. Indeed, while introduction of a non-negativity constraint significantly improved the performance of independent models, it had little effect on individualized models. A common model has similar numerical advantages, however it does not capture individual country characteristics.

Comparing the values $a_i$ for individual policies, there is a pronounced inconsistency between modelling approaches, particularly for the independent models. For example, “School closing” had a weight of 0.225 for the common model, 0.149 for the individualized model, and between $-0.443$ and 0.718 for the independent models. As discussed above, the data used for fitting the independent models is too scarce and noisy for the considered number of parameters to be estimable. Hence, the estimations obtained with this approach cannot reliably be used for inference. The differences between weights estimated by common and individualized models, although present, do not lead to drastically different conclusions—indeed, both models indicate that school closing has a large impact on virus transmission.

Application of the methodology proposed here enabled a ranking of government policies according to their impact. The most effective measures are public information campaigns followed closely by income support, school and workplace closures, cancellation of public events and open testing policy. The high rank of information campaigns emphasises the importance of knowledge and public access to verified information in mitigating a crisis. As hypothesised at the stage of pre-selecting policies to be included in the model, providing financial support to those affected by the pandemic proved effective, likely through reducing the necessity of bypassing restrictions to secure livelihood. An open testing policy, particularly not limiting testing to symptomatic individuals, ensures higher detection rates and consequently more effective case isolation.

Perhaps surprisingly, some of the intuitively powerful measures such as facial coverings, restrictions on gatherings and stay at home requirements ranked relatively low. One possible explanation is that the restrictions pertain to formal policies, not to the degree of their observance. Indeed, a certain pattern may be noticed. Assuming a threshold of 0.05, the ineffective policies comprise restrictions on internal movement, debt/contract relief, facial coverings, contact tracing, restrictions on gatherings, stay at home requirements and closing public transport. Almost every policy deemed ineffective is less tangible, difficult to enforce and monitor. Restrictions on internal movement, gatherings and stay at home requirements would require frequent and strict controls beyond the capabilities of any country’s police force. Precise and exact contact tracing is practically impossible to achieve since it relies on either the entire population providing a perfect and constant account of all their encounters or using a geolocation device at any given time. Facial coverings, as possibly the most debated restriction, have met with considerable resistance. Moreover, especially at the beginning of the pandemic, limited sanitary resources were rerouted to the health system with the rest of the general population using homemade coverings. In contrast, the policies deemed effective are straightforward and well-defined. Public information campaigns, income support, school closing, cancellation of public events, workplace closing and testing policy are all top-down, independent of the individual and easily traceable. The low position in the ranking of using facial coverings may also be attributed to infections occurring primarily by prolonged contact, for example with family, during events, or at schools or workplaces (which ranked high), where masks are often neglected.

Notably, the sum of all $a_i$ values is 0.859, which suggests implementing all restrictions simultaneously at the highest level would decrease the virus transmission rate to approximately 14% of its original value.

This study is not without limitations. The SEIR model used to model the pandemic is relatively simple as it does not consider repeated infections or population structure. Furthermore, our estimations were based on confirmed cases constituting only a fraction of the actual number of infections, many of which were asymptomatic. One solution could be correcting the number of cases for testing capacity. However, there are considerable inconsistencies and gaps in reporting numbers of tests: in many countries the number of tests was reported only several months into the pandemic, in others only cumulative number was reported weekly. In total, to incorporate testing data, even allowing for a certain percentage of missing values, 23 countries would have to be excluded from the analysis. The number of deaths could also be used as an alternative indicator of the course of the pandemic. Yet, the reporting of COVID-related deaths also varied by country, even evolving within a single country—the reported deaths were sometimes interpreted as ones directly caused by SARS-CoV-2, otherwise as any death coinciding with infection. To some extent, testing accessibility is incorporated into our model as one of the policies (“Testing policy”). Lower levels, usually observed at the beginning of the pandemic, indicate limited access to tests. Higher levels, denoting unlimited access to testing, are typically observed at the later time when the testing system was fully developed. The importance of testing capacity, allowing for more effective isolation of infected individuals, is reflected in the high coefficient for this policy.

This work may be extended in several directions. Additional compartments may be included in the model, representing for example vaccinated individuals, asymptomatic cases, quarantined individuals, movement of people between countries and so on. Alternative forms of the restriction impact function may also be considered, including a multiplicative form.

Nevertheless, our findings lay the foundation for a new approach to parameter estimation and provide a tool for planning pandemic control strategy.

## Supplementary Information

Supplementary Information.

## Data Availability

The data used in this study was taken from publicly available databases: Oxford COVID-19 Government Response Tracker (OxCGRT) https://github.com/OxCGRT/covid-policy-tracker. JHU CSSE COVID-19 data repository https://github.com/CSSEGISandData/COVID-19. Definite data and MATLAB code were made available in a GitHub repository https://github.com/AgataWilk/COVID19_ImpactOfPolicies.git.
