# Supplementary material for: Impact of government policies on the COVID-19 pandemic unraveled by mathematical modelling
Source: Sci Rep. 2022 Oct 10;12:16987. doi: 10.1038/s41598-022-21126-2 (PMC9549859; doi:10.1038/s41598-022-21126-2)
Supplement: Supplementary file 1 — Supplementary Information. [file 41598_2022_21126_MOESM1_ESM.pdf]

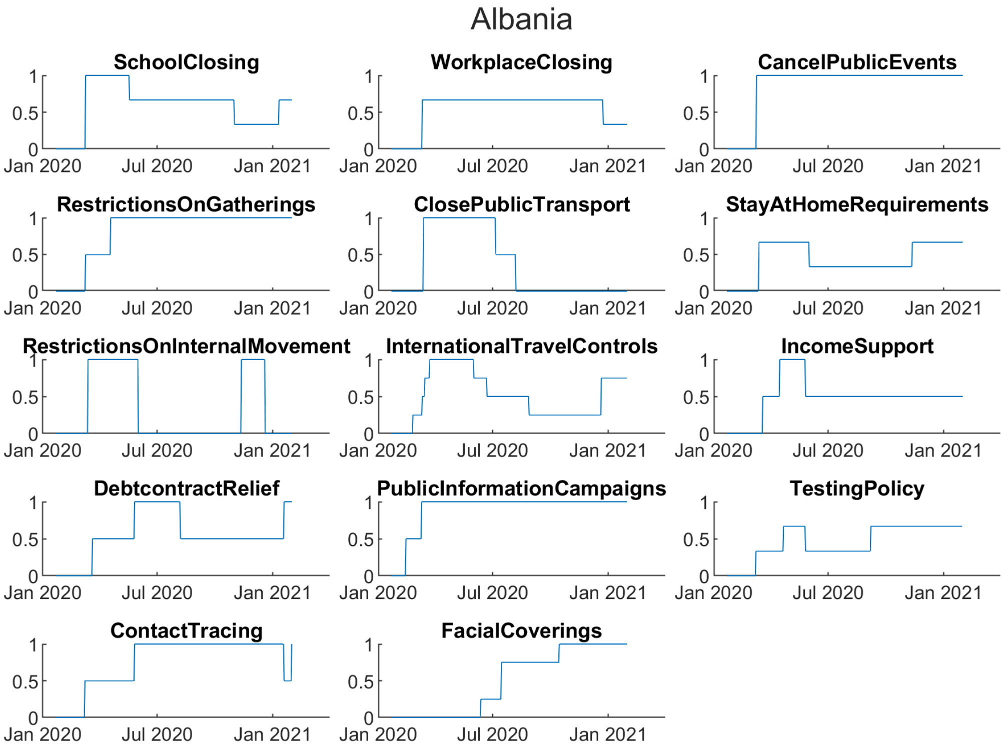

Supplementary Figure 1. The timeline of policies introduced to mitigate the spread of the pandemic — Albania.

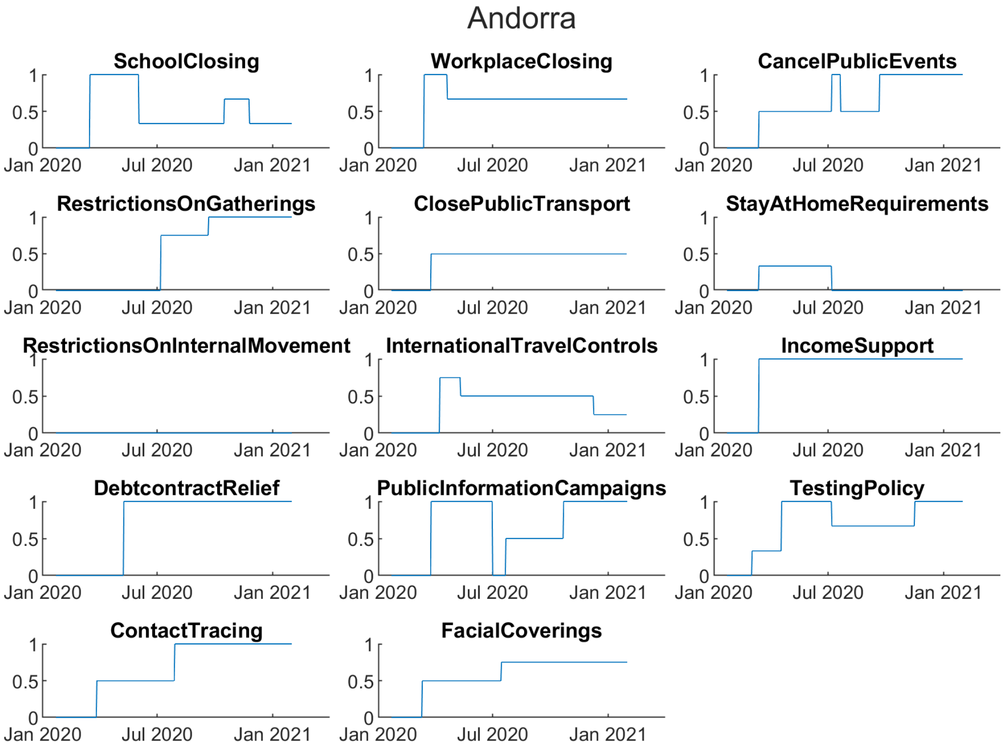

Supplementary Figure 2. The timeline of policies introduced to mitigate the spread of the pandemic – Andorra.

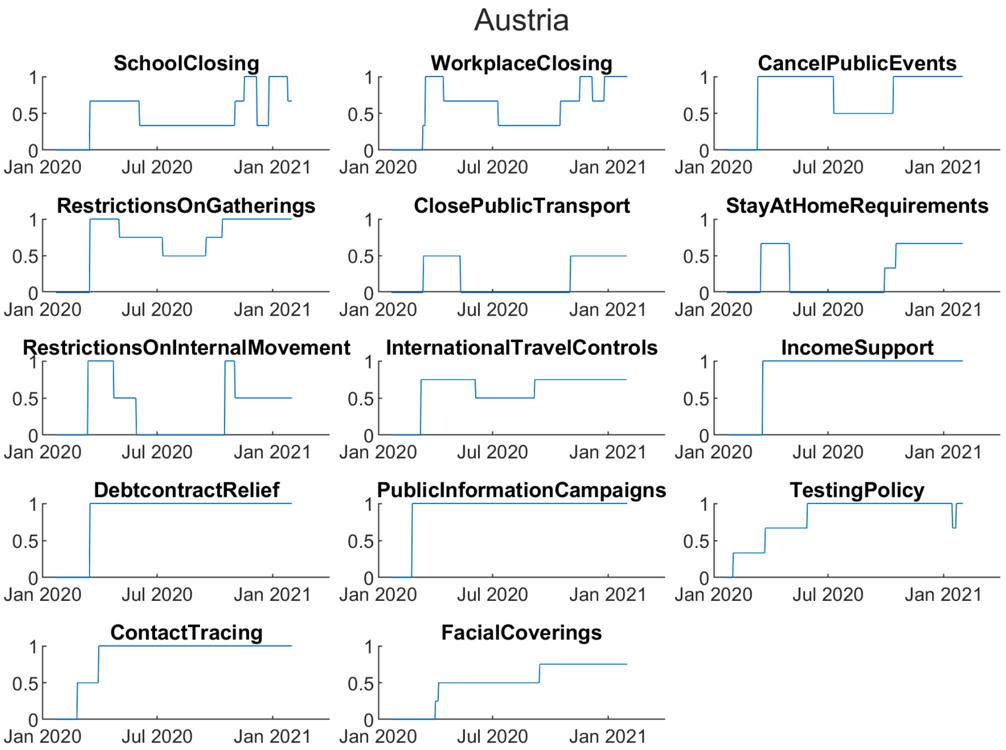

Supplementary Figure 3. The timeline of policies introduced to mitigate the spread of the pandemic — Austria.

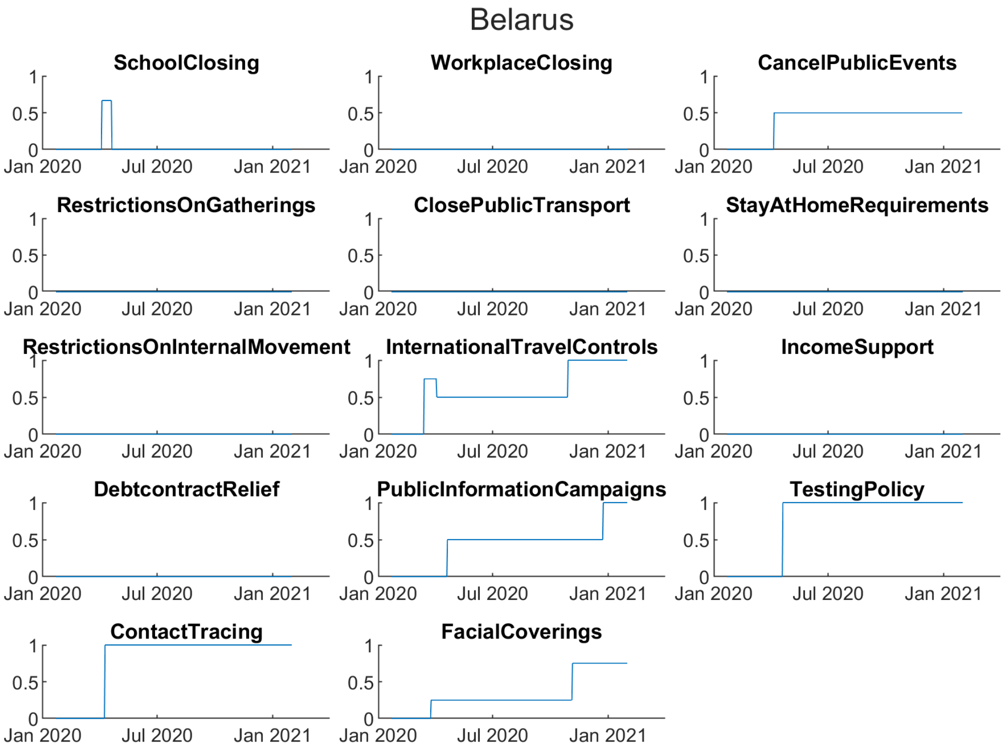

Supplementary Figure 4. The timeline of policies introduced to mitigate the spread of the pandemic — Belarus.

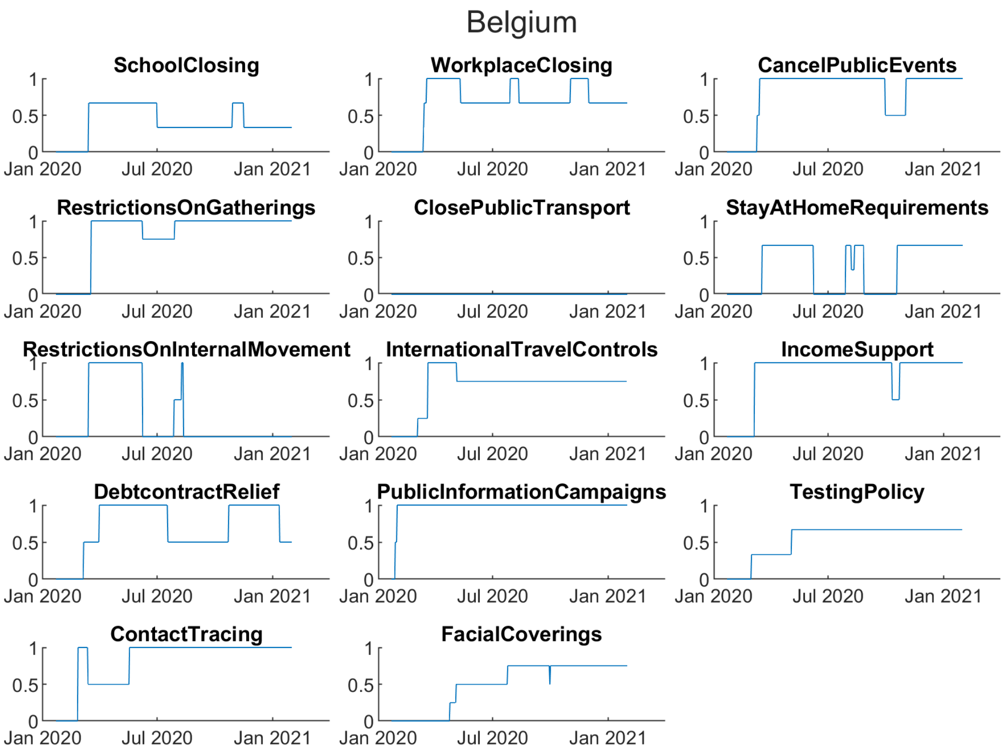

Supplementary Figure 5. The timeline of policies introduced to mitigate the spread of the pandemic — Belgium.

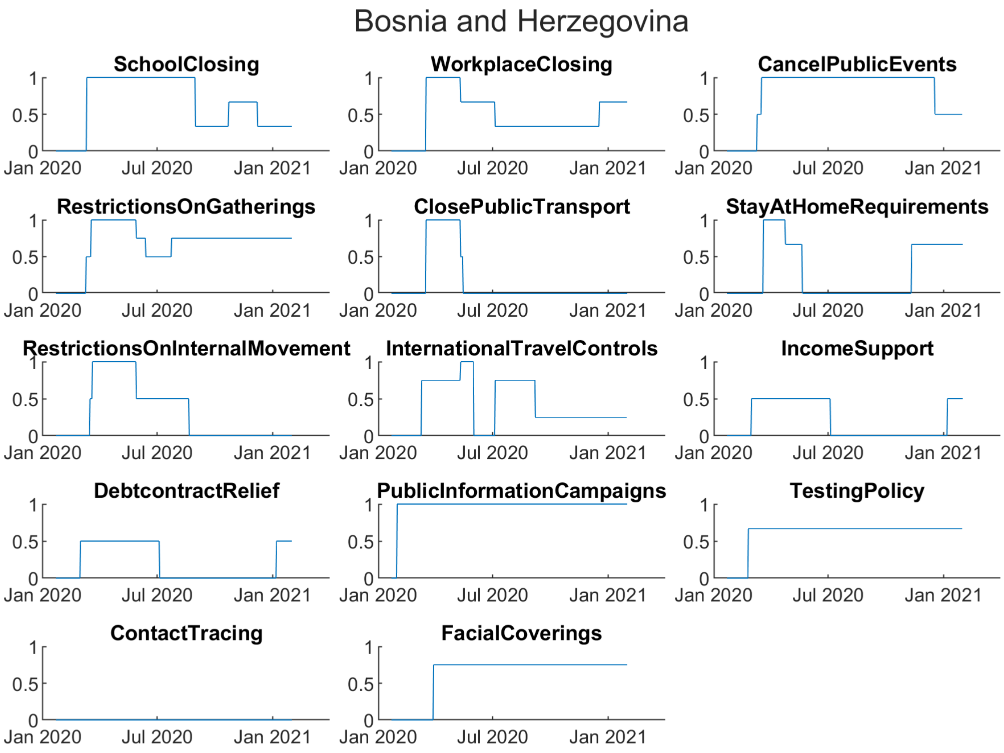

Supplementary Figure 6. The timeline of policies introduced to mitigate the spread of the pandemic — Bosnia and Herzegovina.

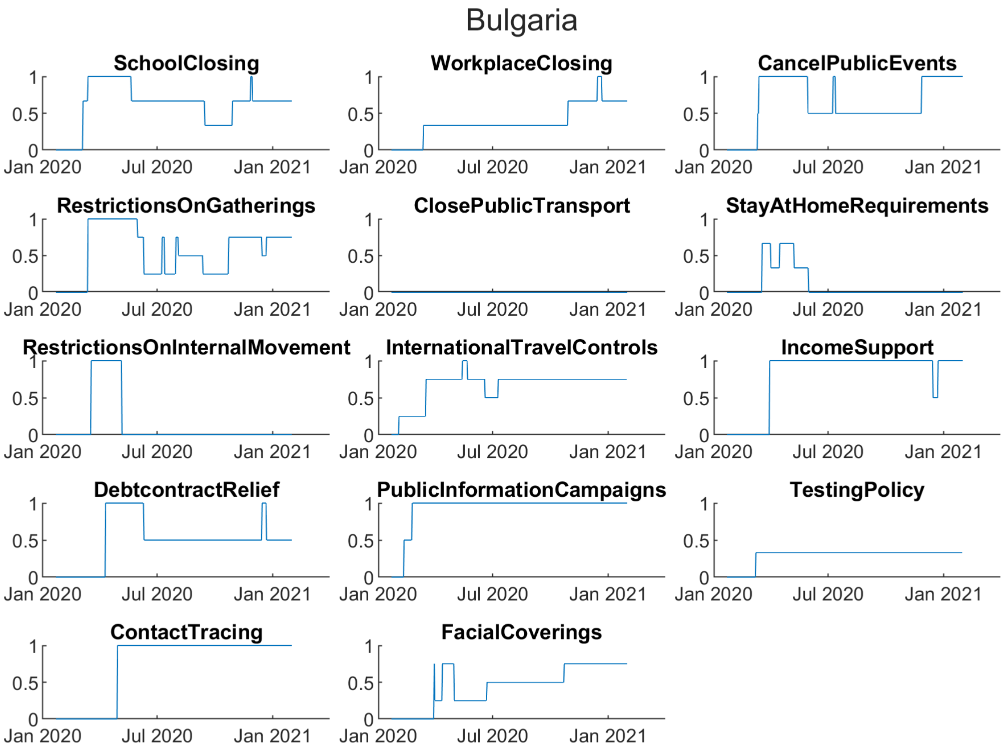

Supplementary Figure 7. The timeline of policies introduced to mitigate the spread of the pandemic — Bulgaria.

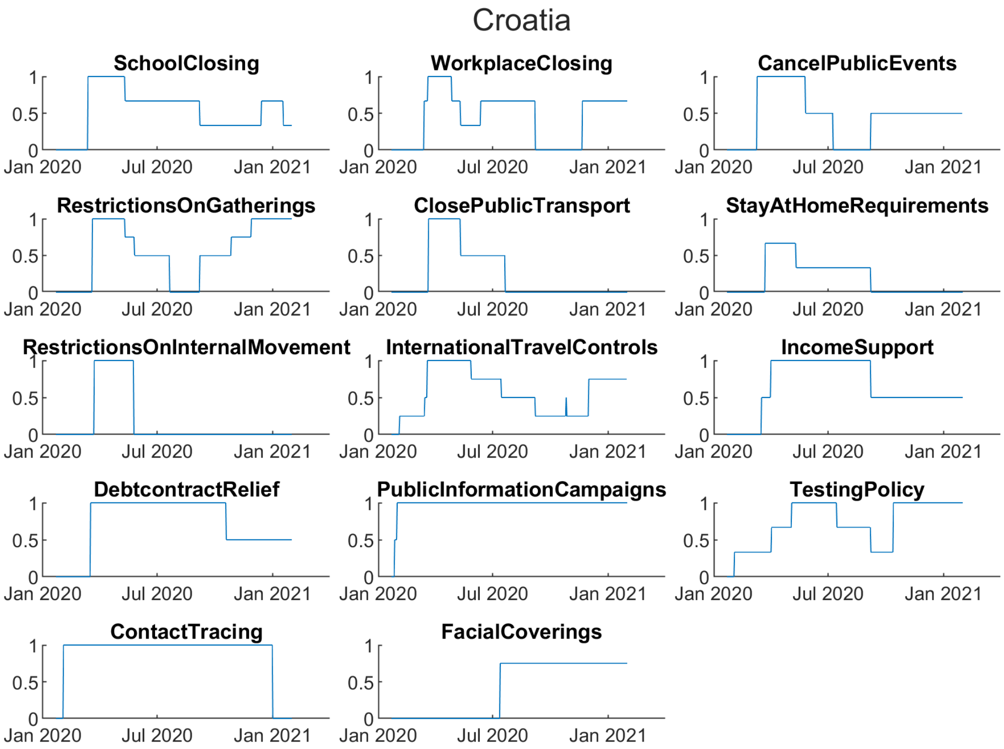

Supplementary Figure 8. The timeline of policies introduced to mitigate the spread of the pandemic — Croatia.

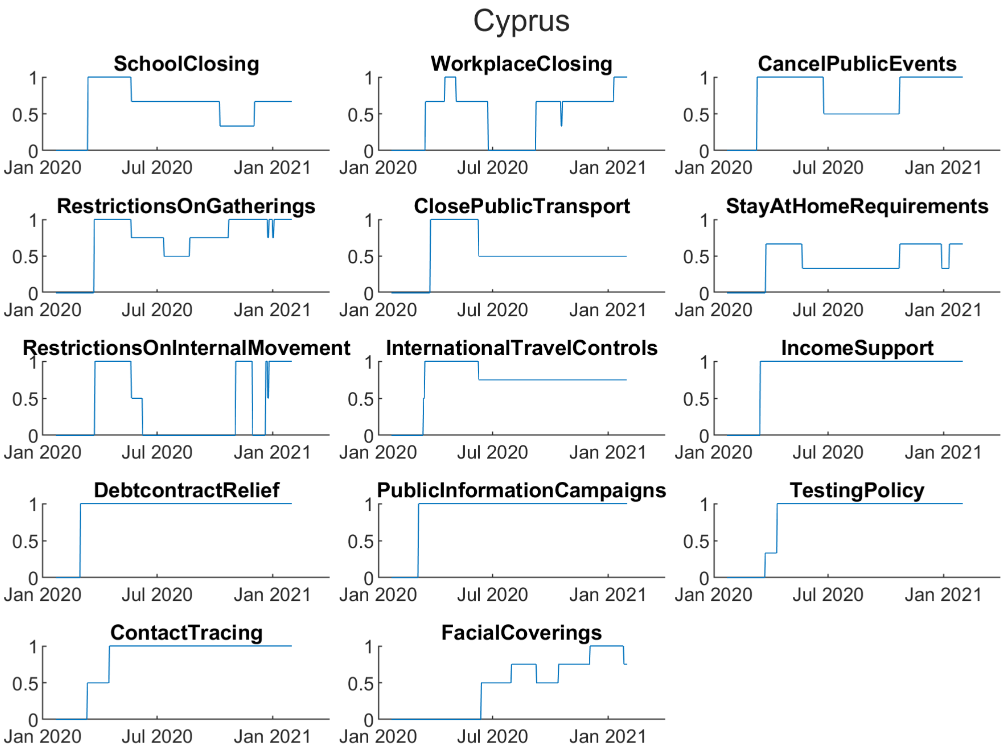

Supplementary Figure 9. The timeline of policies introduced to mitigate the spread of the pandemic — Cyprus.

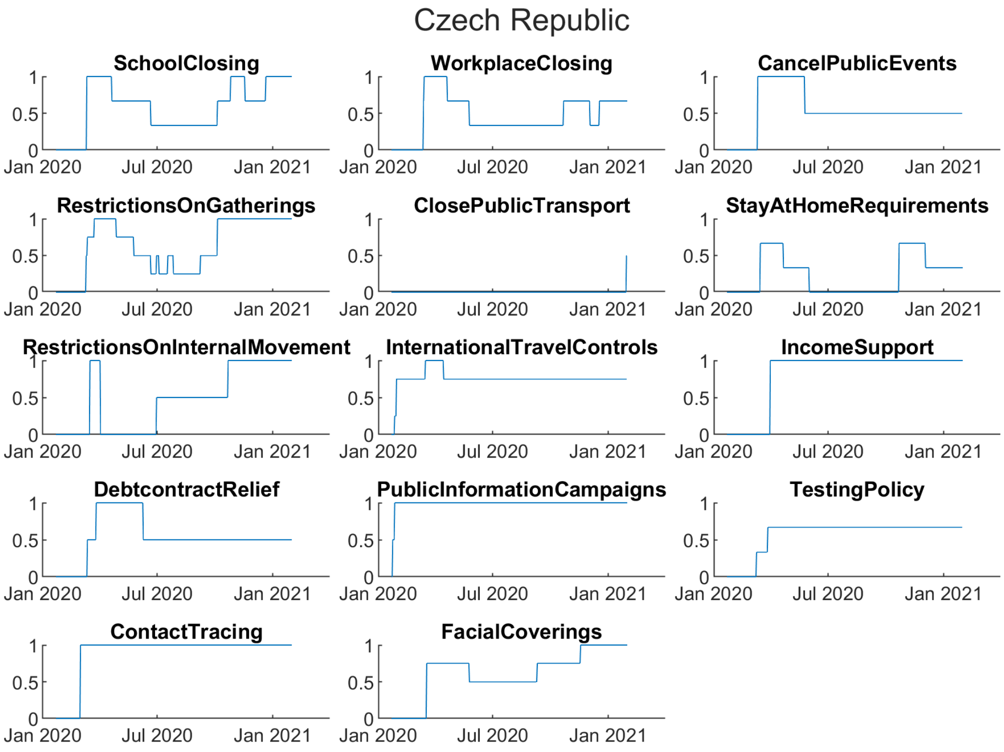

Supplementary Figure 10. The timeline of policies introduced to mitigate the spread of the pandemic — Czech Republic.

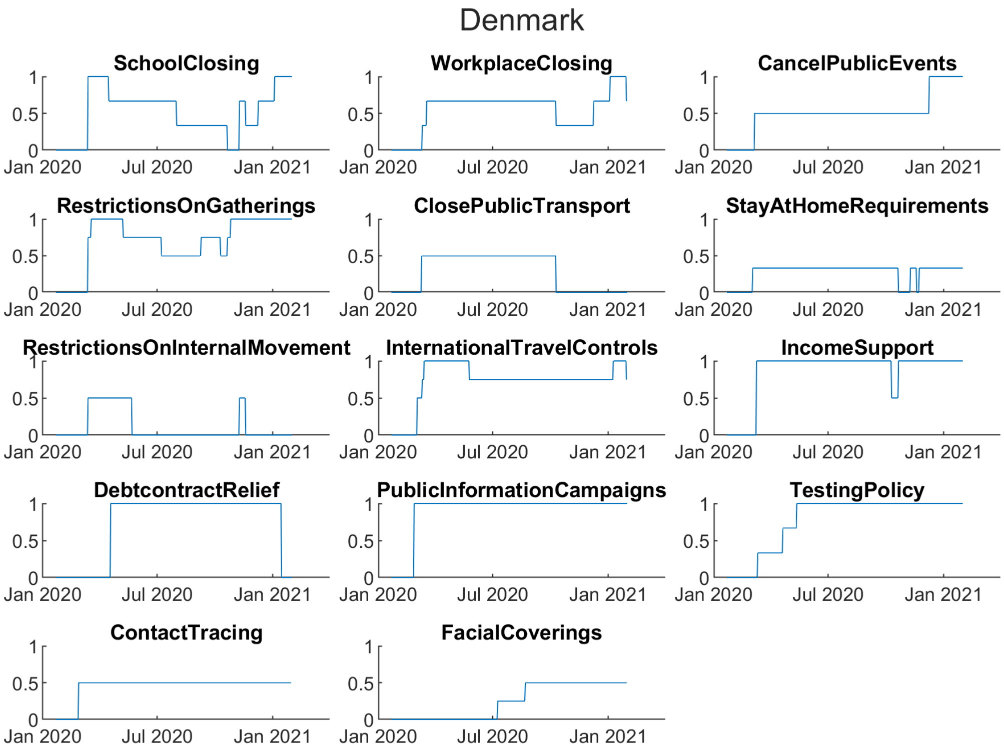

Supplementary Figure 11. The timeline of policies introduced to mitigate the spread of the pandemic — Denmark.

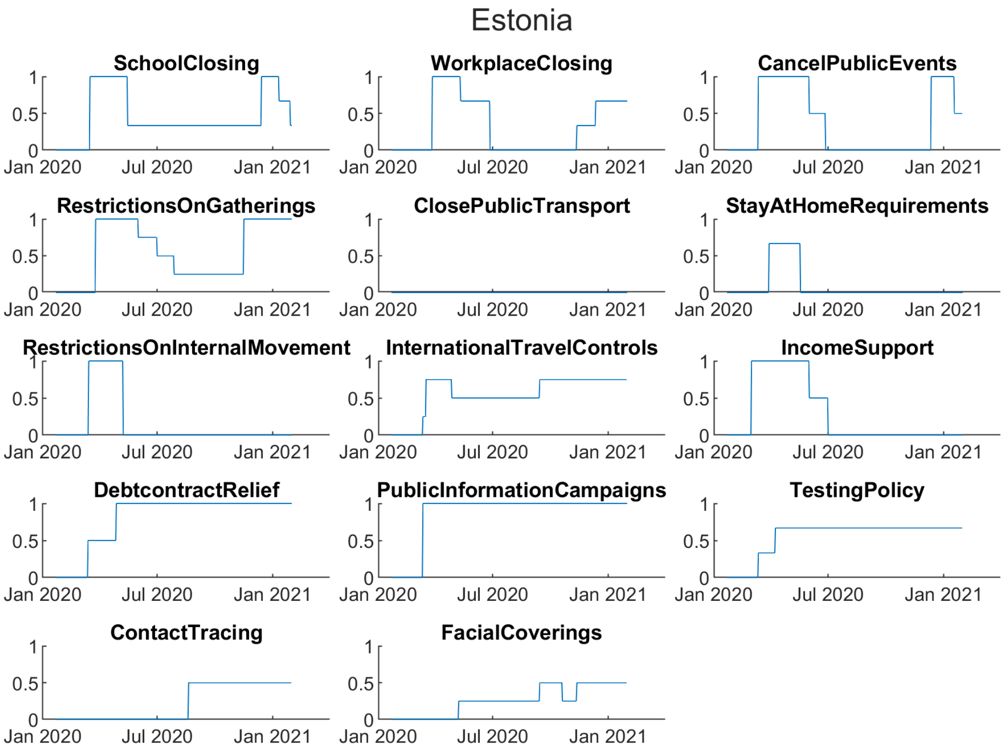

Supplementary Figure 12. The timeline of policies introduced to mitigate the spread of the pandemic — Estonia.

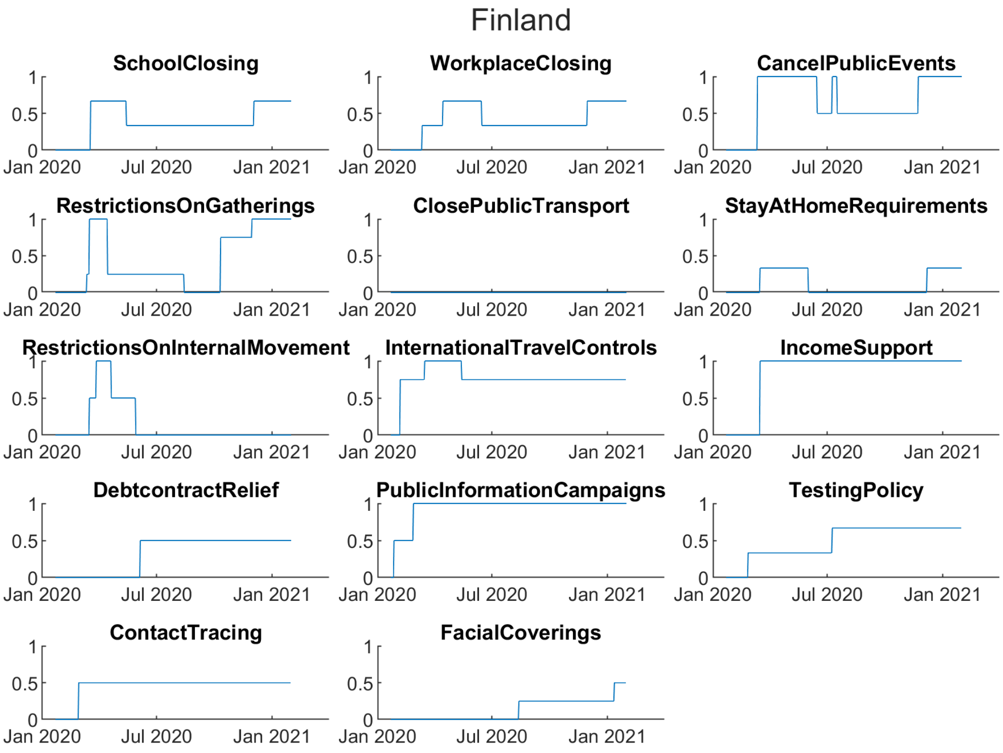

Supplementary Figure 13. The timeline of policies introduced to mitigate the spread of the pandemic — Finland.

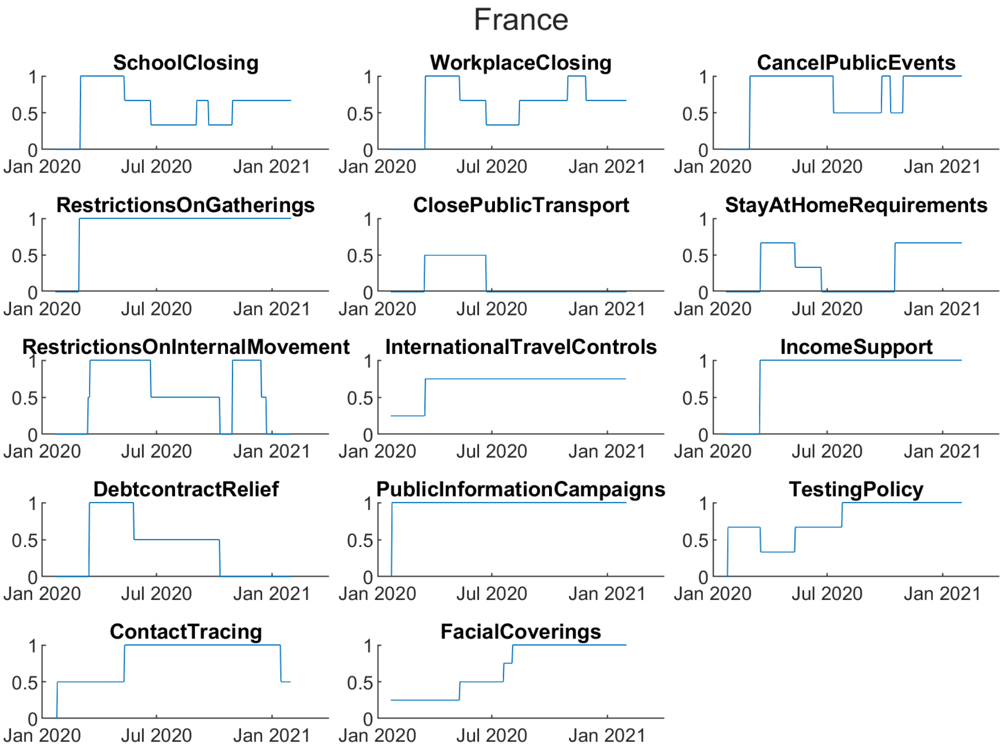

Supplementary Figure 14. The timeline of policies introduced to mitigate the spread of the pandemic — France.

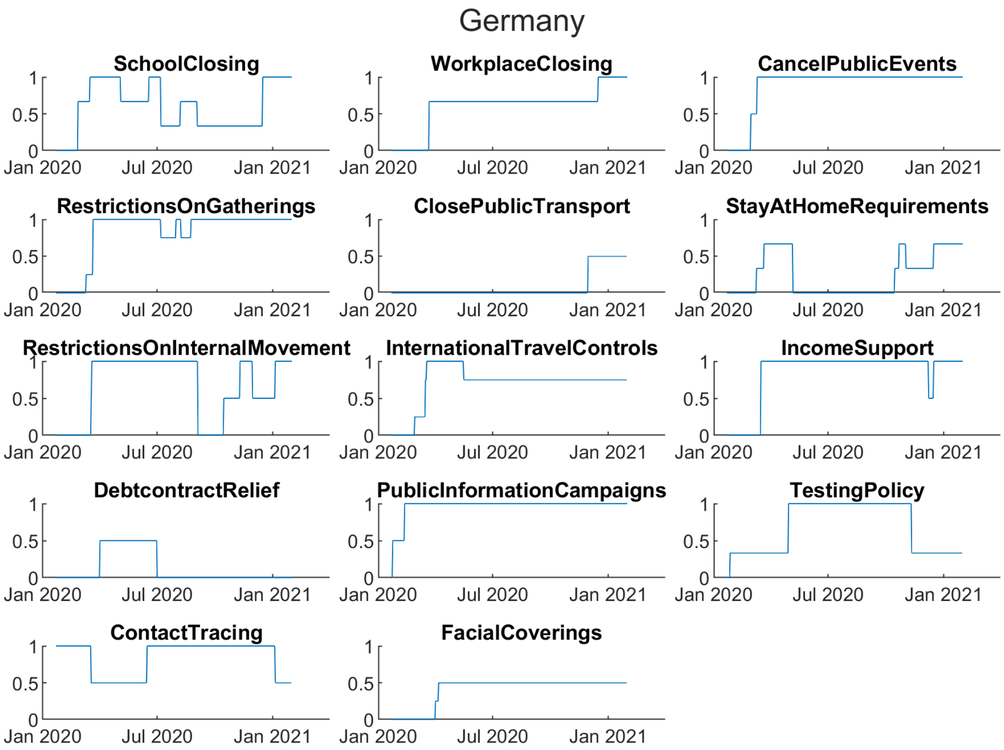

Supplementary Figure 15. The timeline of policies introduced to mitigate the spread of the pandemic — Germany.

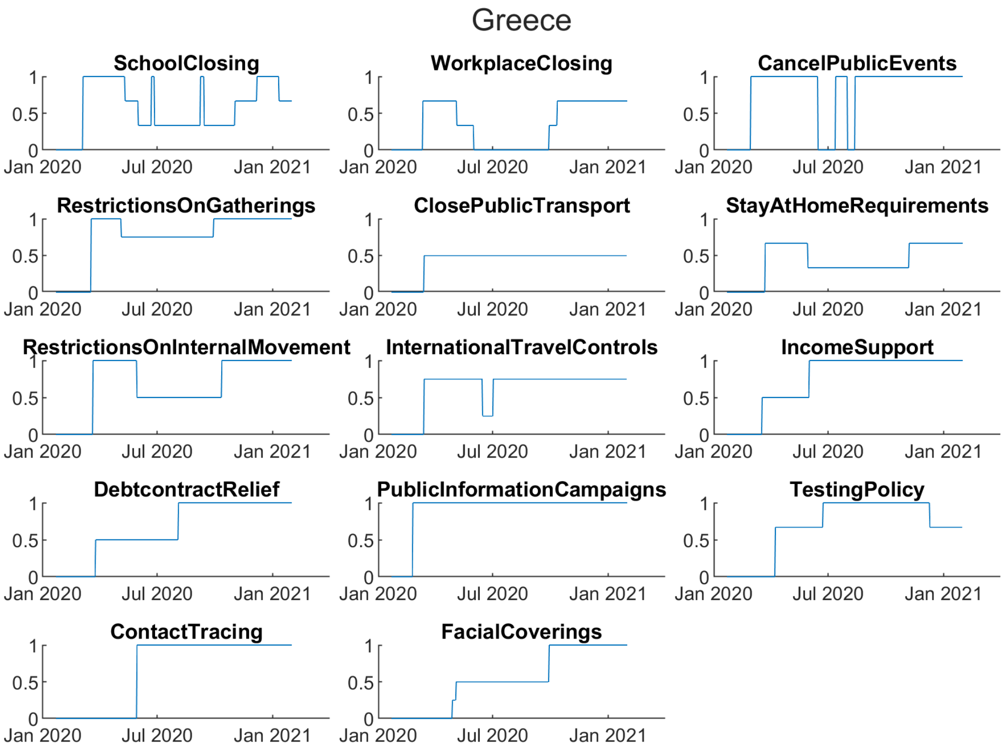

Supplementary Figure 16. The timeline of policies introduced to mitigate the spread of the pandemic — Greece.

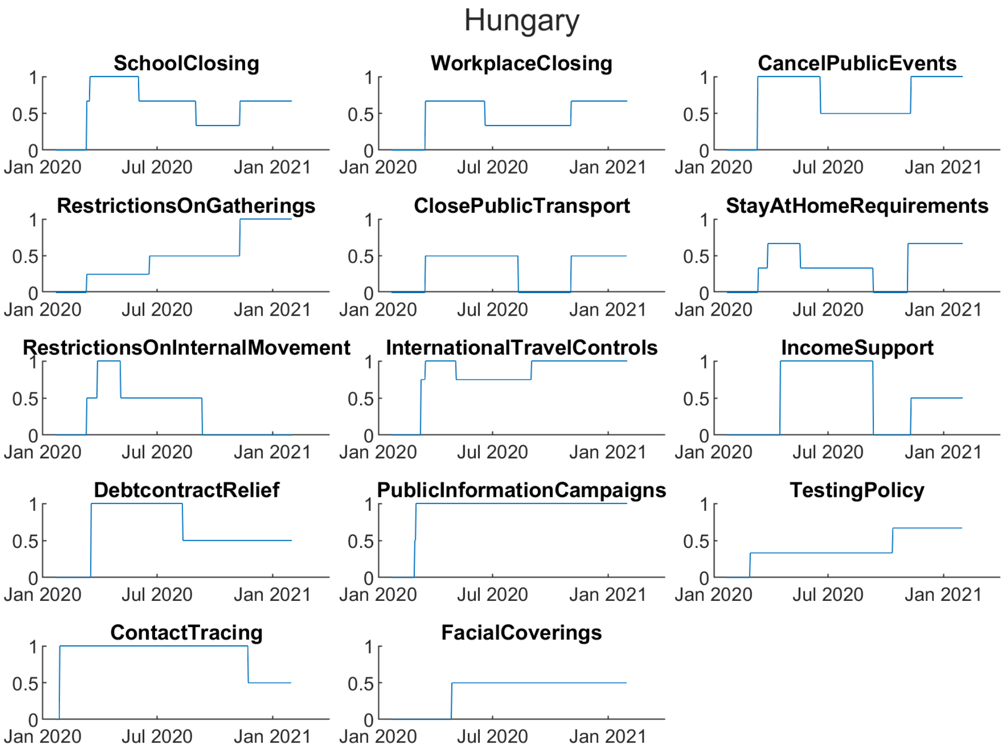

Supplementary Figure 17. The timeline of policies introduced to mitigate the spread of the pandemic — Hungary.

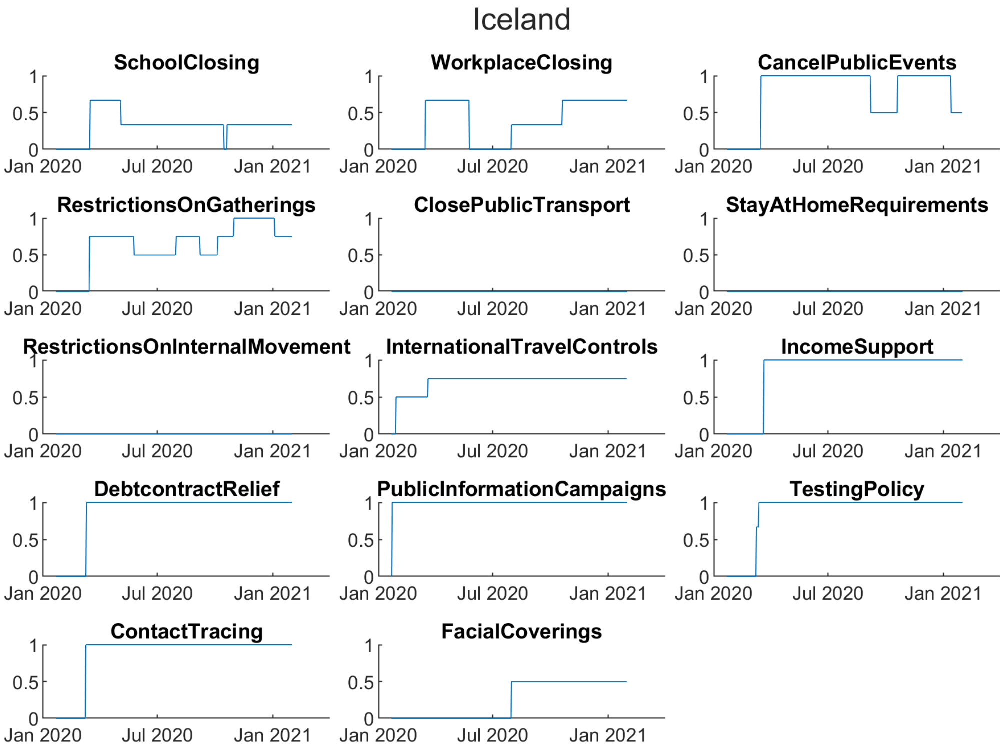

Supplementary Figure 18. The timeline of policies introduced to mitigate the spread of the pandemic — Iceland.

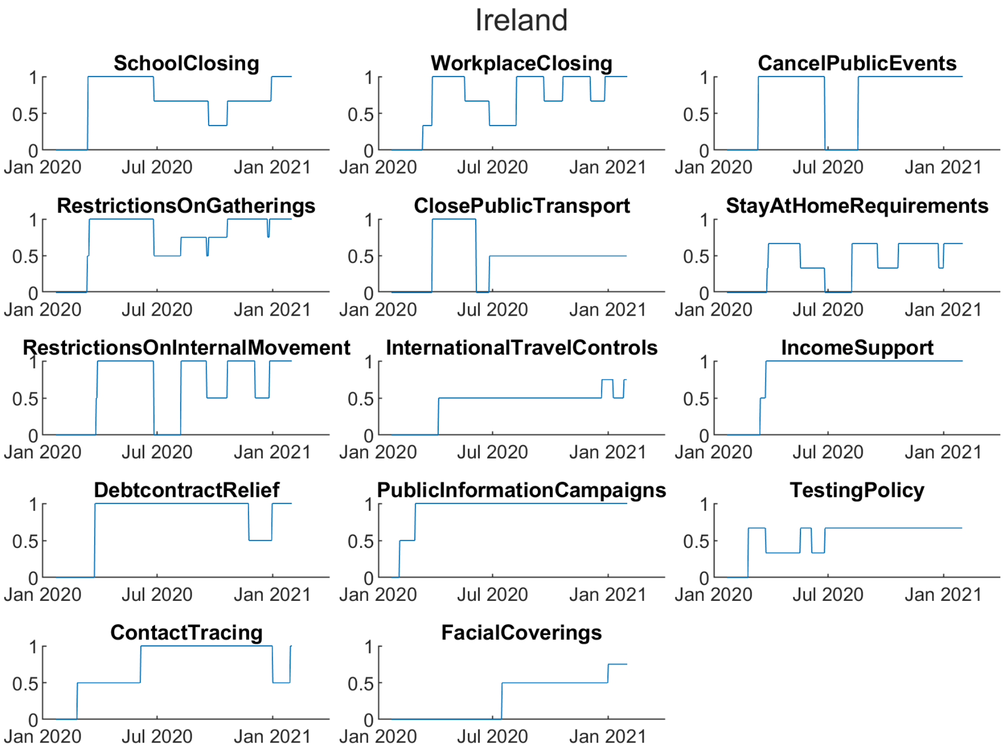

Supplementary Figure 19. The timeline of policies introduced to mitigate the spread of the pandemic — Ireland.

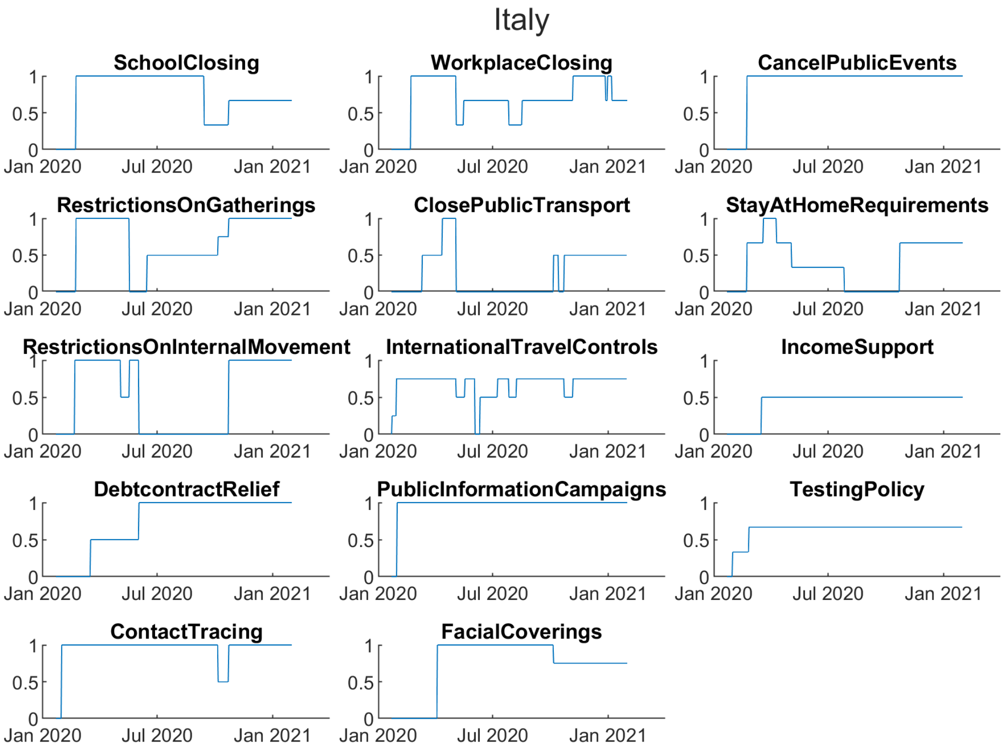

Supplementary Figure 20. The timeline of policies introduced to mitigate the spread of the pandemic — Italy.

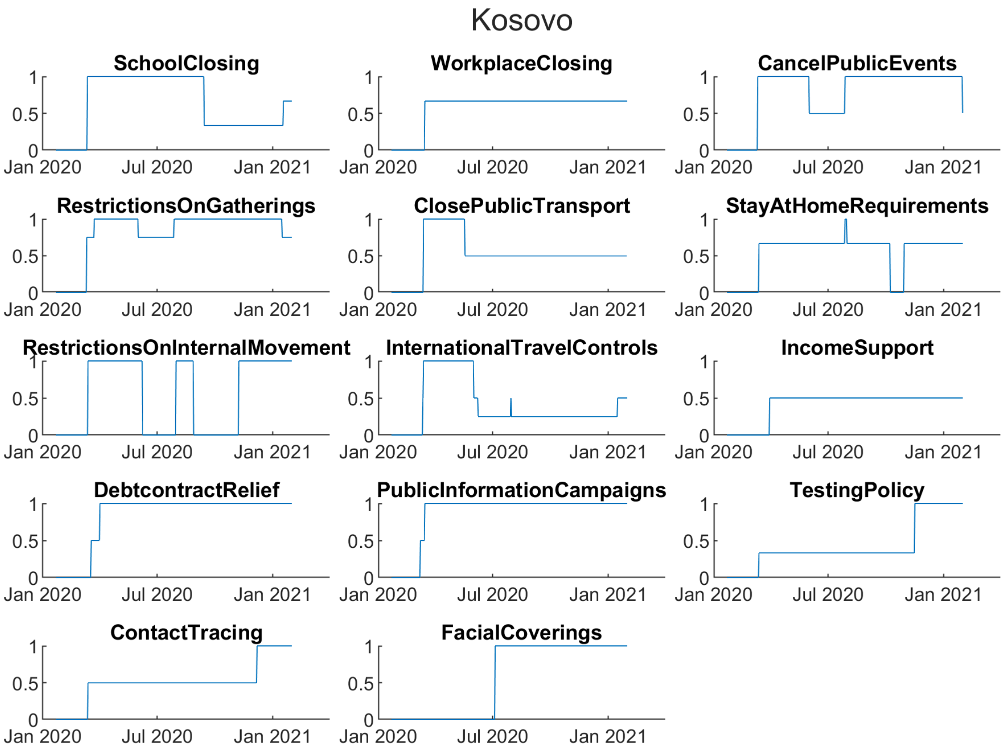

Supplementary Figure 21. The timeline of policies introduced to mitigate the spread of the pandemic — Kosovo.

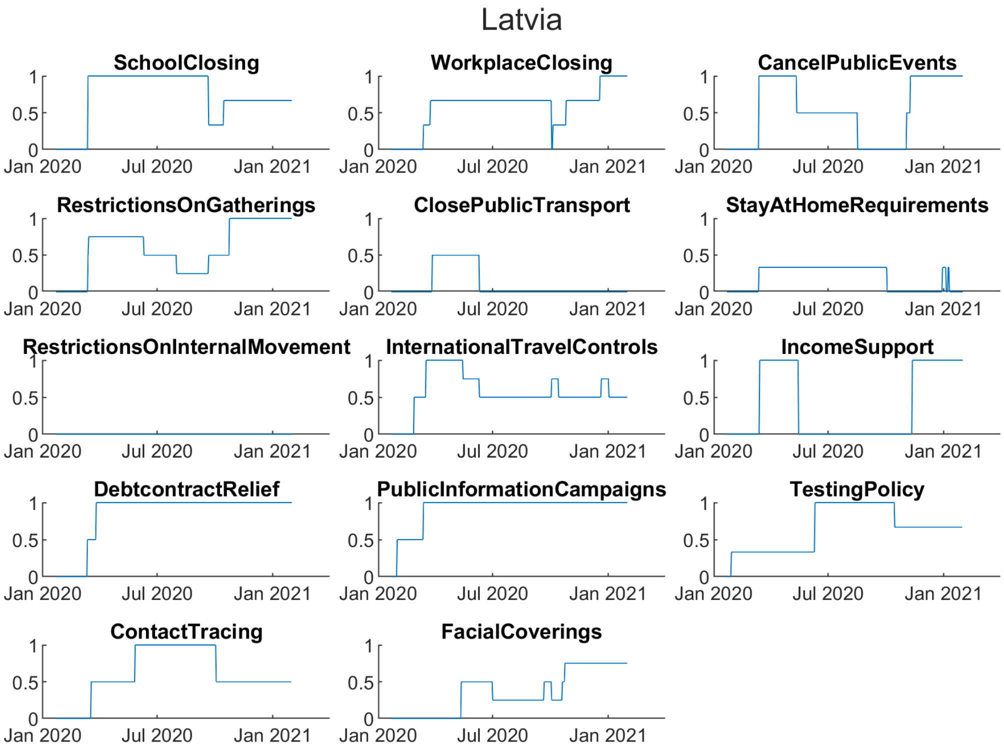

Supplementary Figure 22. The timeline of policies introduced to mitigate the spread of the pandemic — Latvia.

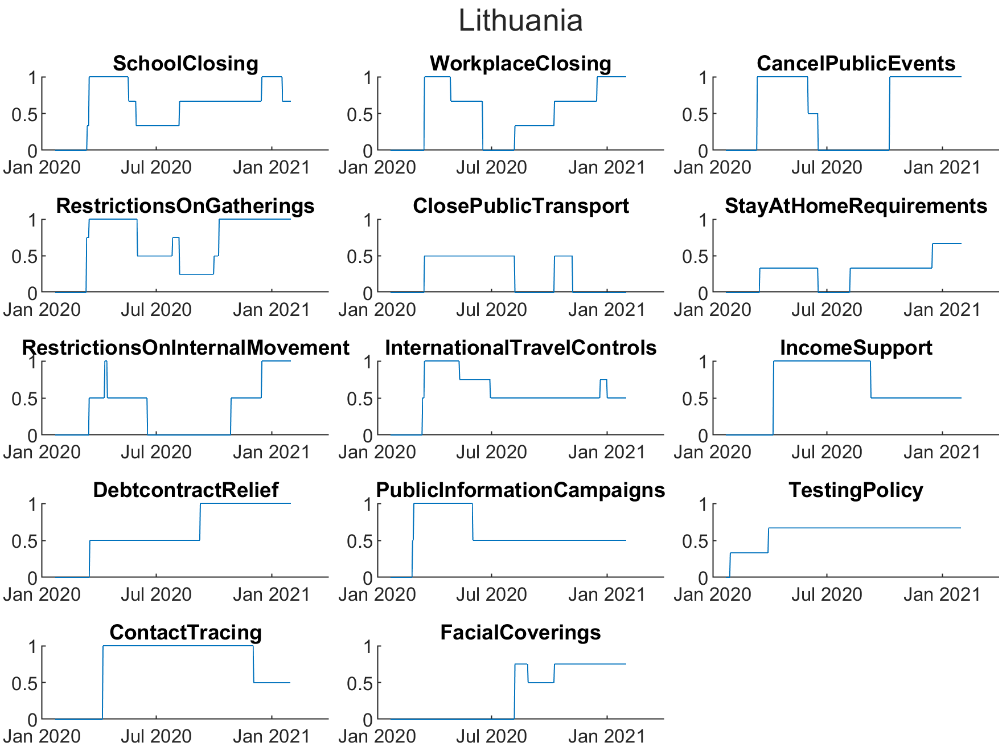

Supplementary Figure 23. The timeline of policies introduced to mitigate the spread of the pandemic — Lithuania.

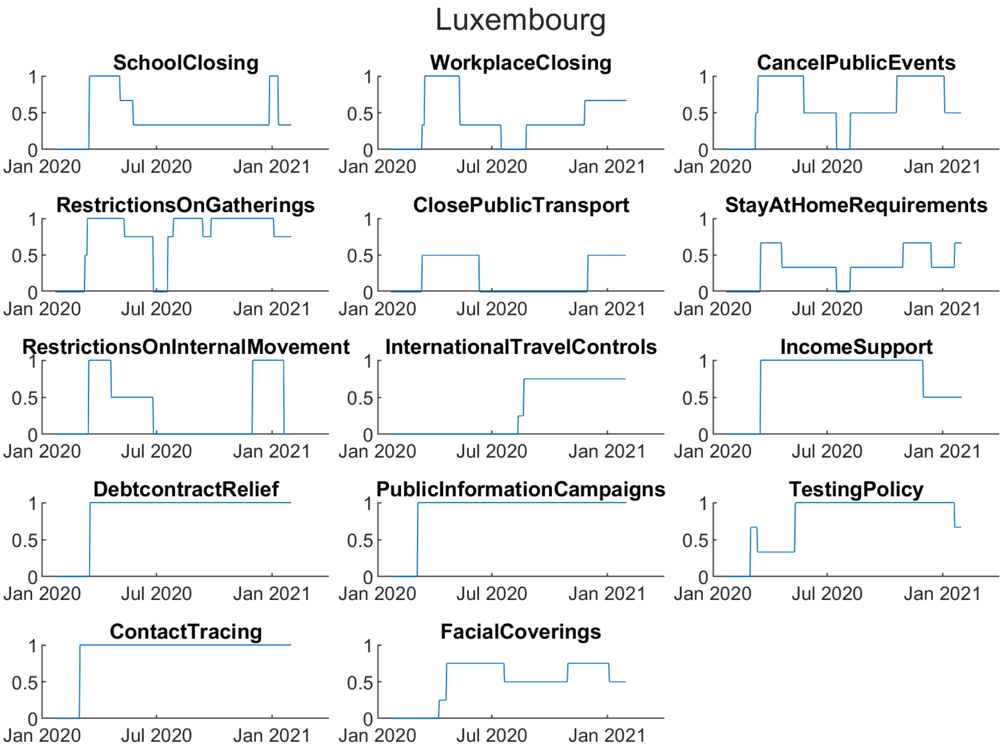

Supplementary Figure 24. The timeline of policies introduced to mitigate the spread of the pandemic — Luxembourg.

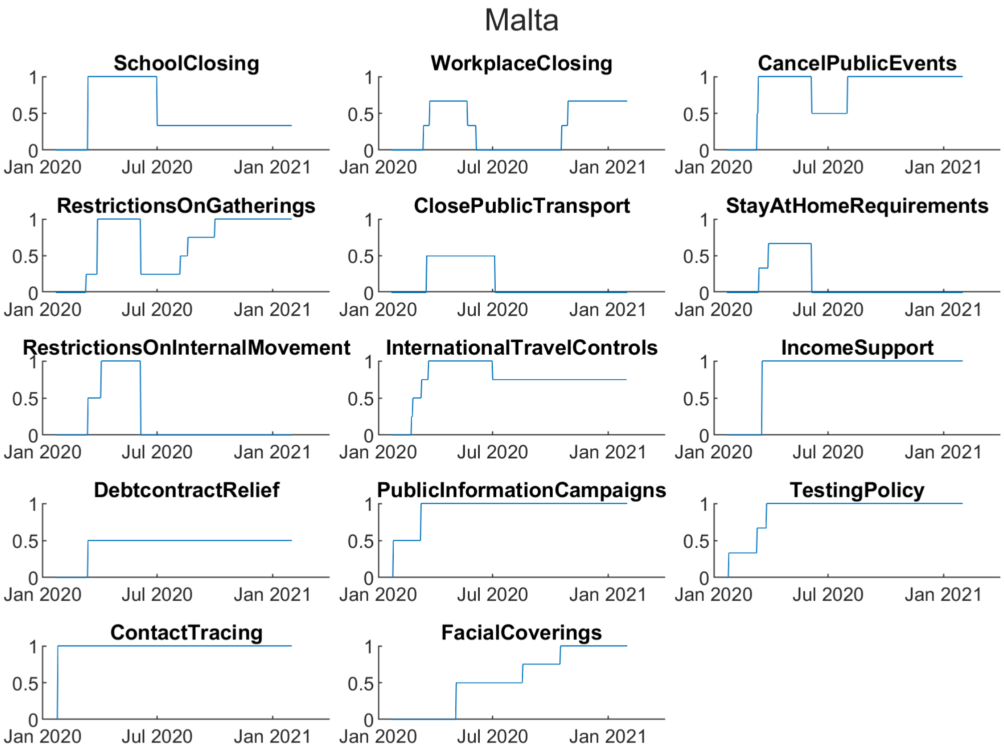

Supplementary Figure 25. The timeline of policies introduced to mitigate the spread of the pandemic — Malta.

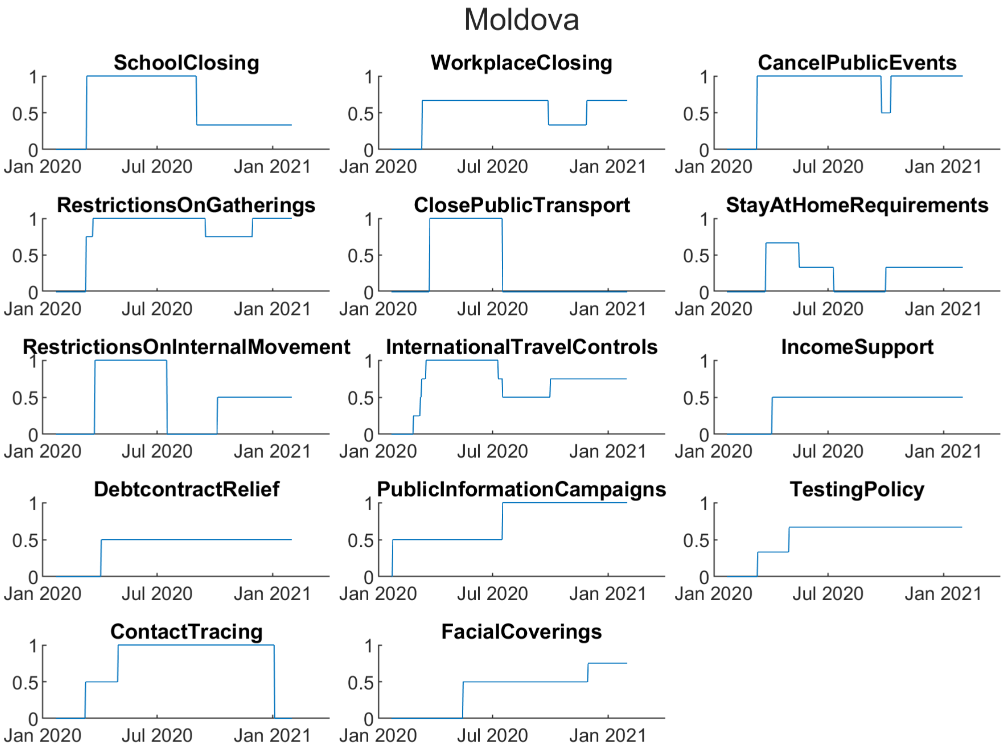

Supplementary Figure 26. The timeline of policies introduced to mitigate the spread of the pandemic — Moldova.

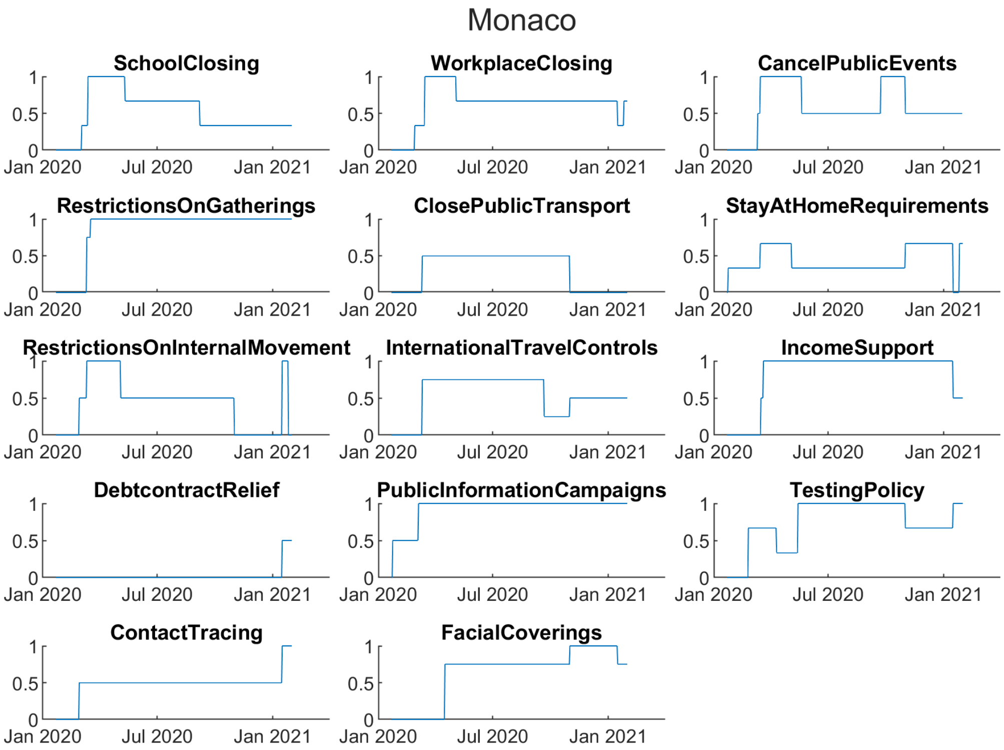

Supplementary Figure 27. The timeline of policies introduced to mitigate the spread of the pandemic — Monaco.

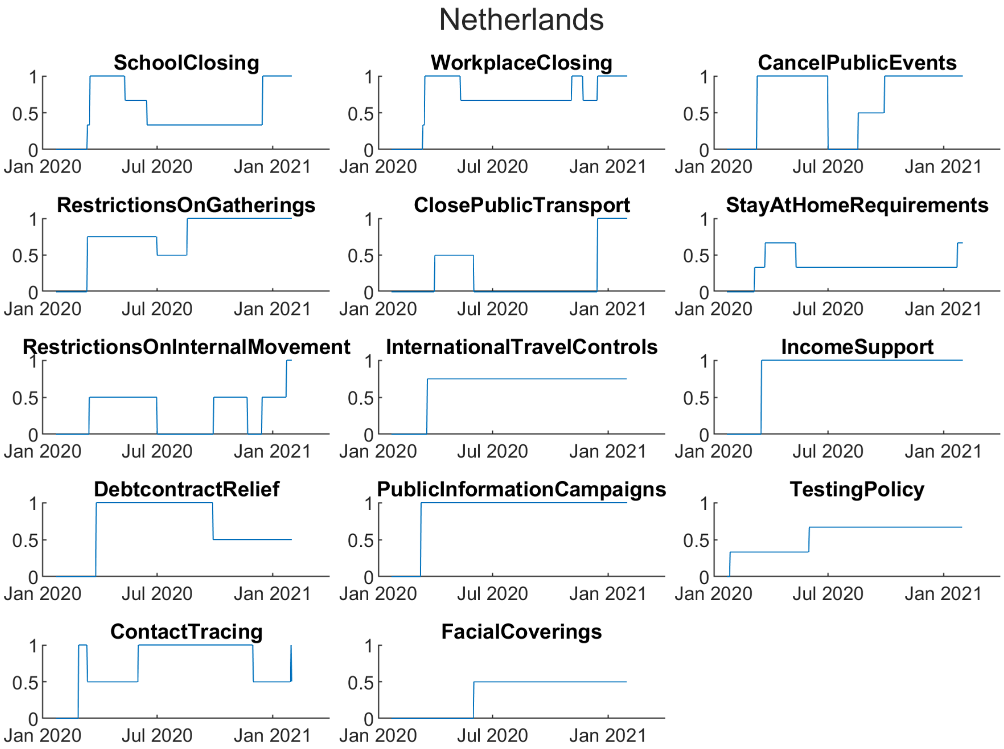

Supplementary Figure 28. The timeline of policies introduced to mitigate the spread of the pandemic — Netherlands.

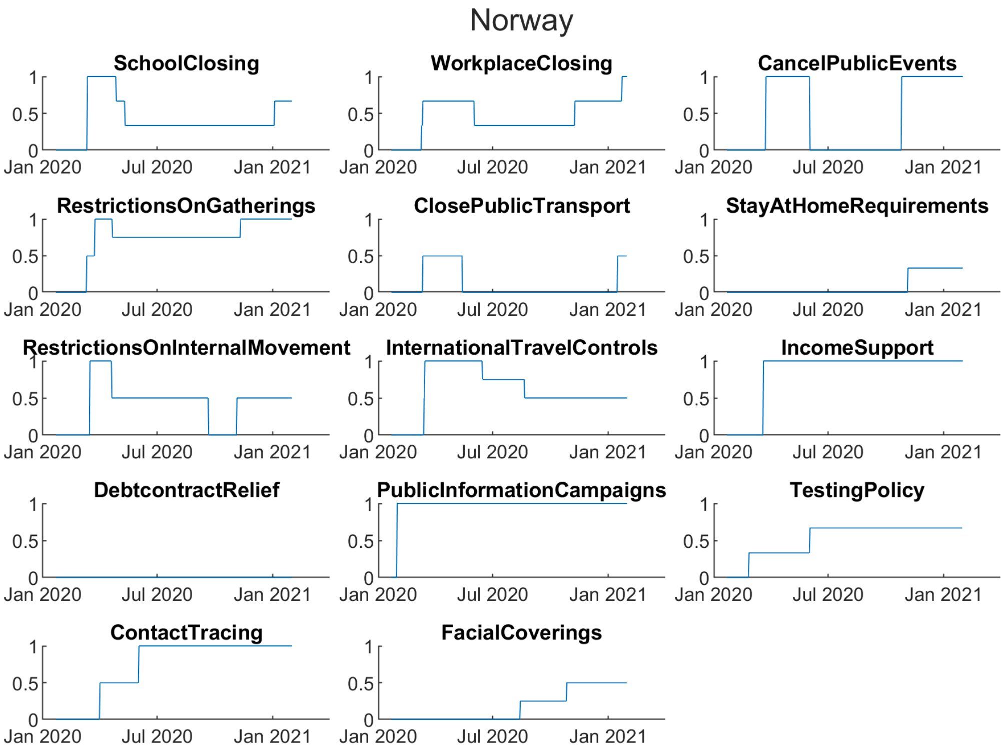

Supplementary Figure 29. The timeline of policies introduced to mitigate the spread of the pandemic — Norway.

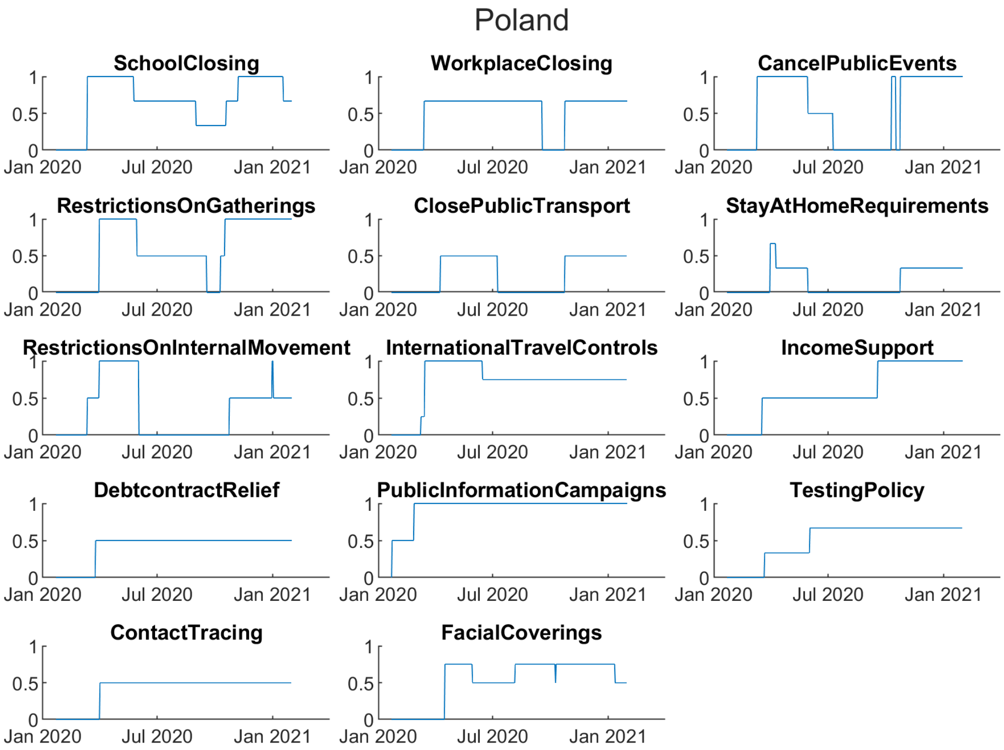

Supplementary Figure 30. The timeline of policies introduced to mitigate the spread of the pandemic — Poland.

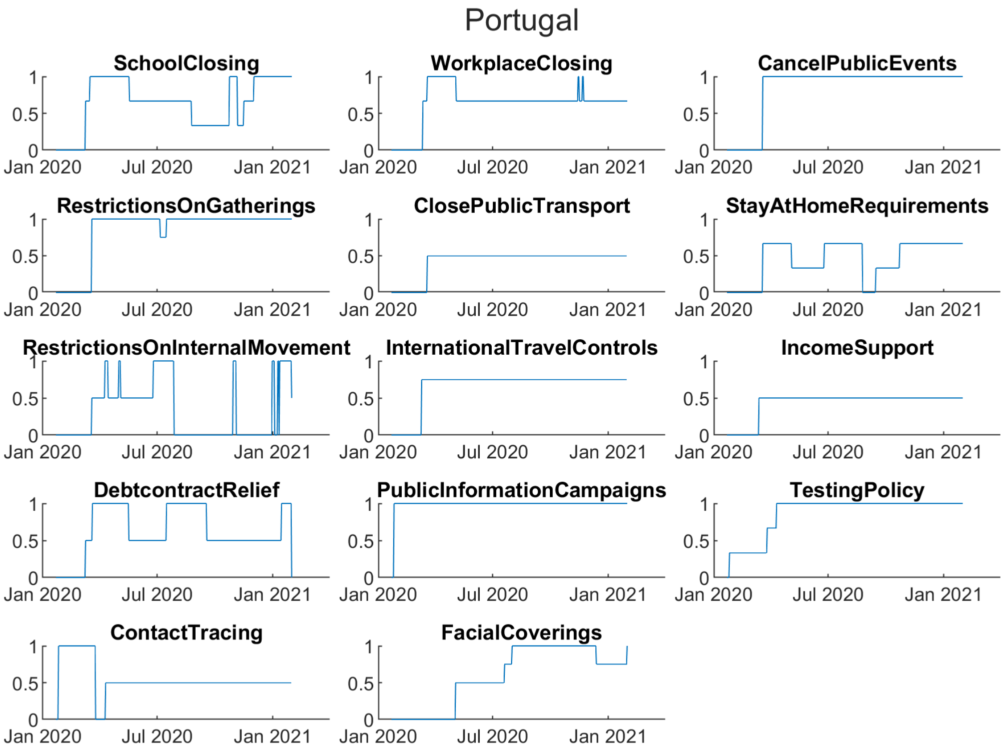

Supplementary Figure 31. The timeline of policies introduced to mitigate the spread of the pandemic — Portugal.

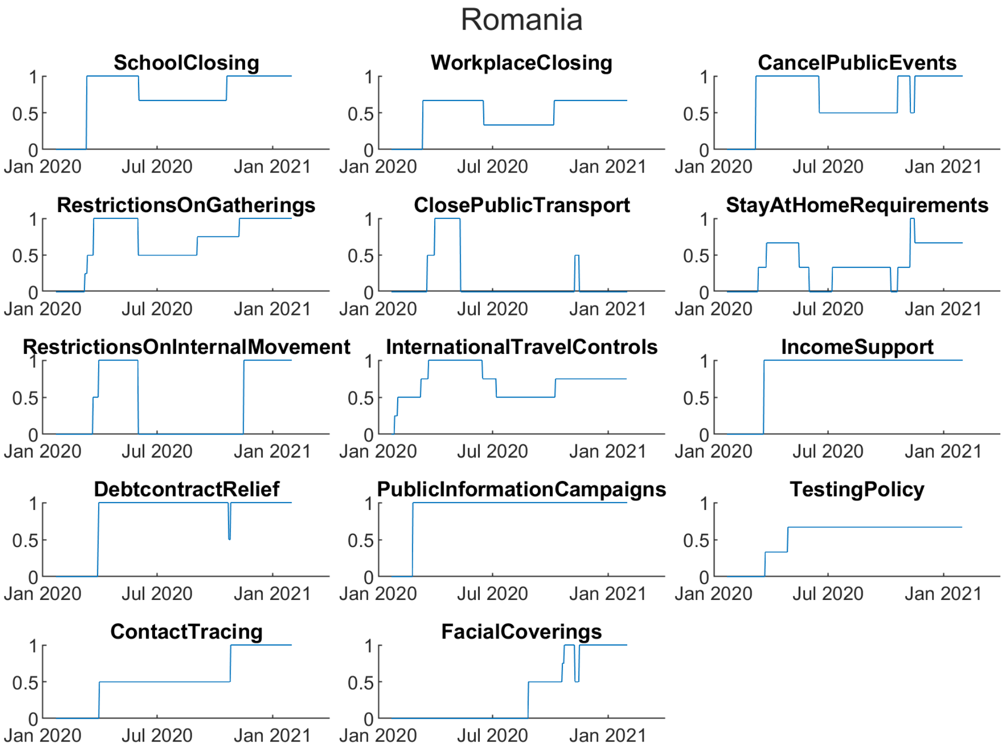

Supplementary Figure 32. The timeline of policies introduced to mitigate the spread of the pandemic — Romania.

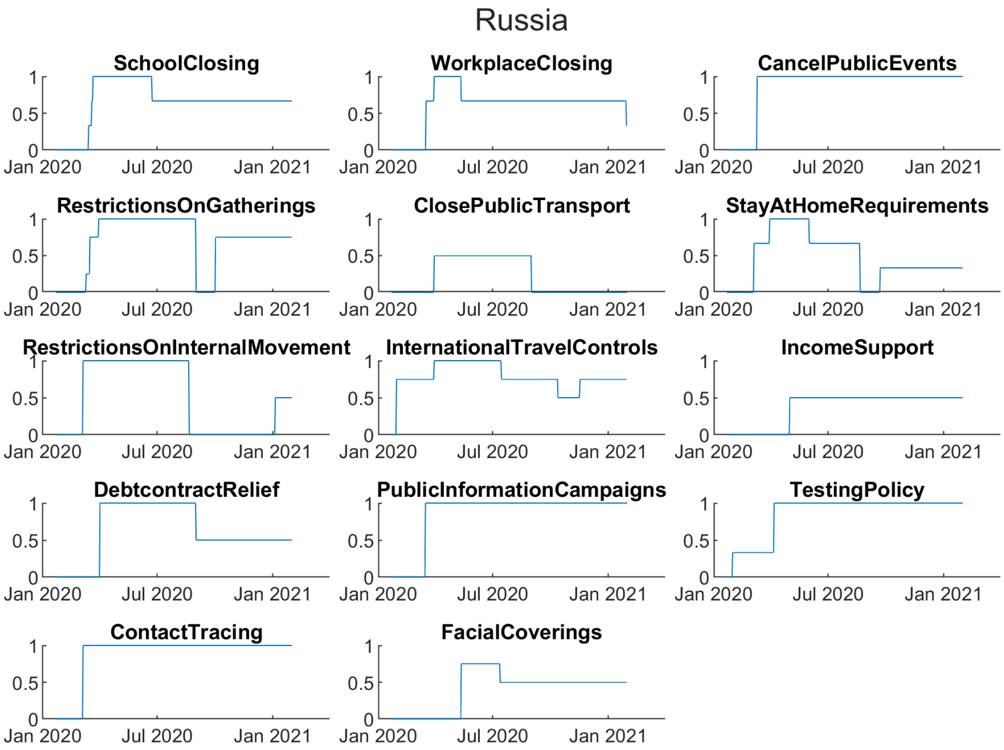

Supplementary Figure 33. The timeline of policies introduced to mitigate the spread of the pandemic — Russia.

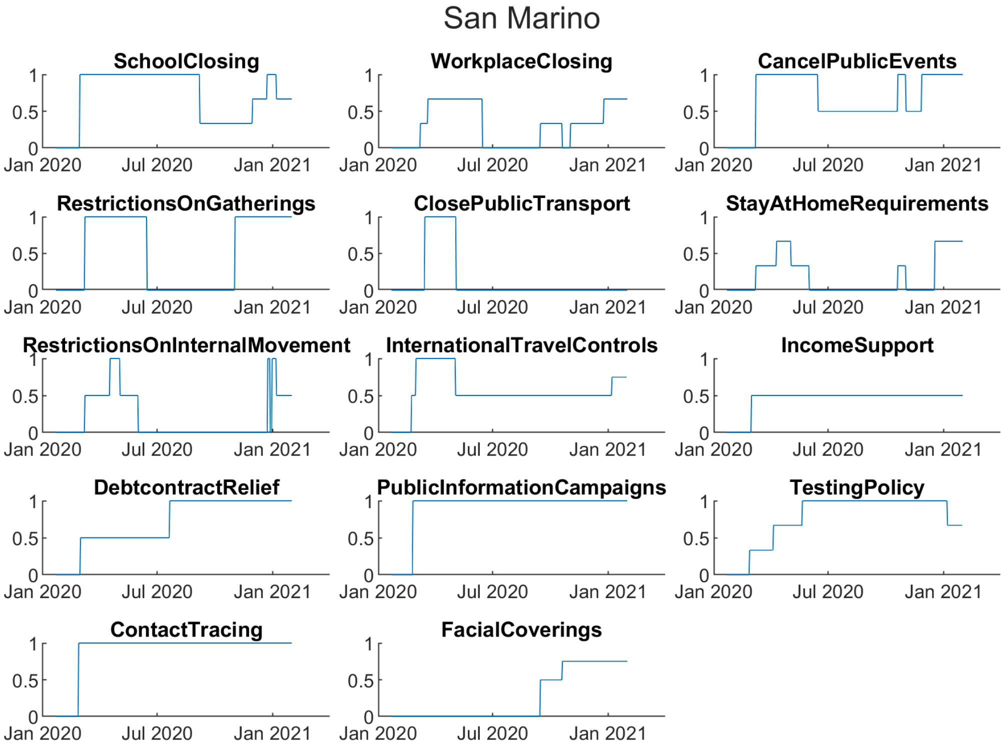

Supplementary Figure 34. The timeline of policies introduced to mitigate the spread of the pandemic — San Marino.

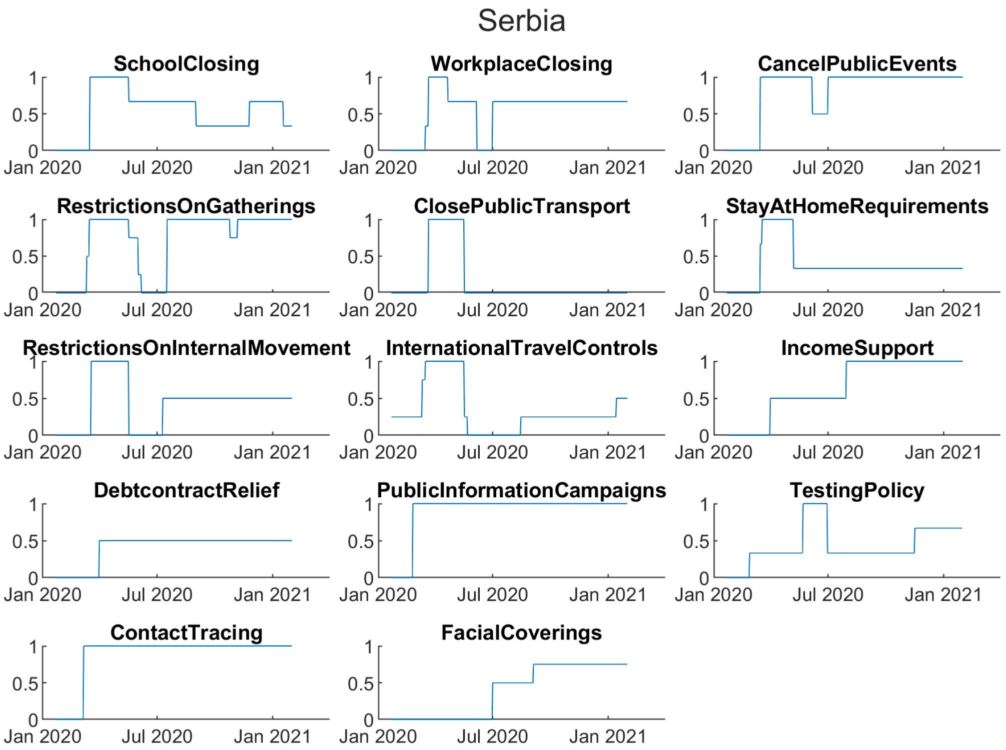

Supplementary Figure 35. The timeline of policies introduced to mitigate the spread of the pandemic — Serbia.

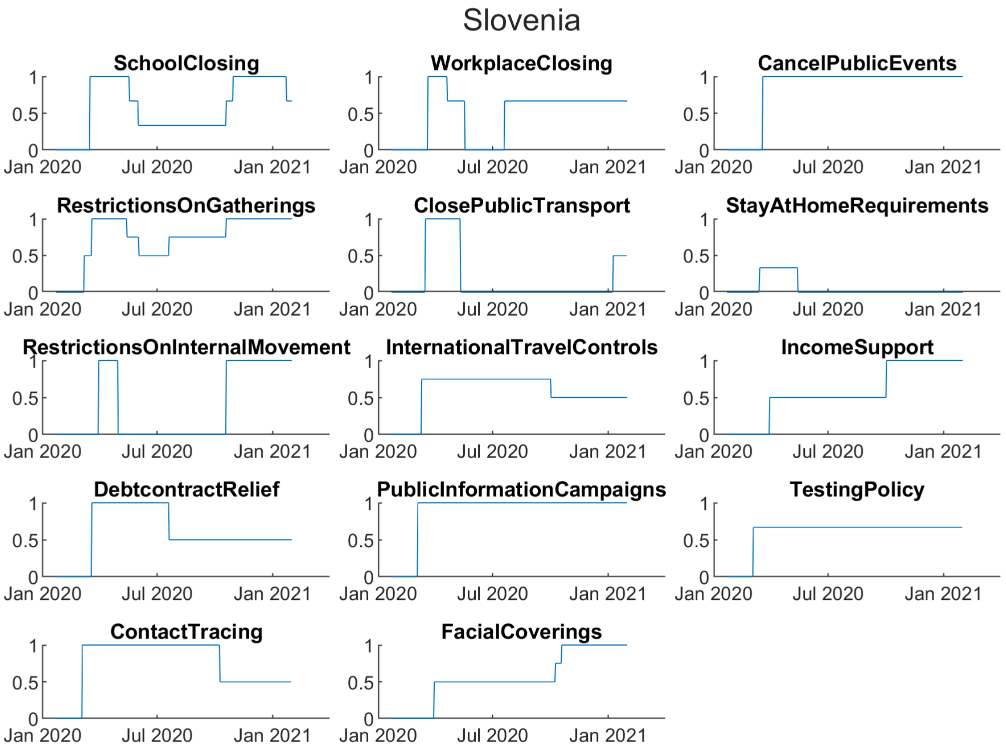

Supplementary Figure 36. The timeline of policies introduced to mitigate the spread of the pandemic — Slovenia.

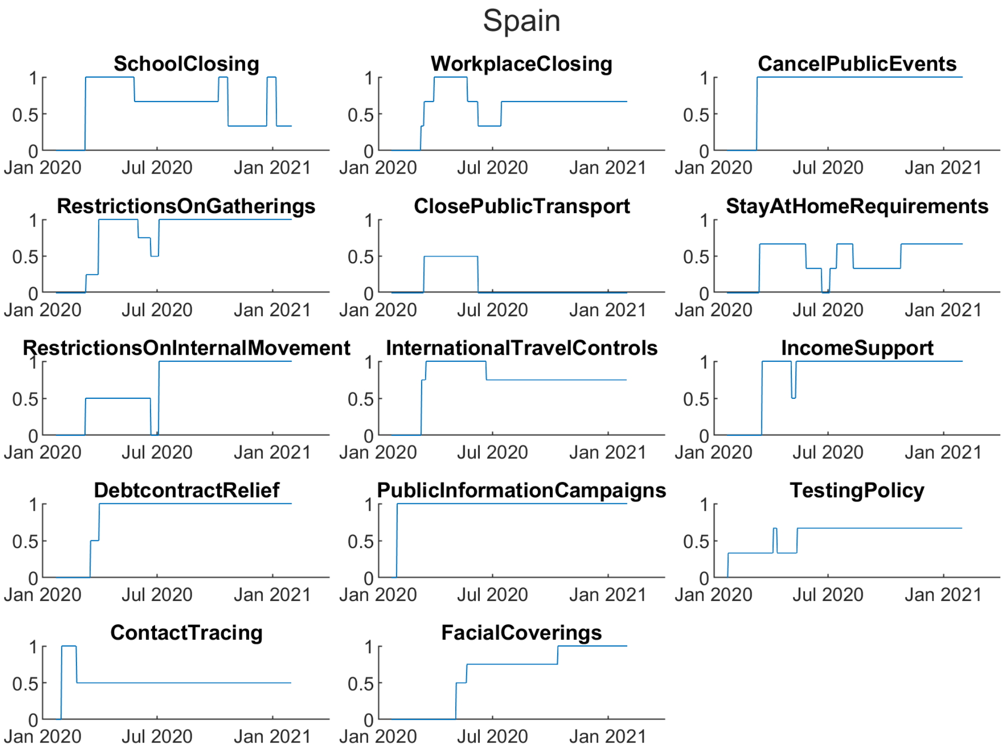

Supplementary Figure 37. The timeline of policies introduced to mitigate the spread of the pandemic — Spain.

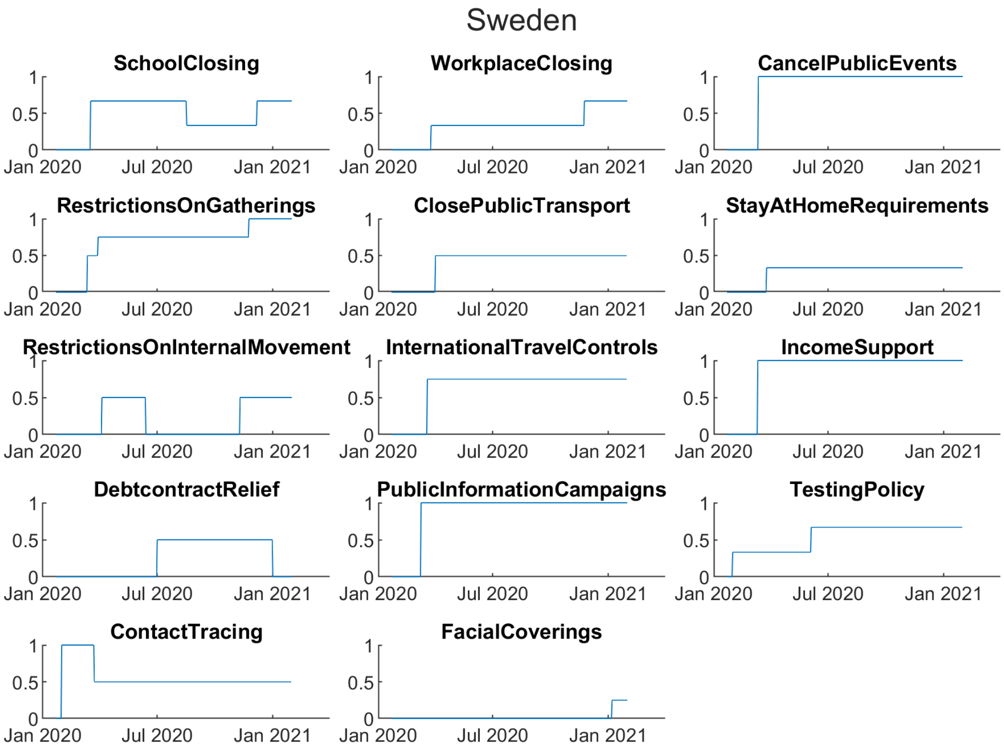

Supplementary Figure 38. The timeline of policies introduced to mitigate the spread of the pandemic — Sweden.

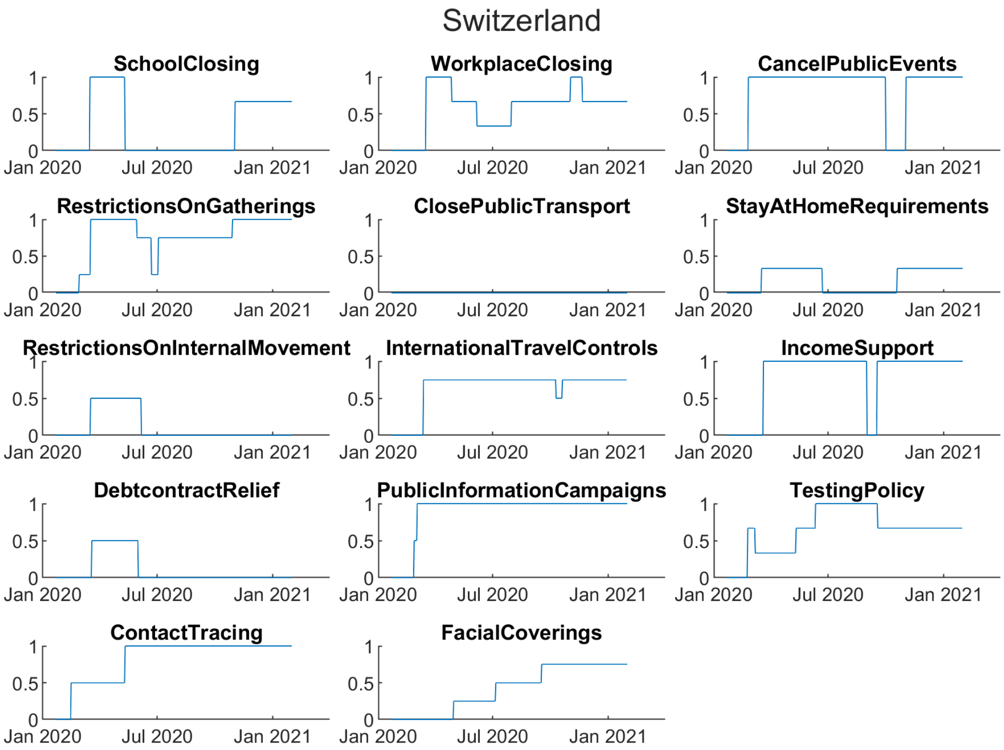

Supplementary Figure 39. The timeline of policies introduced to mitigate the spread of the pandemic — Switzerland.

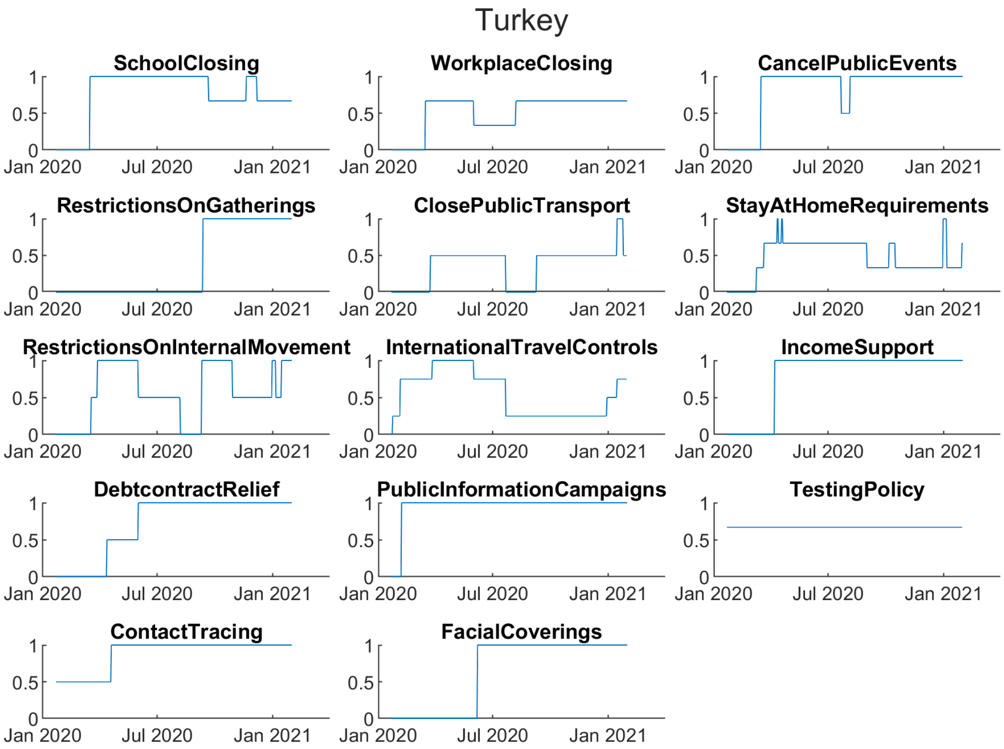

Supplementary Figure 40. The timeline of policies introduced to mitigate the spread of the pandemic — Turkey.

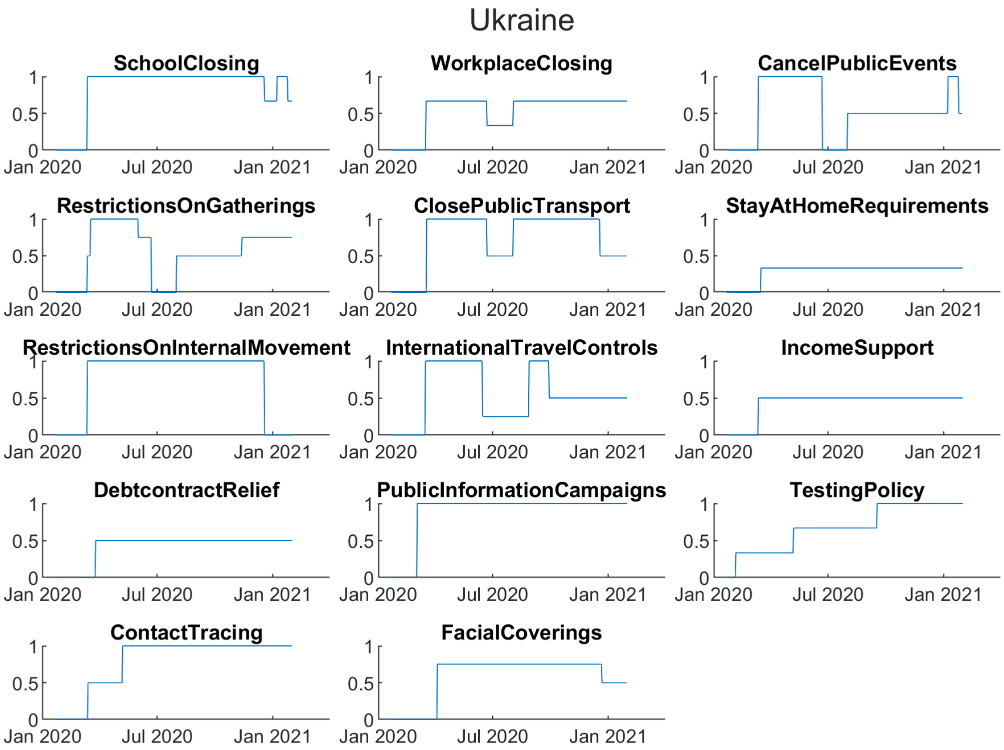

Supplementary Figure 41. The timeline of policies introduced to mitigate the spread of the pandemic — Ukraine.

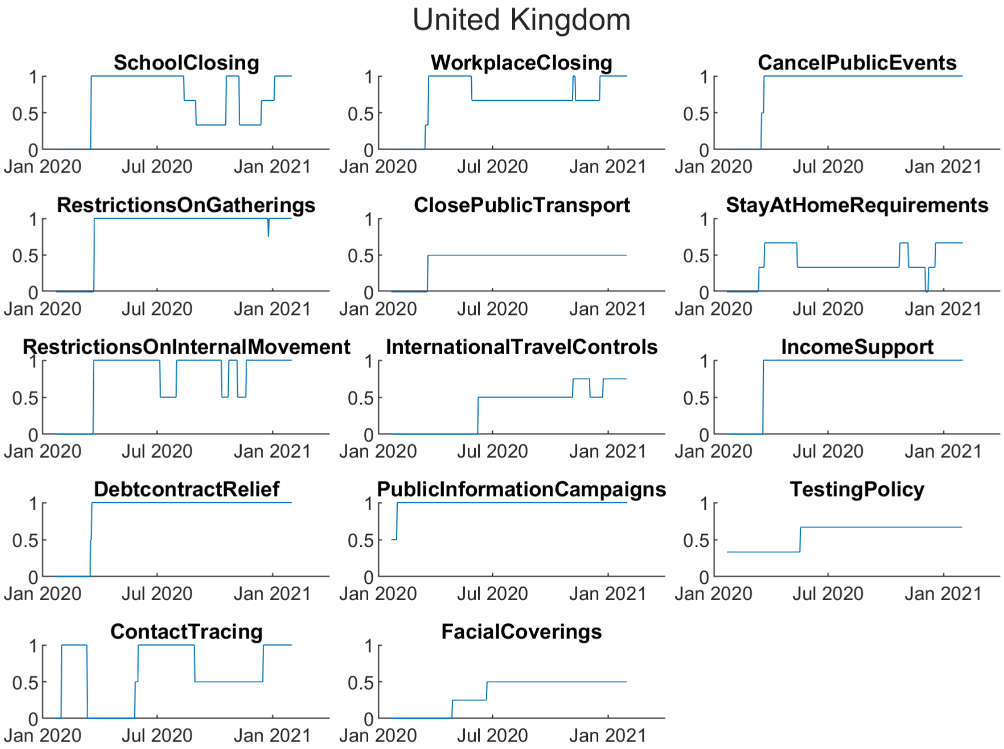

Supplementary Figure 42. The timeline of policies introduced to mitigate the spread of the pandemic - United Kingdom.

Independent models														
**Country**	a_1	a_2	a_3	a_4	a_5	a_6	a_7	a_8	a_9	a_{10}	a_{11}	a_{12}	a_{13}	b_k
Albania	0.24	-0.18	-0.06	-0.33	-0.14	0.58	-0.16	0.16	-0.01	0.62	-0.07	0.20	-0.06	0.34
Andorra	0.07	0.43	0.45	-0.93	-0.32	-2.13	0	0.27	-0.05	0.243	0.37	-0.24	0.51	0.25
Austria	0.12	0.20	0.05	-0.28	0.17	-0.02	-0.09	0.18	0.19	0.09	-0.23	0.49	-0.11	0.37
Belarus	0.72	0	0	0	0	0	0	0	0	0.24	0.47	-0.02	0.04	0.27
Belgium	0.07	0.15	0.30	0.03	0	0.11	-0.05	-0.01	0.22	-0.03	0.15	0.12	-0.12	0.45
Bosnia and Herzegovina	0.07	-0.21	0.24	0.06	0.11	0.14	-0.06	0.02	0.02	0.05	0.47	0	0.02	0.35
Bulgaria	0.16	0.03	0.07	-0.04	0	-0.03	0.09	0.13	-0.01	0.35	0.24	-0.04	-0.05	0.38
Croatia	0.25	0.20	0.39	0.17	-0.01	0.03	-0.01	0.08	0.13	-0.83	0.05	0.21	0.22	0.17
Cyprus	0.17	-0.01	0.09	0.03	0.01	-0.01	0.05	0.08	0.44	0.13	-0.15	-0.234	-0.02	0.25
Czech Rep.	0.07	-0.02	0.03	-0.15	0	0.40	-0.08	0.05	0.03	0.06	0.34	0.28	0.03	0.39
Denmark	0.04	0.57	0.27	0.04	-0.43	0	0.05	0.06	0.1	0.21	-0.03	0.10	-0.24	0.38
Estonia	0.07	0.07	-0.36	-0.05	0	0.03	0	0.37	-0.02	0.61	0.05	-0.23	0	0.31
Finland	-0.06	0.30	-0.01	0.02	0	-0.10	0	0.20	0.07	0.42	-0.10	0.19	-0.34	0.44
France	0.04	0.04	0.07	0.08	-0.10	0.06	0.10	0.14	0.03	0.18	-0.10	0.35	-0.05	0.65
Germany	0.05	0.17	0.26	-0.04	0.08	-0.08	0.07	-0.01	0.07	0.27	-0.08	-0.15	0.61	0.50
Greece	0.09	0.0	0.03	0.09	0.27	0.37	-0.16	0.06	0.02	0.32	-0.04	0.02	-0.16	0.34
Hungary	0.04	0.14	0.07	0.02	-0.13	0.16	0.12	-0.08	0.19	0.41	0.03	-0.13	-0.12	0.30
Iceland	-0.44	0.32	0.09	0.26	0	0	0	0.32	0	0.25	-0.45	0.48	-0.49	0.37
Ireland	0.11	-0.24	-0.07	0.36	0.31	-0.07	0.13	-0.22	0.05	0.19	-0.12	0.51	-0.07	0.43
Italy	0.33	-0.08	0.10	-0.11	0.16	0.222	0.09	0.57	0.04	-0.90	0.13	0.02	0.72	0.28
Kosovo	0.27	-0.27	0.34	-0.11	0.21	0.16	-0.04	-0.12	0	0.63	0.17	-1.28	0.15	0.27
Latvia	0.01	-0.26	0.06	0.24	-0.41	0.90	0	0.06	0.55	0.04	-0.38	-0.07	-0.27	0.21
Lithuania	0.03	0.36	0	-0.09	-0.12	-0.76	-0.01	0.71	0.63	0.42	0.28	-0.77	-0.02	0.32
Luxembourg	0.18	-0.29	-0.15	-0.01	0.07	0.24	0.09	0.04	0.04	0.20	-0.34	0.51	0.29	0.34
Malta	0.01	0.15	0.07	0	0.02	0.08	-0.14	0.01	0.01	0.62	-0.15	0.17	-0.09	0.40
Moldova	-0.01	0.17	0	0.32	-0.04	0.01	0.16	-0.01	-0.01	0.37	-0.54	0.34	-0.08	0.39
Monaco	0.52	-0.38	-0.03	0.06	-0.68	0.28	0.18	-0.16	0	-0.22	0.26	0.81	0.03	0.16
Netherlands	0.07	0.20	0.11	-0.06	0.20	0.07	0.13	0.23	0.31	0.23	-2.41	0.27	1.66	0.23
Norway	-0.08	0.43	0.14	-0.10	0.12	-0.22	0.04	0.07	0	0.16	0.58	0.10	-0.41	0.45
Poland	0.189	0.06	-0.02	-0.12	0.03	0.10	0.04	-0.10	0.24	0.46	0.01	0.22	0.02	0.53
Portugal	0.07	-0.24	0.20	-0.14	0.10	-0.02	0.02	0.67	0.06	0.09	0.32	0.04	-0.08	0.43
Romania	0.34	-0.06	0.11	0.12	-0.06	0.10	-0.11	0.22	0.12	0.29	-0.21	-0.24	-0.03	0.50
Russia	-0.03	0.11	0.07	0.03	0.02	-0.18	0.08	0.08	0.06	0.08	0.05	0.29	0.19	0.42
San Marino	0.18	0.11	0.06	0.11	-0.25	-0.25	0.34	0.18	-0.40	0.26	0.50	-0.19	-0.19	0.24
Serbia	0.35	0.14	-0.18	0.04	-0.16	0.01	-0.02	0.06	0.34	0.30	-0.16	0.18	-0.19	0.38
Slovenia	0.20	0.06	-0.20	0	-0.02	0.06	0.14	-0.11	0.25	0.33	0.22	0.02	-0.14	0.28
Spain	-0.05	0.07	0.14	0.27	0.11	-0.01	-0.20	0	0.30	0.51	-0.30	-0.43	0.14	0.49
Sweden	0.38	0.01	-0.13	0.18	0.21	0.12	-0.09	0.03	0.02	0.30	0.02	0.02	0	0.39
Switzerland	0.20	-0.10	0.10	0.11	0	-0.04	0.11	0.11	0.10	0.08	0.13	0.35	-0.08	0.47
Turkey	-0.15	0.09	0.14	-0.15	-0.10	0.11	0.16	0	0.15	0.12	0.66	0.05	0.02	0.95
Ukraine	0.09	-0.13	-0.07	0.11	0.06	0.34	0.09	0.04	0.28	0.31	-0.01	-0.18	0.12	0.39
UK	0.30	-0.07	0.34	0.32	-0.02	-0.21	-0.01	-0.05	-0.10	0.03	0.26	-0.14	0.04	0.41

Supplementary Table 1. Parameters estimated for the independent models. a_1: School closing, a_2: workplace closing, a_3: cancel public events, a_4: restrictions on gatherings, a_5: close public transport, a_6: stay at home requirements, a_7: restrictions on internal movement, a_8: income support, a_9: debt/contract relief, a_{10}: public information campaigns, a_{11}: testing policy, a_{12}: contact tracing, a_{13}: facial coverings.

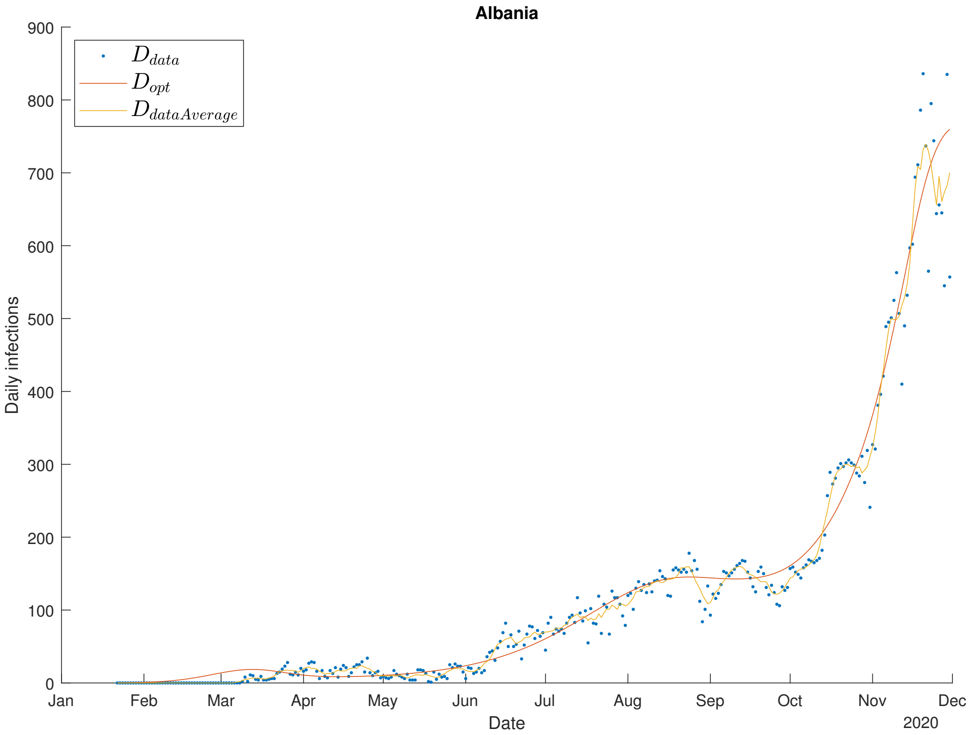

(a) Fit based on $\beta_o pt$ over the training time period. Blue points represent daily infections, the yellow line the seven-day moving average of daily infections, and the red line is the fit obtained by substituting the $\beta_{opt}(t)$ into the SEIR model.

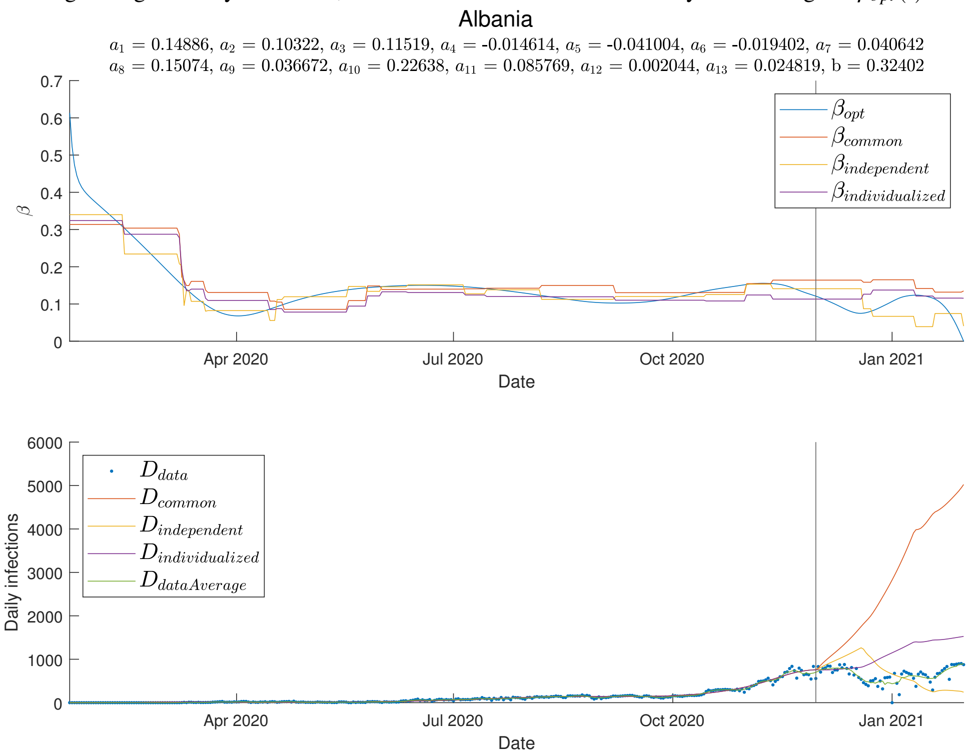

(b) Estimation results. Top panel: fitting a function of restrictions to the signal β_{opt}. Bottom panel: Ability of the model to predict daily infections. The black vertical line indicates the beginning of the validation period. As the number of daily infections is highly variable, a seven day moving average is also presented

Supplementary Figure 43. Prediction results - Albania.

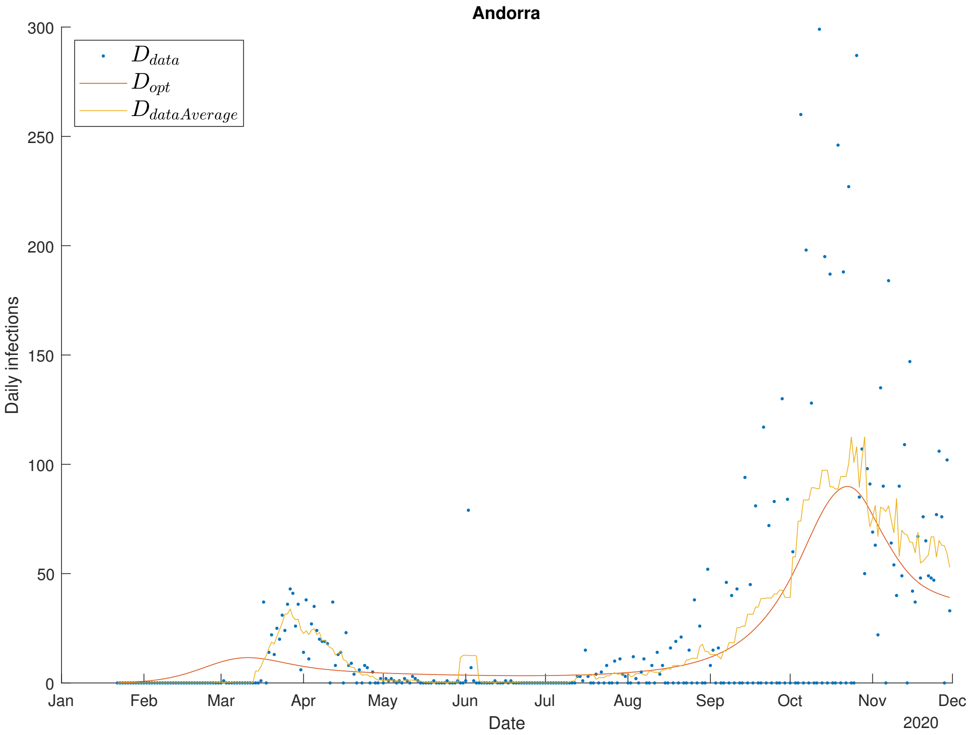

(a) Fit based on $\beta_o pt$ over the training time period. Blue points represent daily infections, the yellow line the seven-day moving average of daily infections, and the red line is the fit obtained by substituting the $\beta_{opt}(t)$ into the SEIR model.

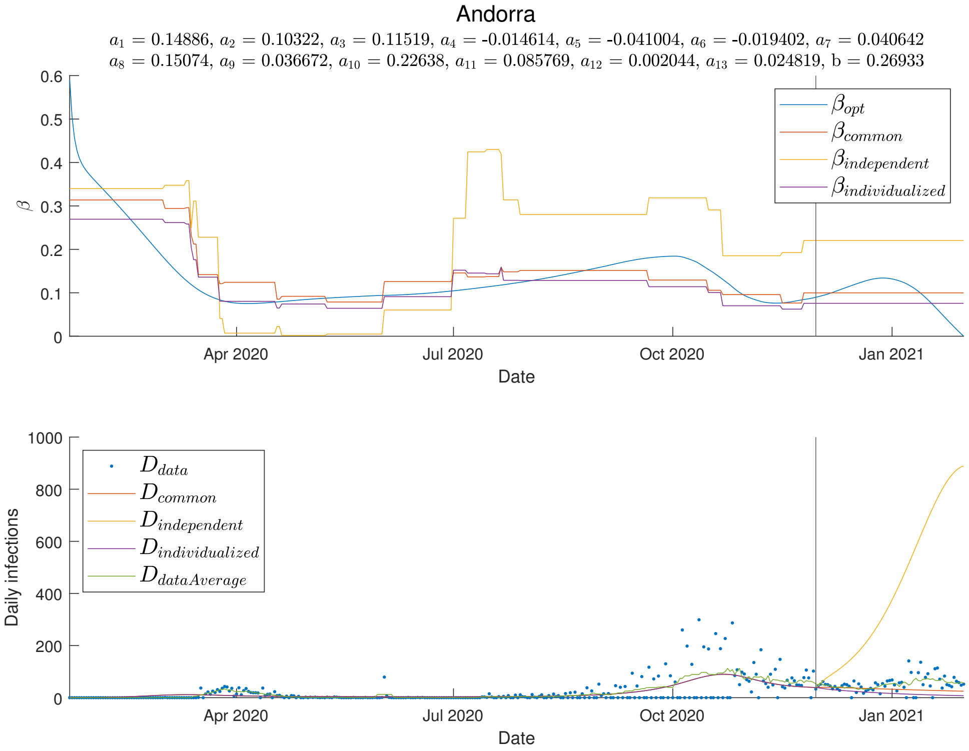

(b) Estimation results. Top panel: fitting a function of restrictions to the signal β_{opt}. Bottom panel: Ability of the model to predict daily infections. The black vertical line indicates the beginning of the validation period. As the number of daily infections is highly variable, a seven day moving average is also presented

Supplementary Figure 44. Prediction results - Andorra.

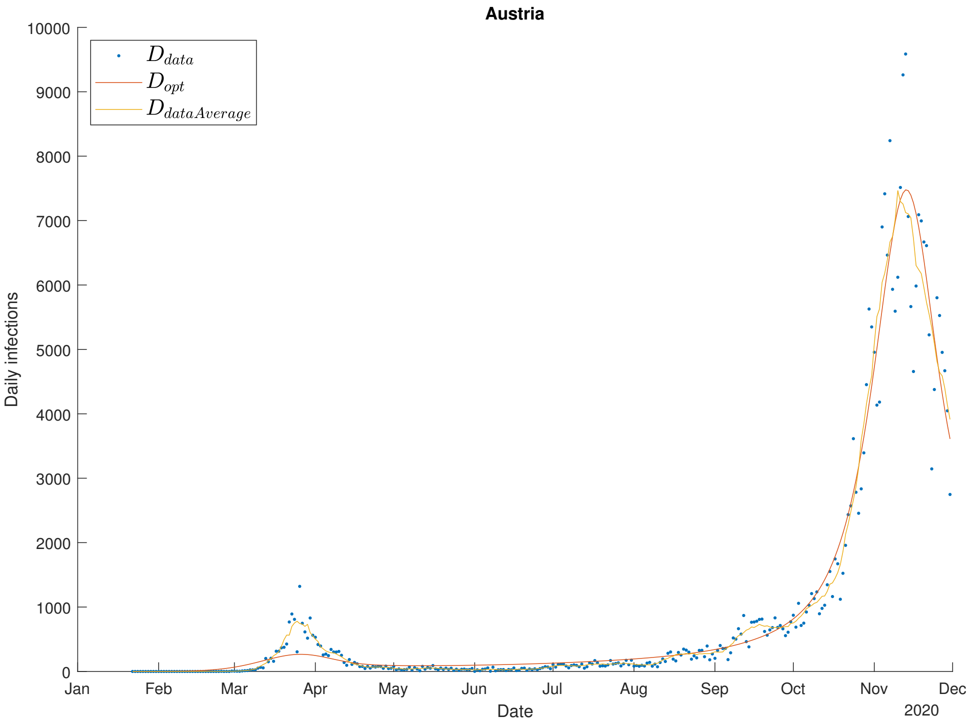

(a) Fit based on $\beta_o pt$ over the training time period. Blue points represent daily infections, the yellow line the seven-day moving average of daily infections, and the red line is the fit obtained by substituting the $\beta_{opt}(t)$ into the SEIR model.

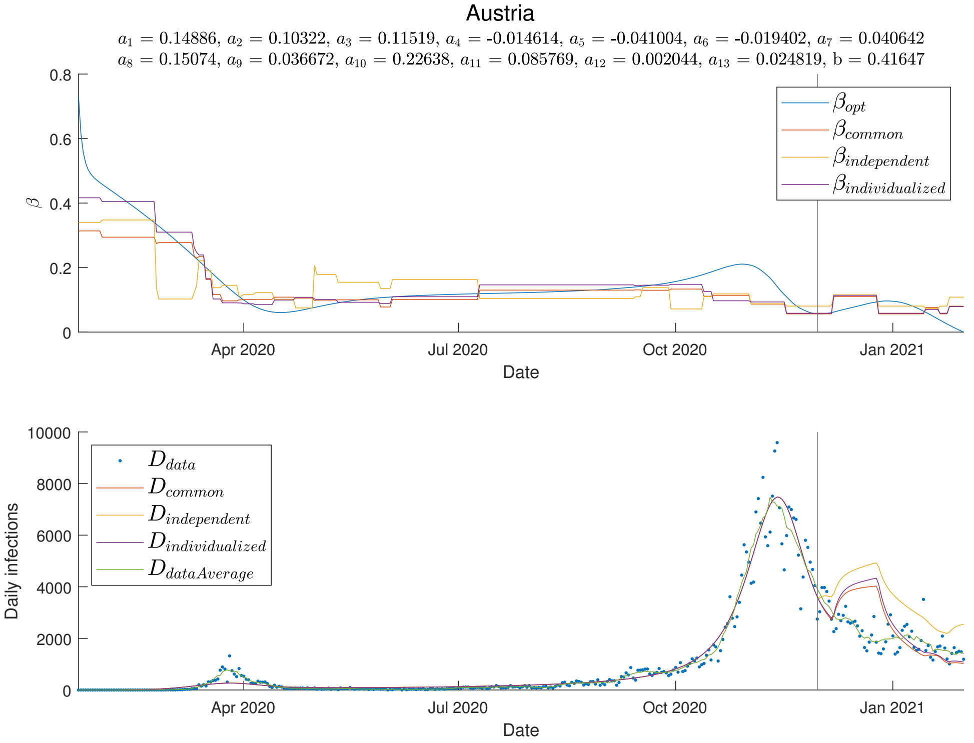

(b) Estimation results. Top panel: fitting a function of restrictions to the signal β_{opt}. Bottom panel: Ability of the model to predict daily infections. The black vertical line indicates the beginning of the validation period. As the number of daily infections is highly variable, a seven day moving average is also presented

Supplementary Figure 45. Prediction results - Austria.

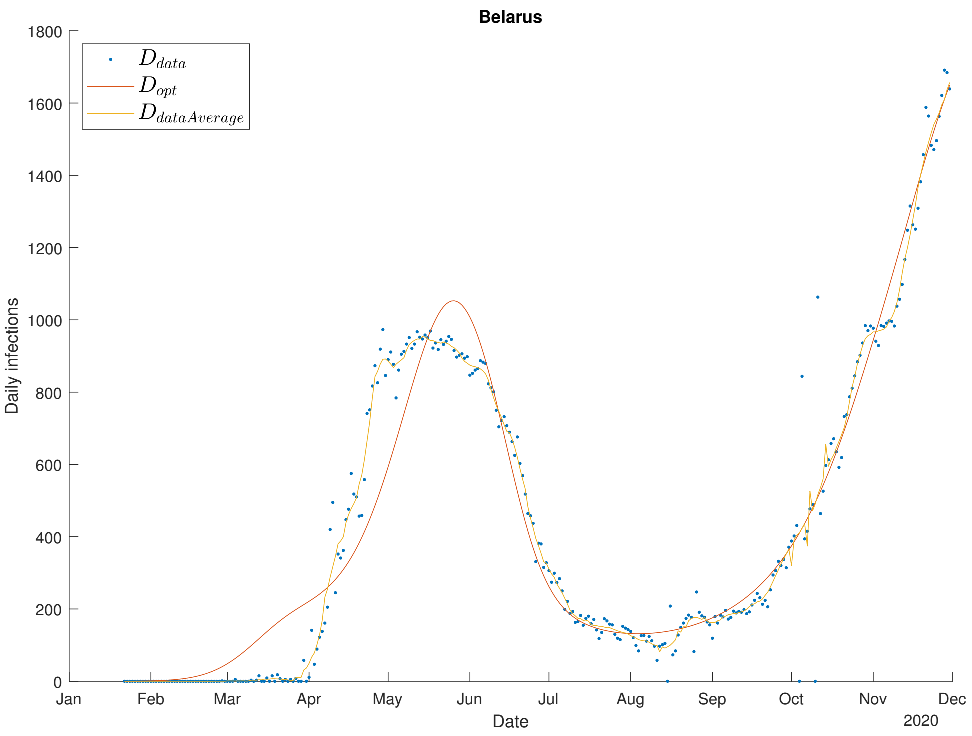

(a) Fit based on $\beta_o pt$ over the training time period. Blue points represent daily infections, the yellow line the seven-day moving average of daily infections, and the red line is the fit obtained by substituting the $\beta_{opt}(t)$ into the SEIR model.

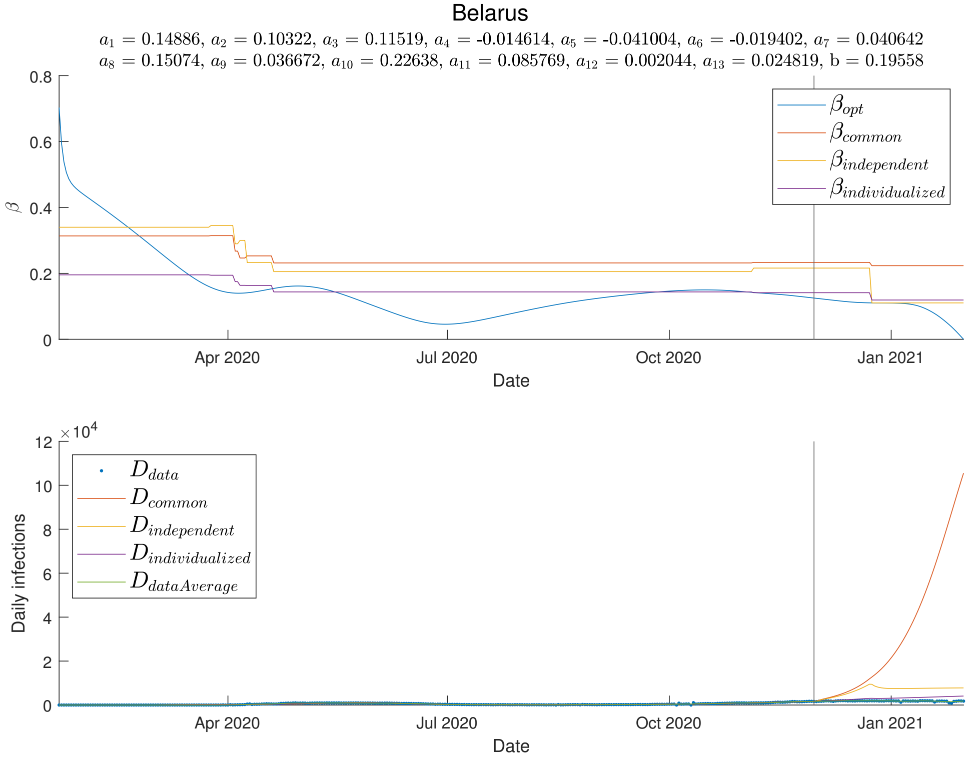

(b) Estimation results. Top panel: fitting a function of restrictions to the signal β_{opt}. Bottom panel: Ability of the model to predict daily infections. The black vertical line indicates the beginning of the validation period. As the number of daily infections is highly variable, a seven day moving average is also presented

Supplementary Figure 46. Prediction results - Belarus.

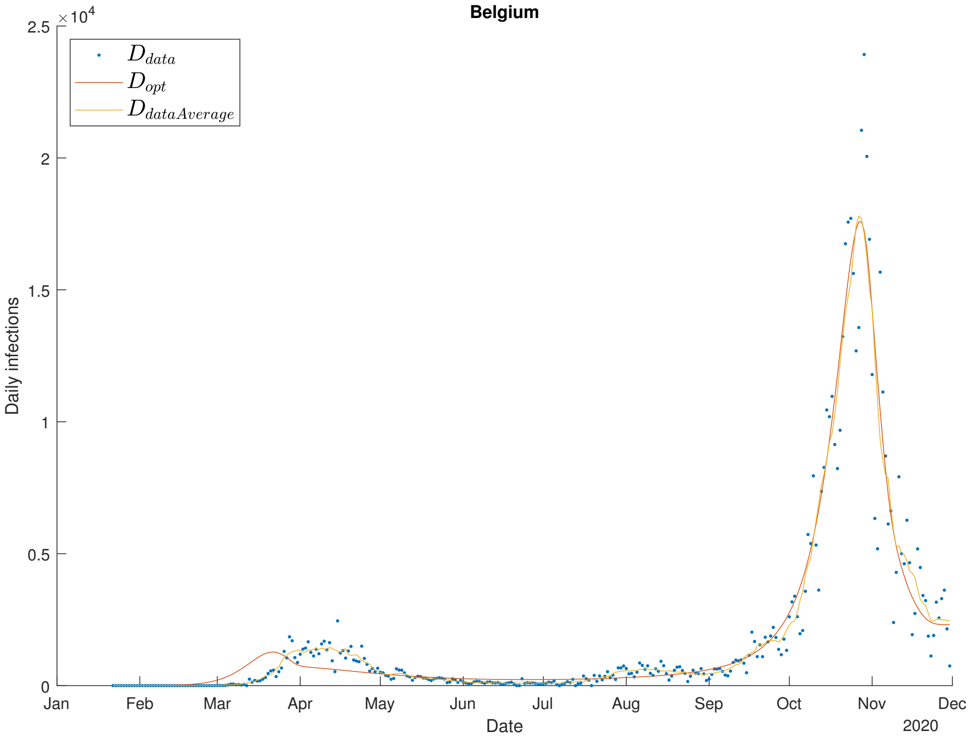

(a) Fit based on $\beta_o pt$ over the training time period. Blue points represent daily infections, the yellow line the seven-day moving average of daily infections, and the red line is the fit obtained by substituting the $\beta_{opt}(t)$ into the SEIR model.

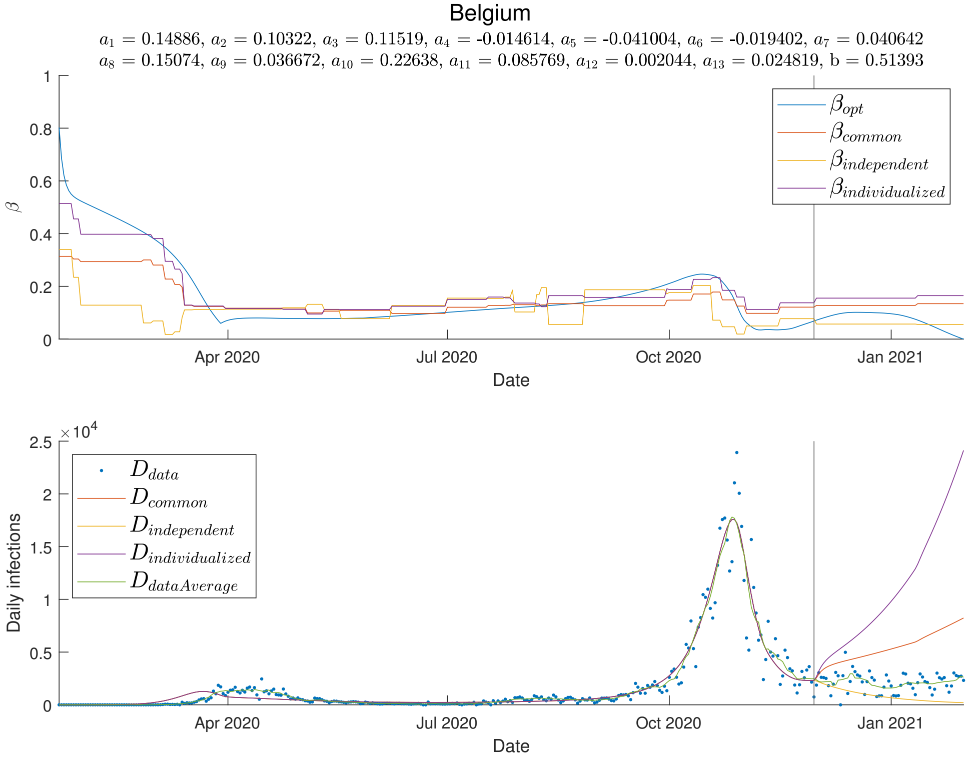

(b) Estimation results. Top panel: fitting a function of restrictions to the signal β_{opt}. Bottom panel: Ability of the model to predict daily infections. The black vertical line indicates the beginning of the validation period. As the number of daily infections is highly variable, a seven day moving average is also presented

Supplementary Figure 47. Prediction results - Belgium.

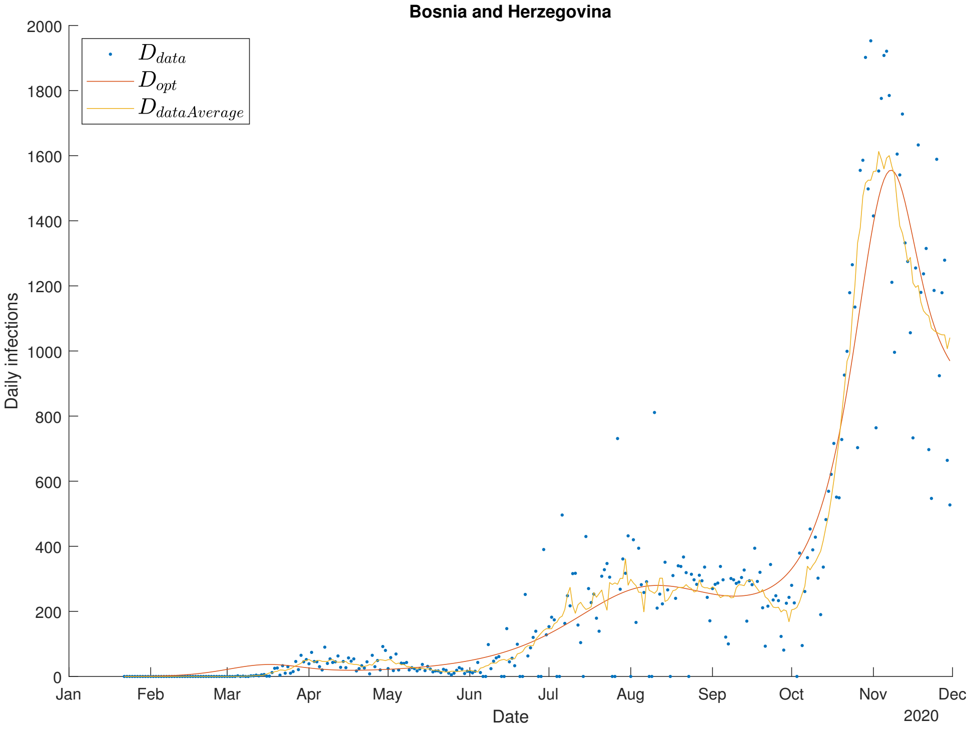

(a) Fit based on $\beta_o pt$ over the training time period. Blue points represent daily infections, the yellow line the seven-day moving average of daily infections, and the red line is the fit obtained by substituting the $\beta_{opt}(t)$ into the SEIR model.

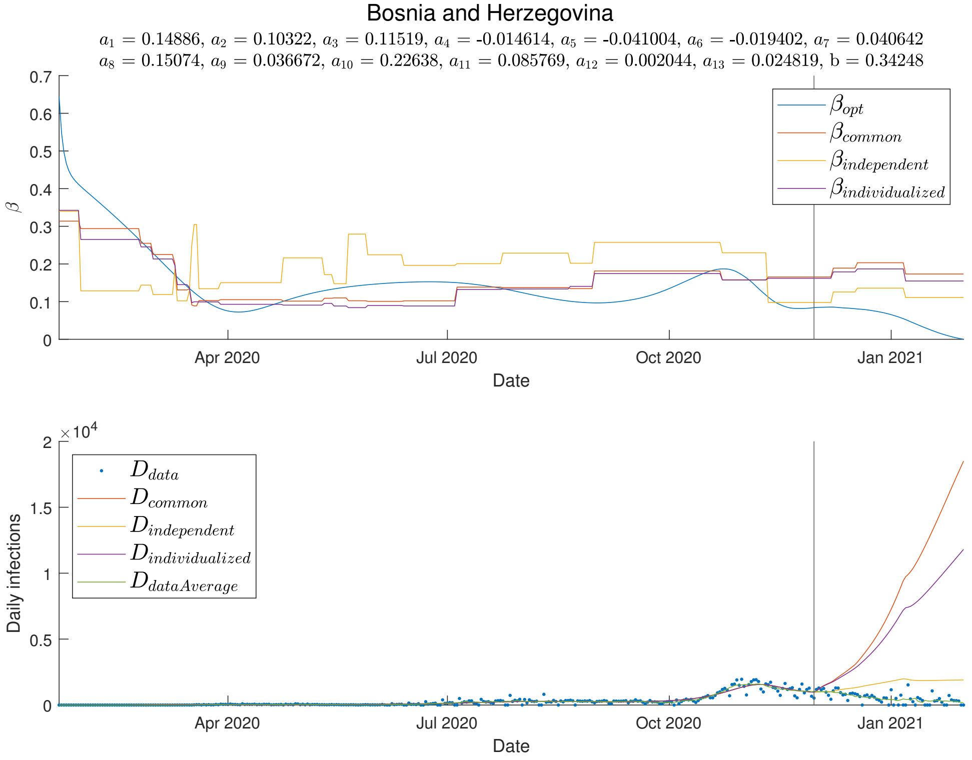

(b) Estimation results. Top panel: fitting a function of restrictions to the signal β_{opt}. Bottom panel: Ability of the model to predict daily infections. The black vertical line indicates the beginning of the validation period. As the number of daily infections is highly variable, a seven day moving average is also presented

Supplementary Figure 48. Prediction results - Bosnia and Herzegovina.

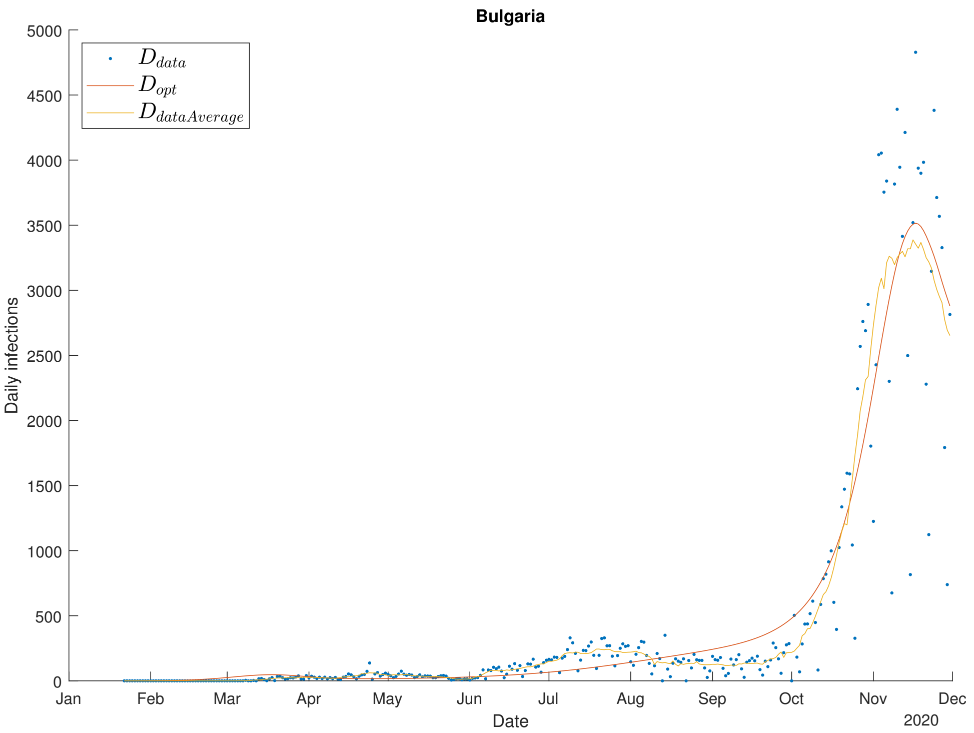

(a) Fit based on $\beta_o pt$ over the training time period. Blue points represent daily infections, the yellow line the seven-day moving average of daily infections, and the red line is the fit obtained by substituting the $\beta_{opt}(t)$ into the SEIR model.

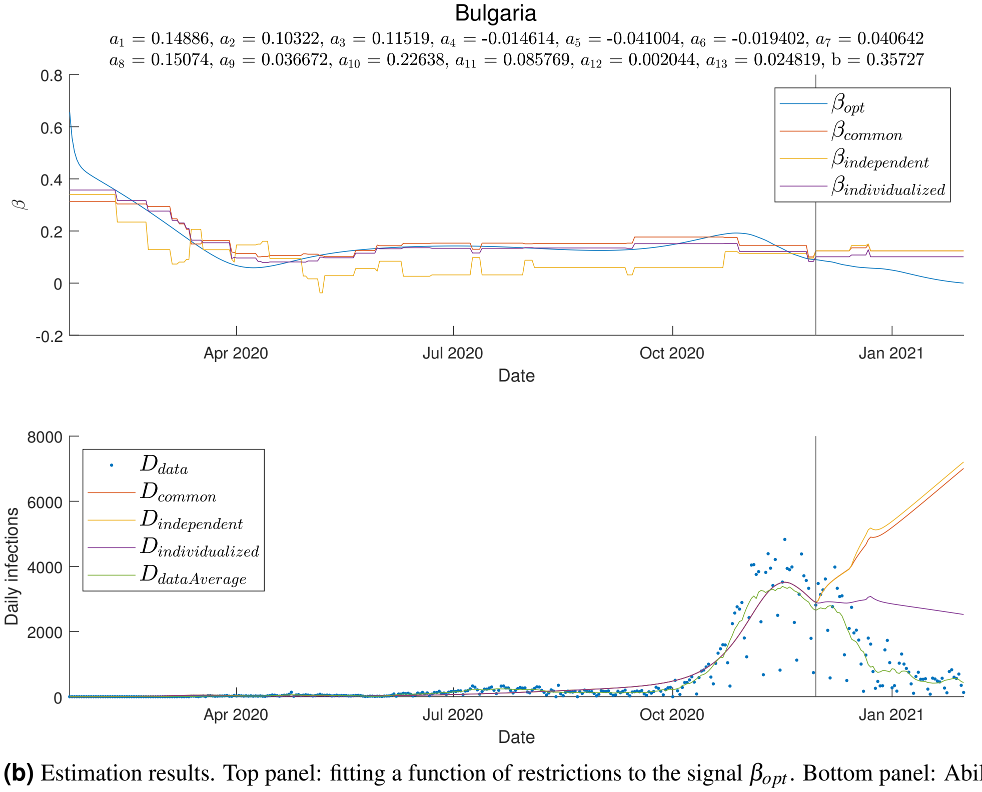

(b) Estimation results. Top panel: fitting a function of restrictions to the signal β_{opt}. Bottom panel: Ability of the model to predict daily infections. The black vertical line indicates the beginning of the validation period. As the number of daily infections is highly variable, a seven day moving average is also presented

Supplementary Figure 49. Prediction results - Bulgaria.

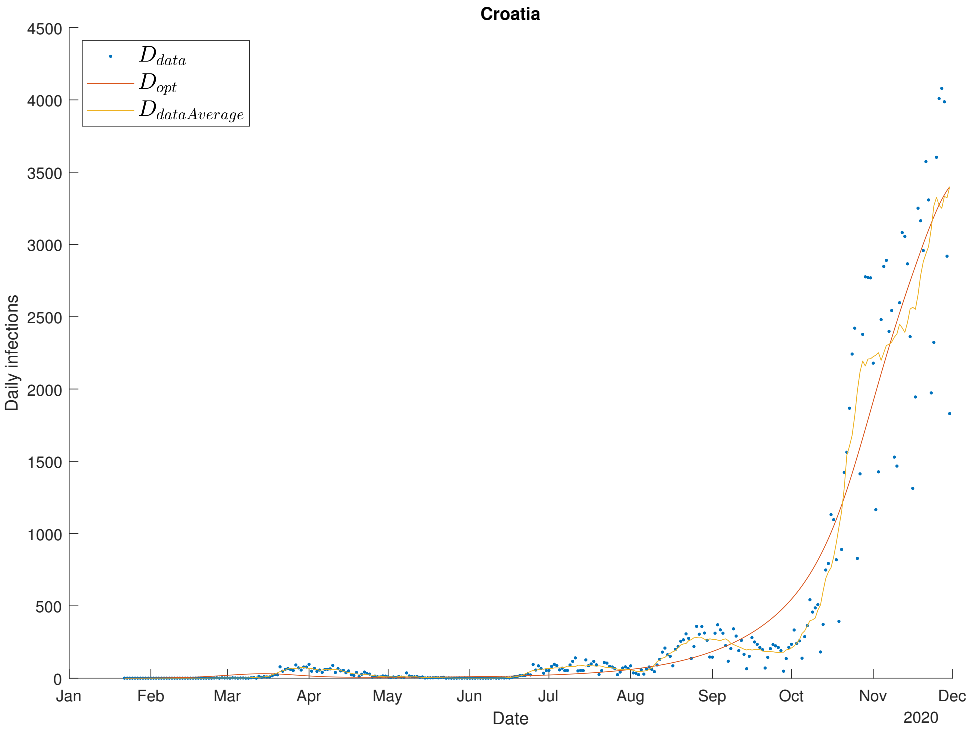

(a) Fit based on $\beta_o pt$ over the training time period. Blue points represent daily infections, the yellow line the seven-day moving average of daily infections, and the red line is the fit obtained by substituting the $\beta_{opt}(t)$ into the SEIR model.

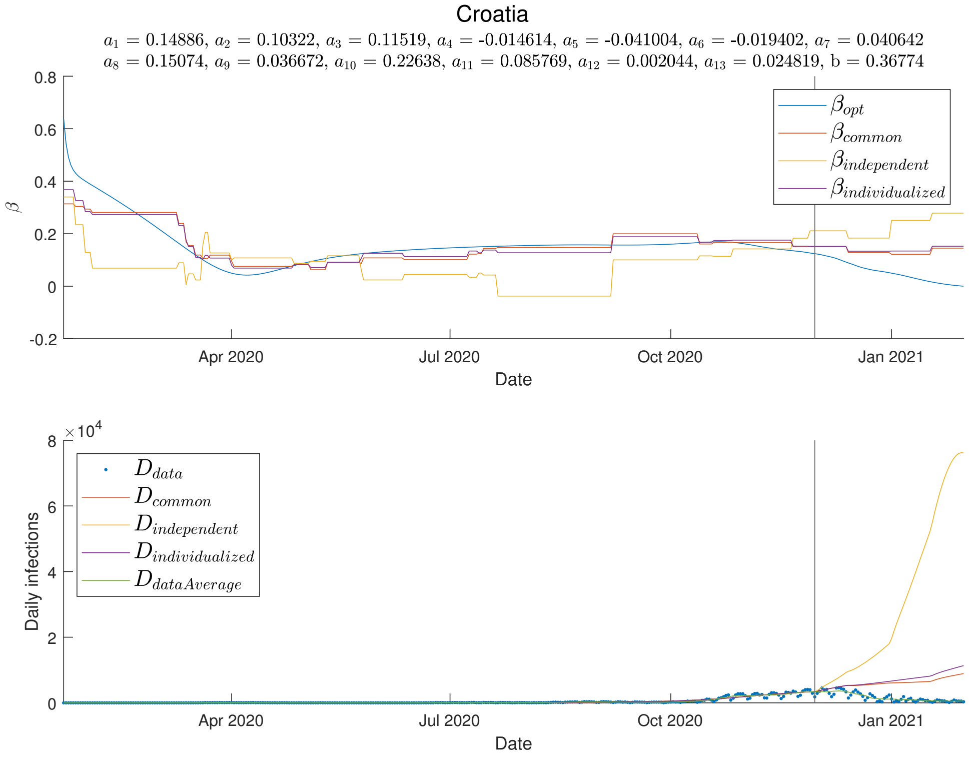

(b) Estimation results. Top panel: fitting a function of restrictions to the signal β_{opt}. Bottom panel: Ability of the model to predict daily infections. The black vertical line indicates the beginning of the validation period. As the number of daily infections is highly variable, a seven day moving average is also presented

Supplementary Figure 50. Prediction results - Croatia.

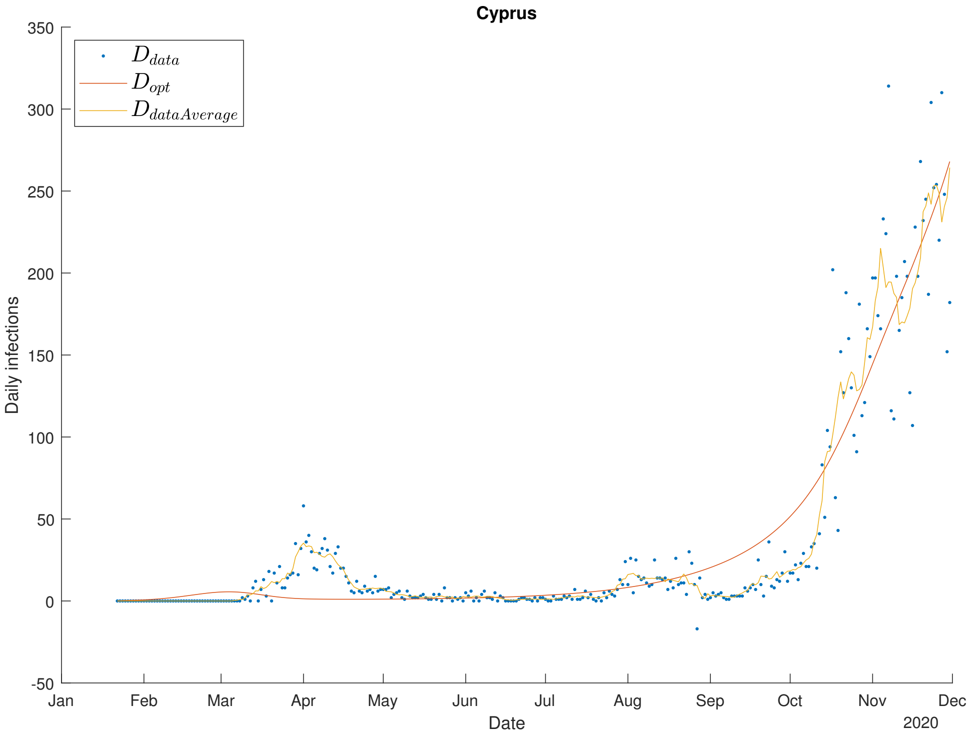

(a) Fit based on $\beta_o pt$ over the training time period. Blue points represent daily infections, the yellow line the seven-day moving average of daily infections, and the red line is the fit obtained by substituting the $\beta_{opt}(t)$ into the SEIR model.

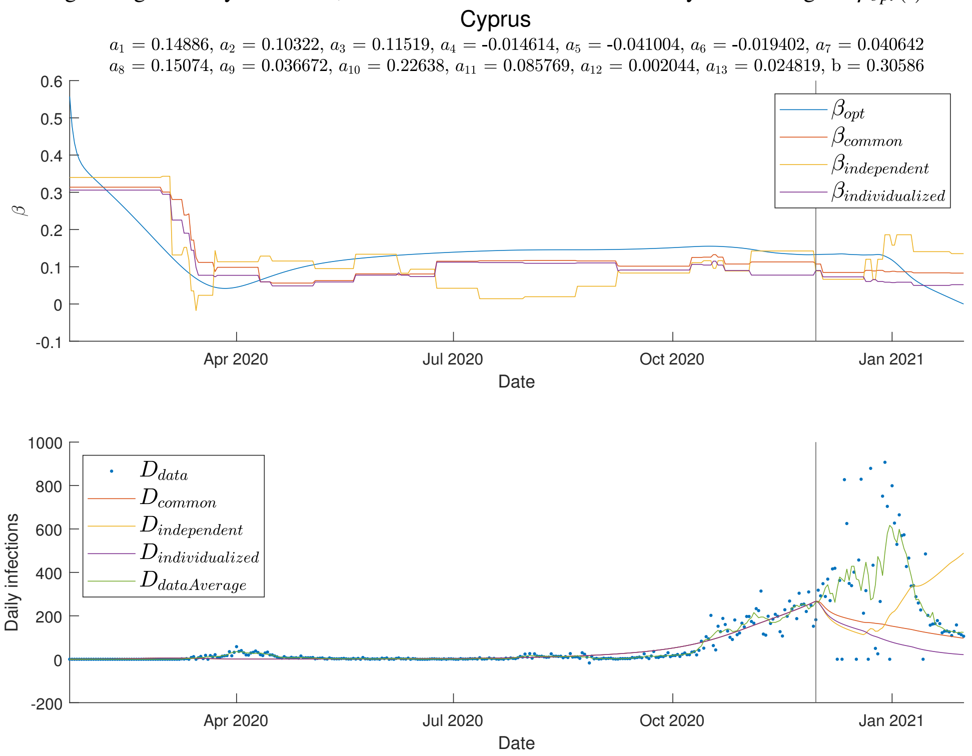

(b) Estimation results. Top panel: fitting a function of restrictions to the signal β_{opt}. Bottom panel: Ability of the model to predict daily infections. The black vertical line indicates the beginning of the validation period. As the number of daily infections is highly variable, a seven day moving average is also presented

Supplementary Figure 51. Prediction results - Cyprus.

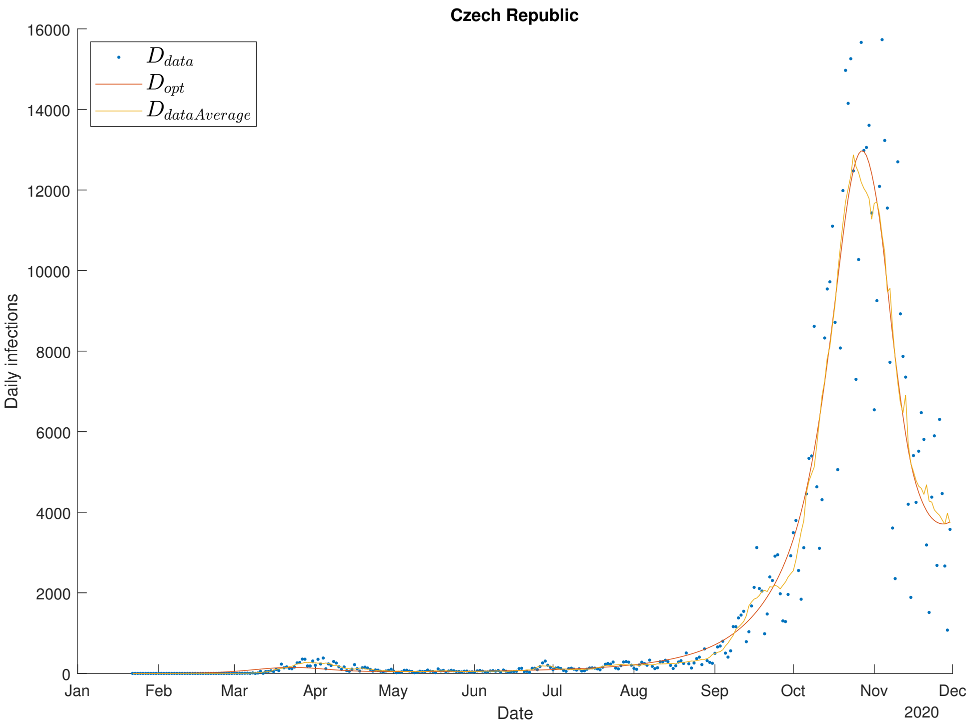

(a) Fit based on $\beta_o pt$ over the training time period. Blue points represent daily infections, the yellow line the seven-day moving average of daily infections, and the red line is the fit obtained by substituting the $\beta_{opt}(t)$ into the SEIR model.

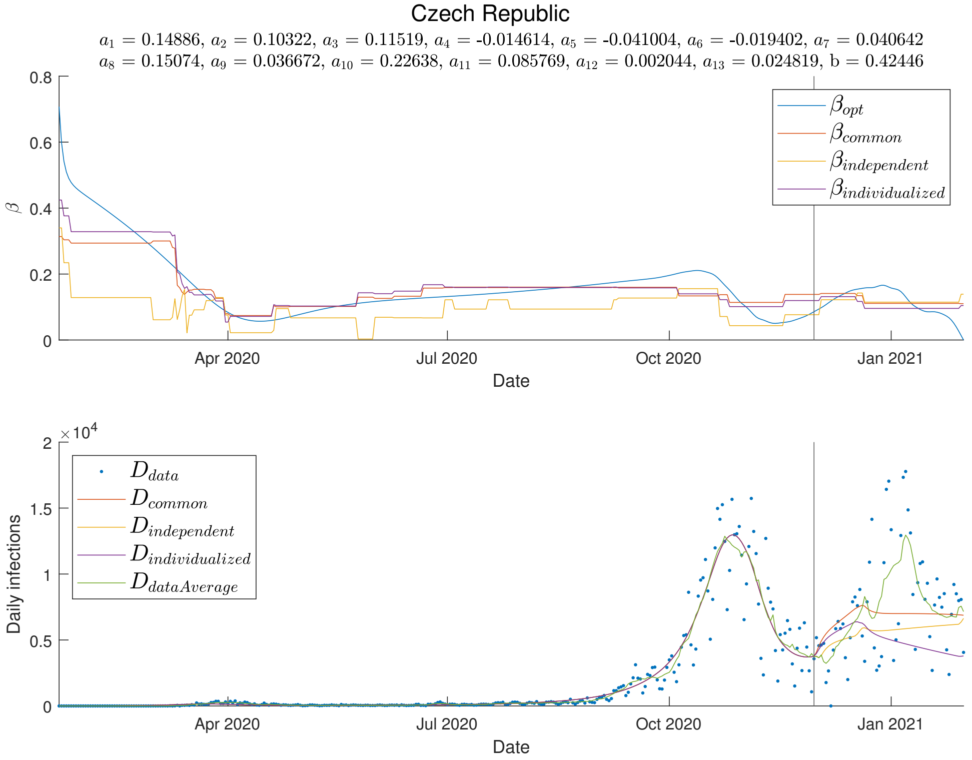

(b) Estimation results. Top panel: fitting a function of restrictions to the signal β_{opt}. Bottom panel: Ability of the model to predict daily infections. The black vertical line indicates the beginning of the validation period. As the number of daily infections is highly variable, a seven day moving average is also presented

Supplementary Figure 52. Prediction results - Czech Republic.

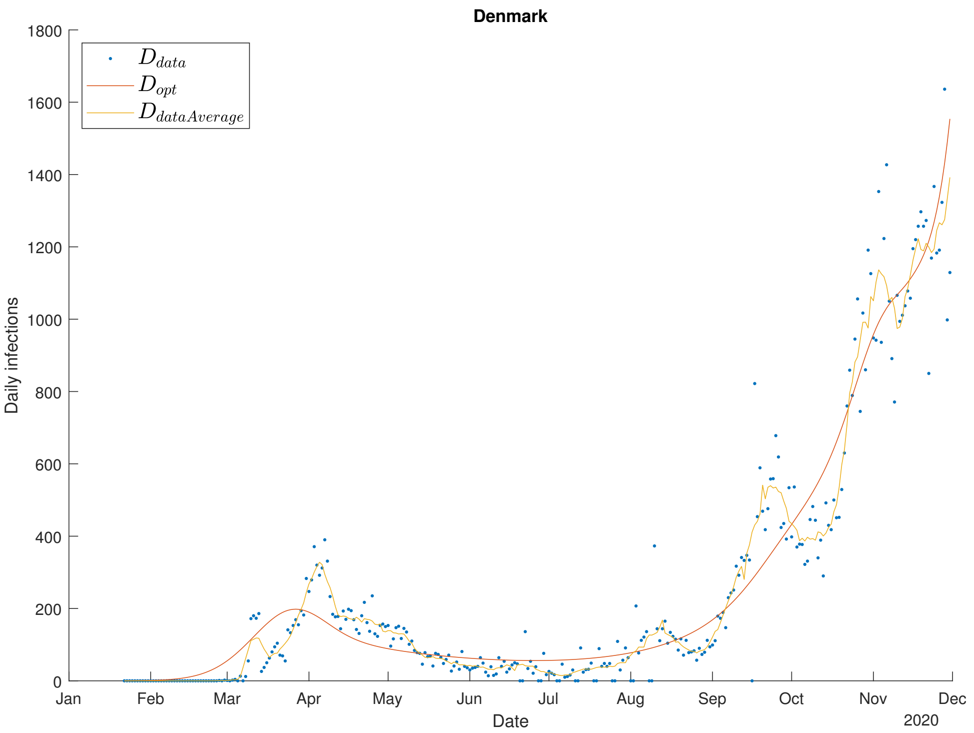

(a) Fit based on $\beta_o pt$ over the training time period. Blue points represent daily infections, the yellow line the seven-day moving average of daily infections, and the red line is the fit obtained by substituting the $\beta_{opt}(t)$ into the SEIR model.

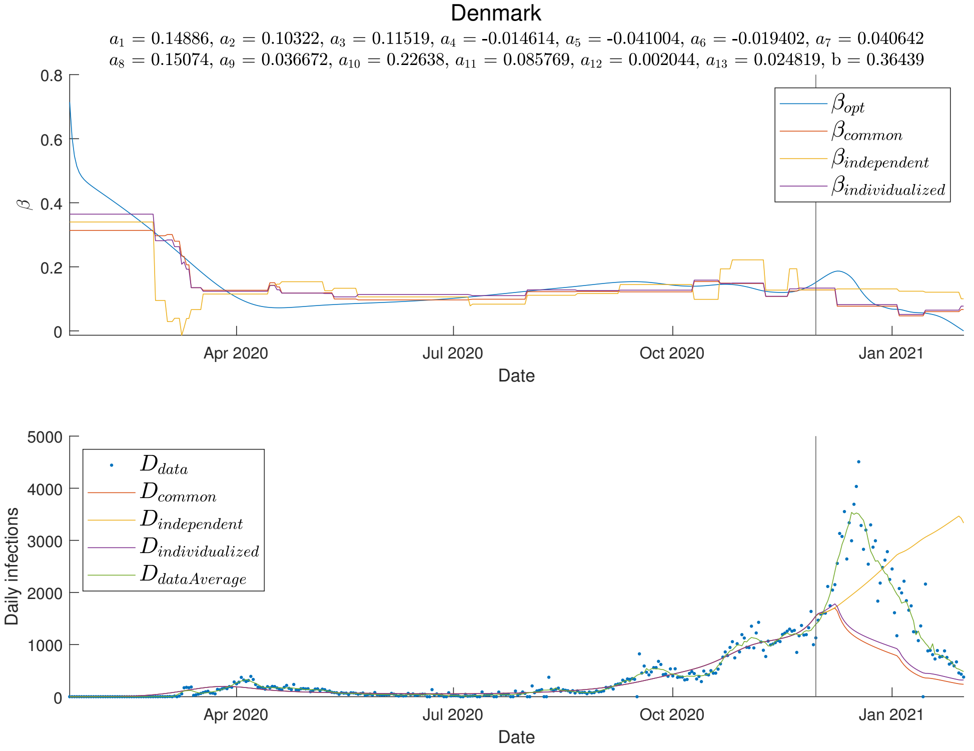

(b) Estimation results. Top panel: fitting a function of restrictions to the signal β_{opt}. Bottom panel: Ability of the model to predict daily infections. The black vertical line indicates the beginning of the validation period. As the number of daily infections is highly variable, a seven day moving average is also presented

Supplementary Figure 53. Prediction results - Denmark.

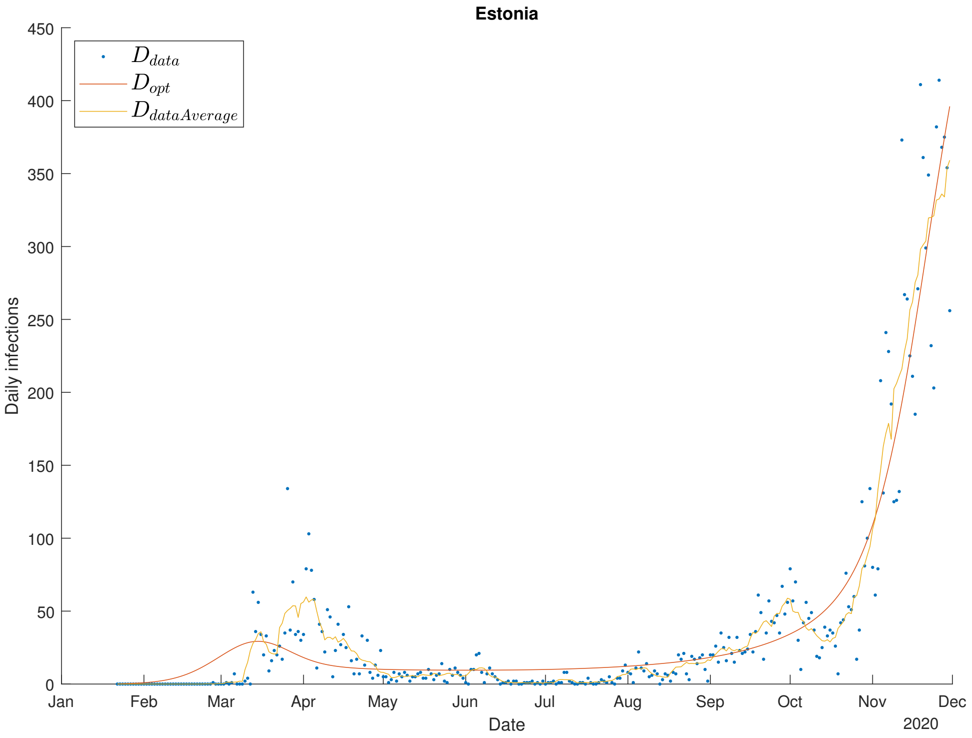

(a) Fit based on $\beta_o pt$ over the training time period. Blue points represent daily infections, the yellow line the seven-day moving average of daily infections, and the red line is the fit obtained by substituting the $\beta_{opt}(t)$ into the SEIR model.

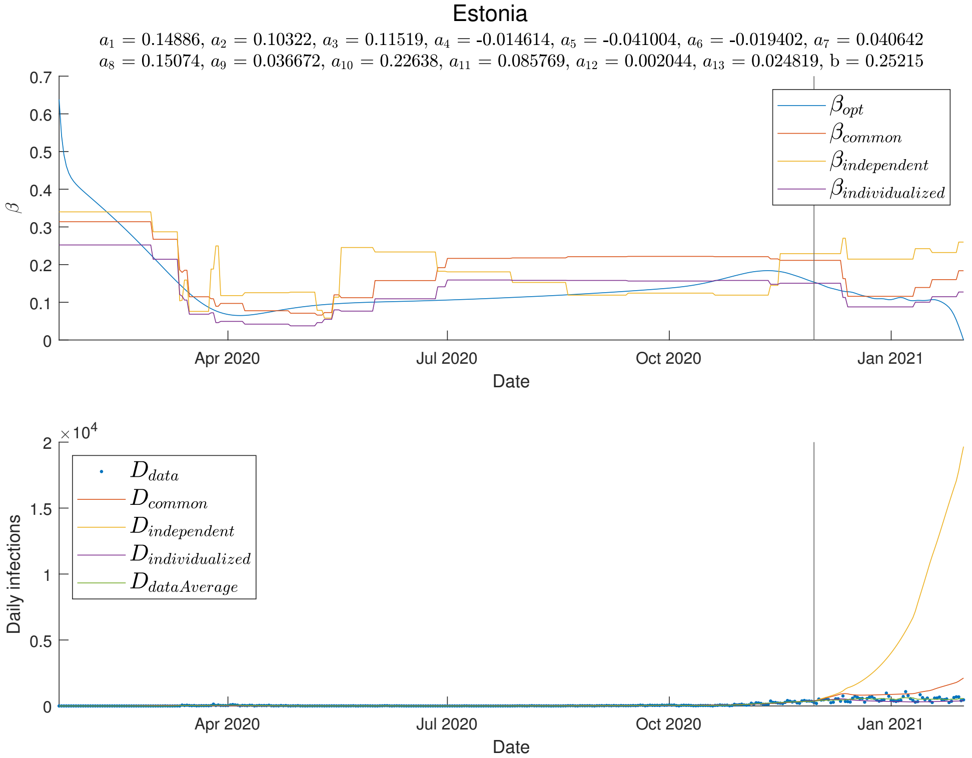

(b) Estimation results. Top panel: fitting a function of restrictions to the signal β_{opt}. Bottom panel: Ability of the model to predict daily infections. The black vertical line indicates the beginning of the validation period. As the number of daily infections is highly variable, a seven day moving average is also presented

Supplementary Figure 54. Prediction results - Estonia.

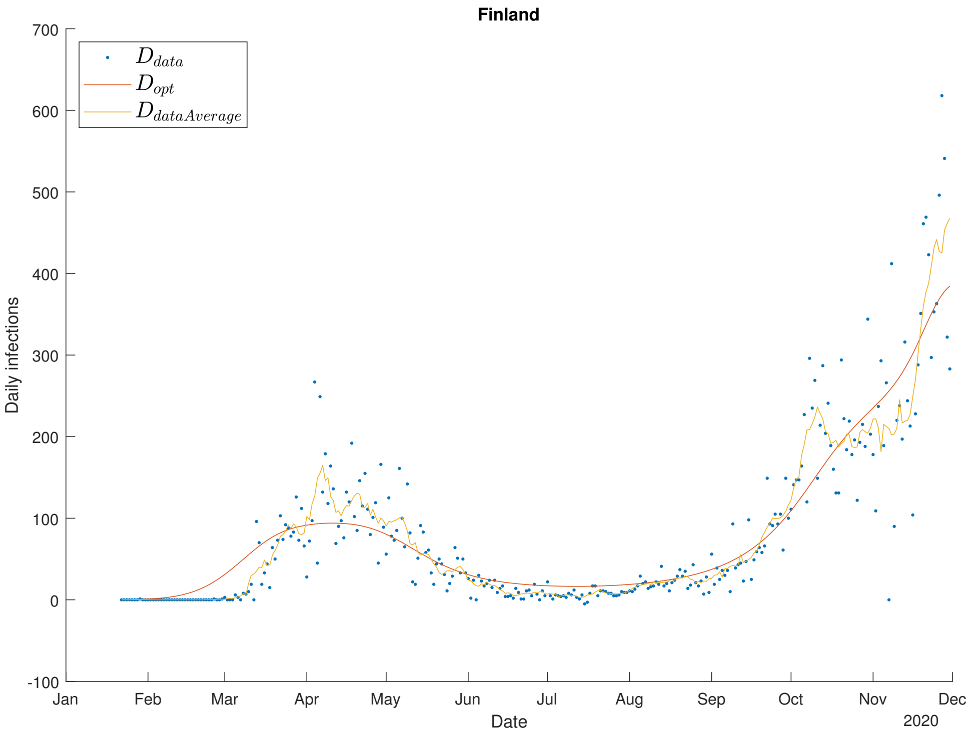

(a) Fit based on $\beta_o pt$ over the training time period. Blue points represent daily infections, the yellow line the seven-day moving average of daily infections, and the red line is the fit obtained by substituting the $\beta_{opt}(t)$ into the SEIR model.

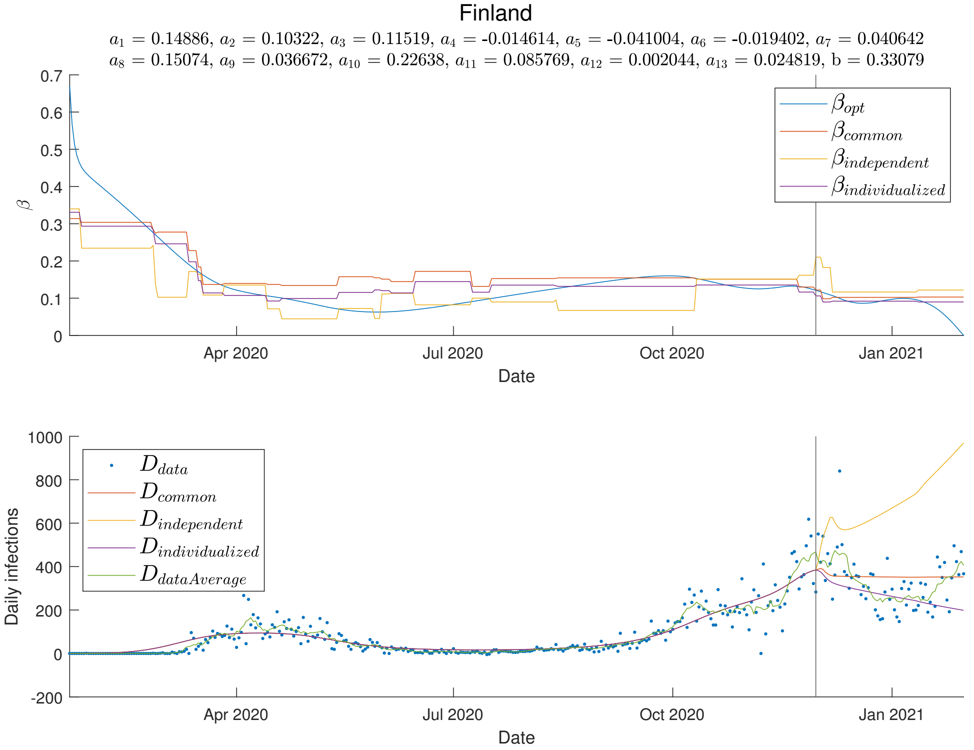

(b) Estimation results. Top panel: fitting a function of restrictions to the signal β_{opt}. Bottom panel: Ability of the model to predict daily infections. The black vertical line indicates the beginning of the validation period. As the number of daily infections is highly variable, a seven day moving average is also presented

Supplementary Figure 55. Prediction results - Finland.

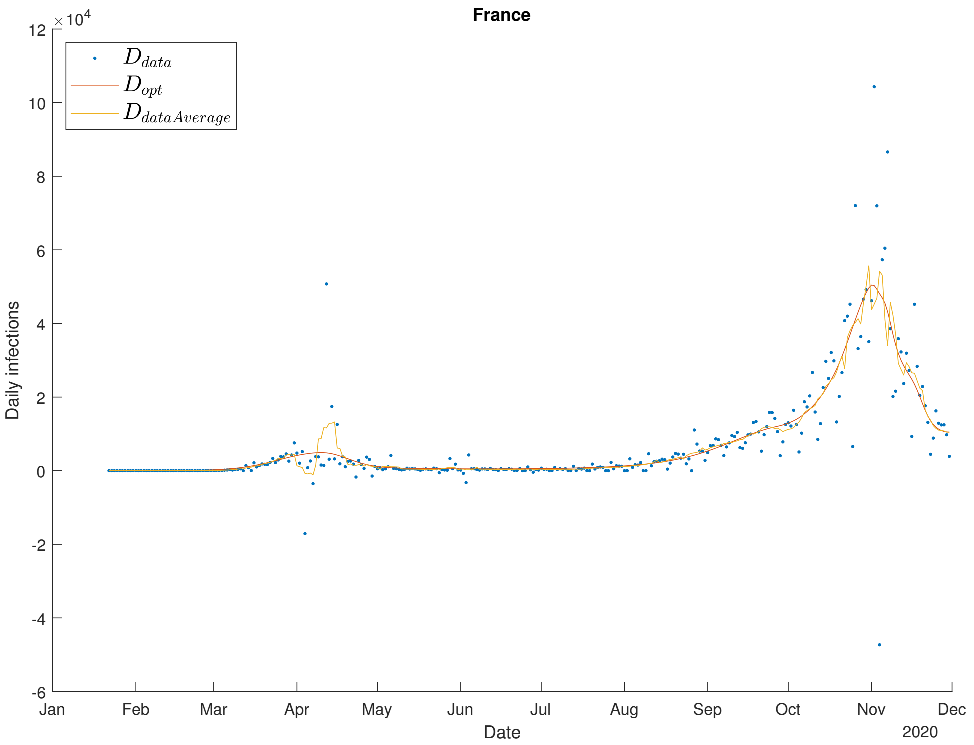

(a) Fit based on $\beta_o pt$ over the training time period. Blue points represent daily infections, the yellow line the seven-day moving average of daily infections, and the red line is the fit obtained by substituting the $\beta_{opt}(t)$ into the SEIR model.

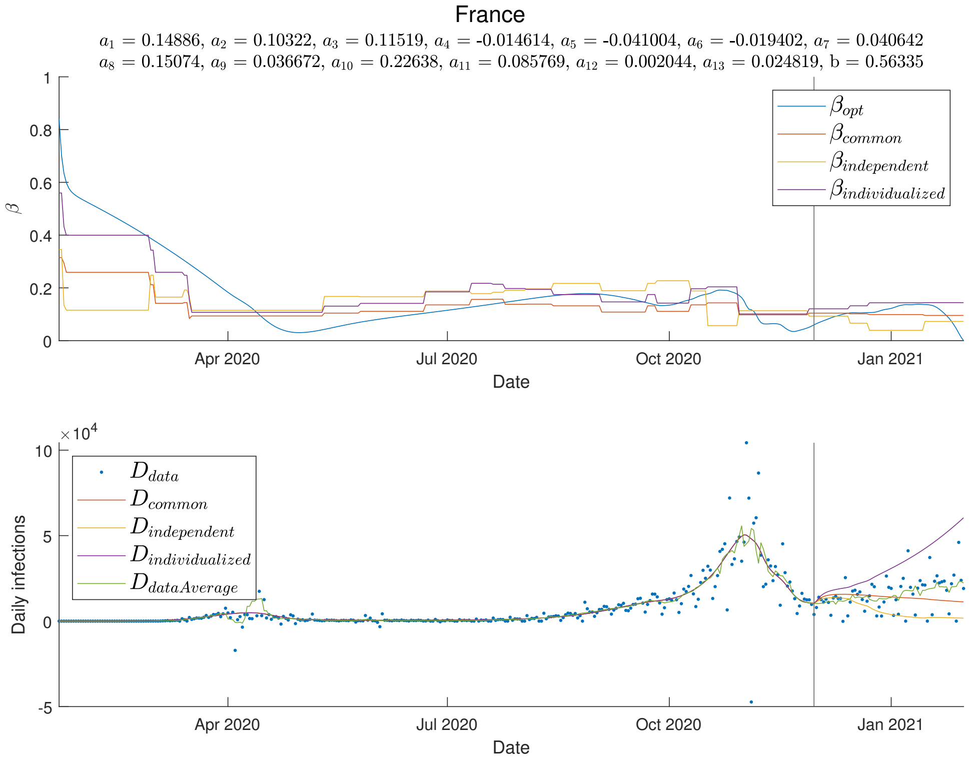

(b) Estimation results. Top panel: fitting a function of restrictions to the signal β_{opt}. Bottom panel: Ability of the model to predict daily infections. The black vertical line indicates the beginning of the validation period. As the number of daily infections is highly variable, a seven day moving average is also presented

Supplementary Figure 56. Prediction results - France.

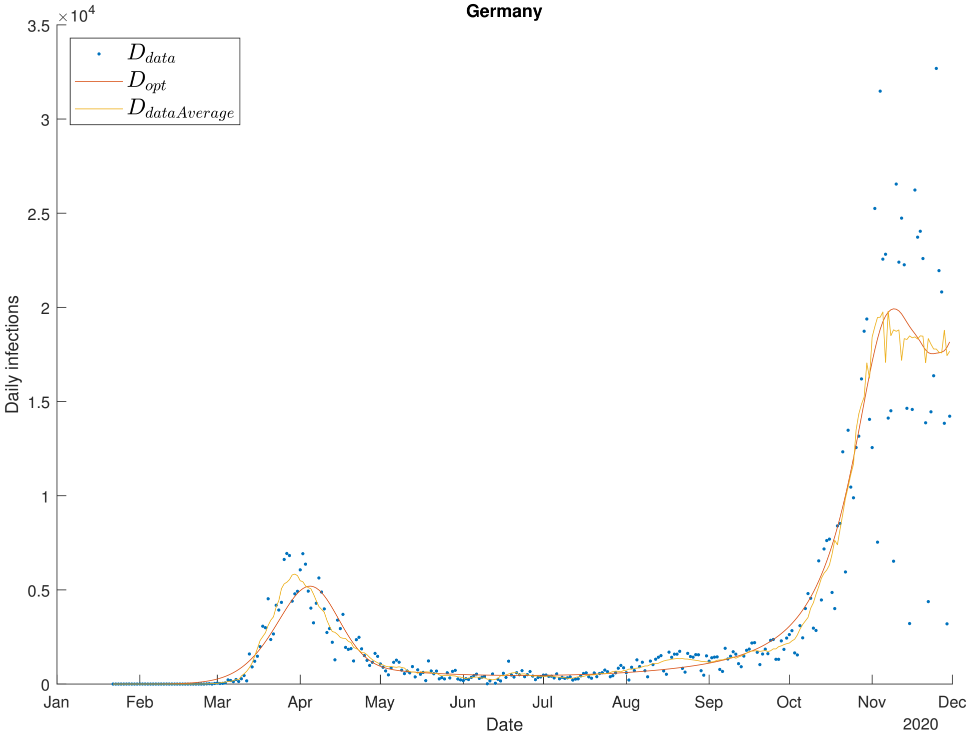

(a) Fit based on β_opt over the training time period. Blue points represent daily infections, the yellow line the seven-day moving average of daily infections, and the red line is the fit obtained by substituting the $\beta_{opt}(t)$ into the SEIR model.

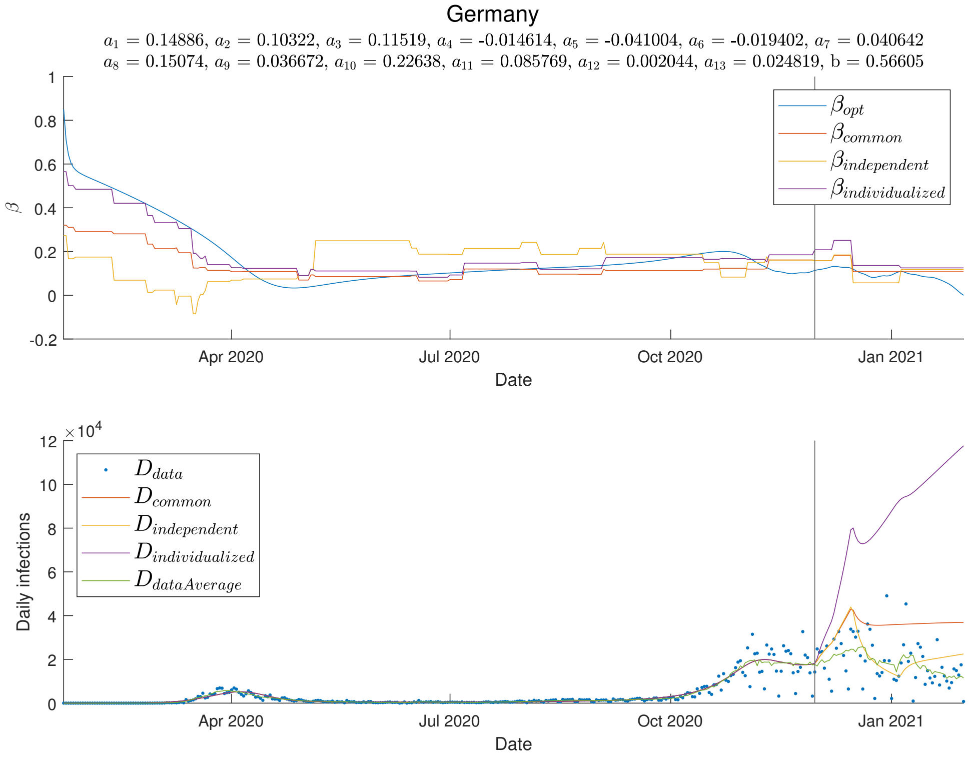

(b) Estimation results. Top panel: fitting a function of restrictions to the signal β_{opt}. Bottom panel: Ability of the model to predict daily infections. The black vertical line indicates the beginning of the validation period. As the number of daily infections is highly variable, a seven day moving average is also presented

Supplementary Figure 57. Prediction results - Germany.

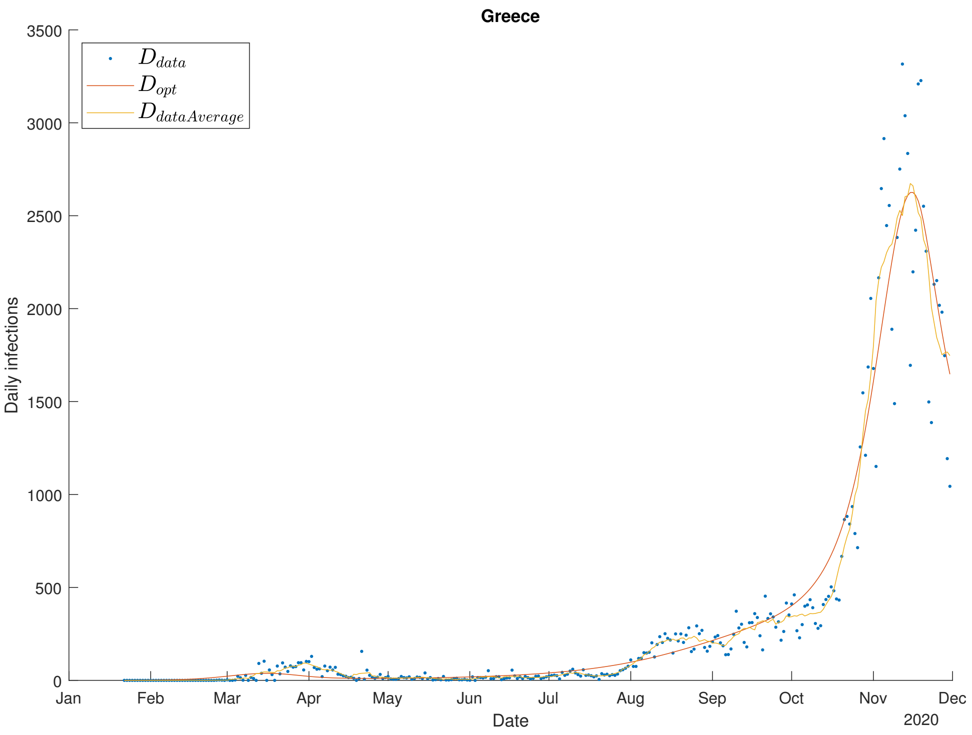

(a) Fit based on $\beta_o pt$ over the training time period. Blue points represent daily infections, the yellow line the seven-day moving average of daily infections, and the red line is the fit obtained by substituting the $\beta_{opt}(t)$ into the SEIR model.

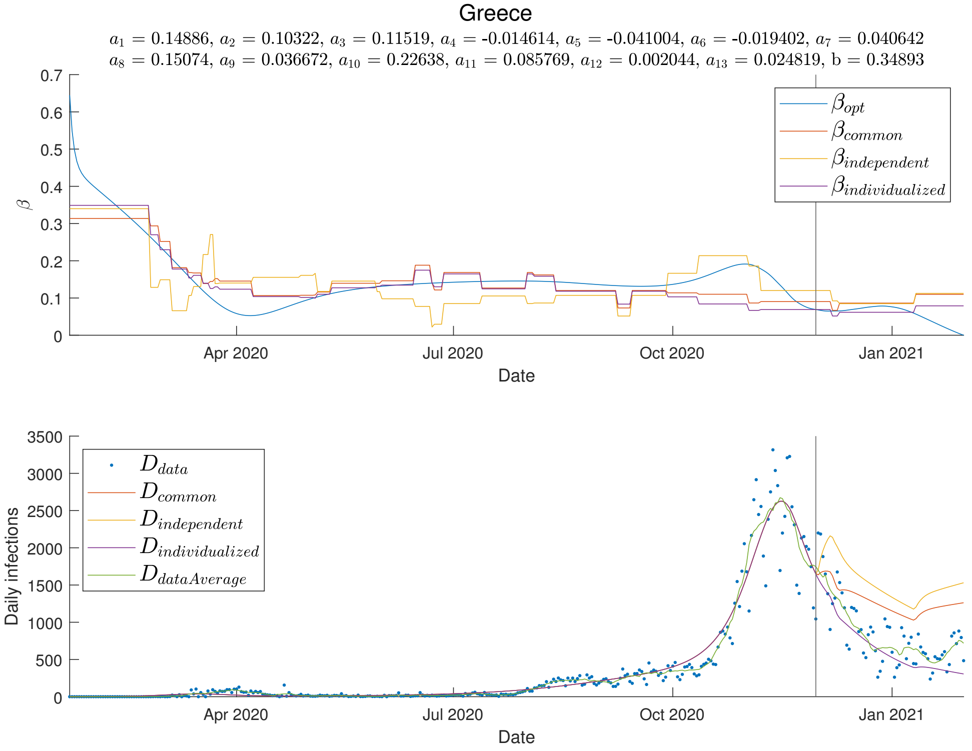

(b) Estimation results. Top panel: fitting a function of restrictions to the signal β_{opt}. Bottom panel: Ability of the model to predict daily infections. The black vertical line indicates the beginning of the validation period. As the number of daily infections is highly variable, a seven day moving average is also presented

Supplementary Figure 58. Prediction results - Greece.

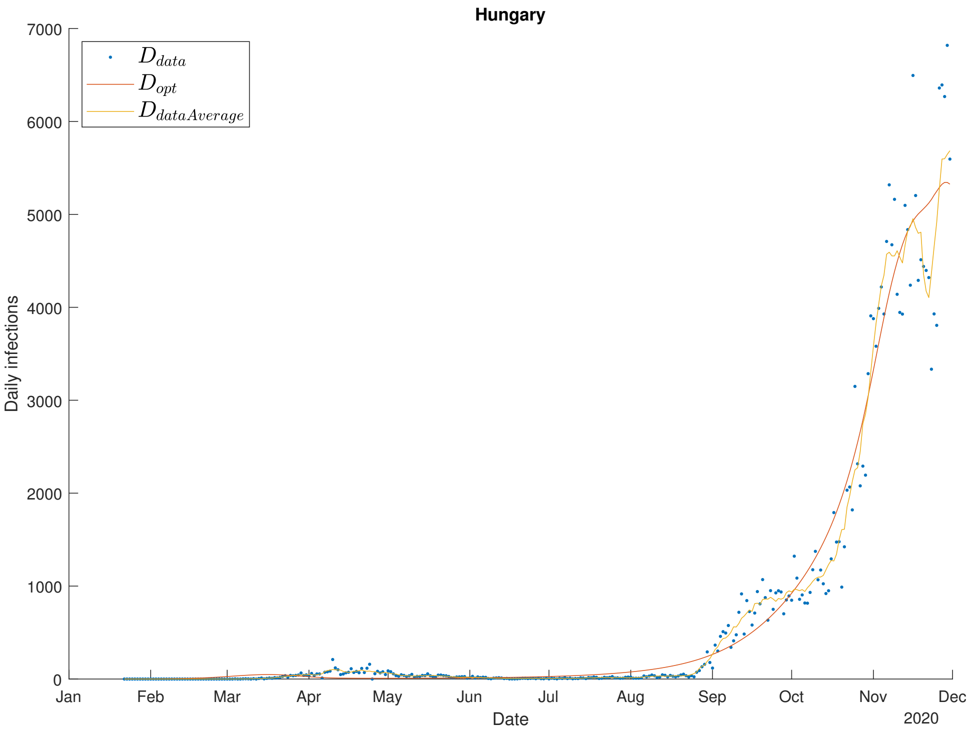

(a) Fit based on $\beta_o pt$ over the training time period. Blue points represent daily infections, the yellow line the seven-day moving average of daily infections, and the red line is the fit obtained by substituting the $\beta_{opt}(t)$ into the SEIR model.

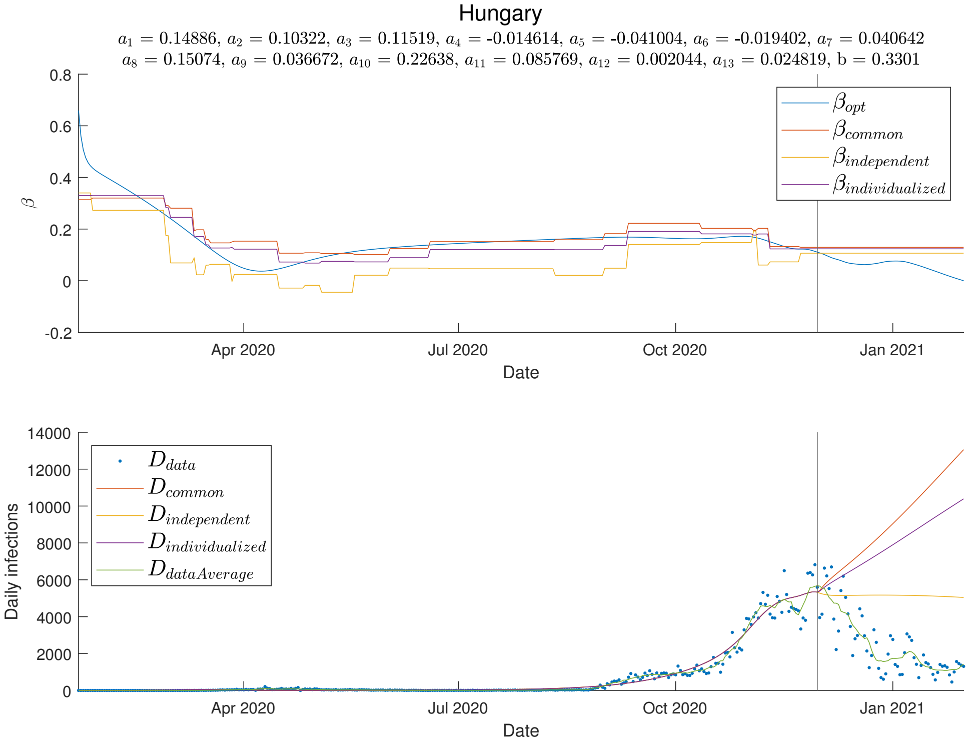

(b) Estimation results. Top panel: fitting a function of restrictions to the signal β_{opt}. Bottom panel: Ability of the model to predict daily infections. The black vertical line indicates the beginning of the validation period. As the number of daily infections is highly variable, a seven day moving average is also presented

Supplementary Figure 59. Prediction results - Hungary.

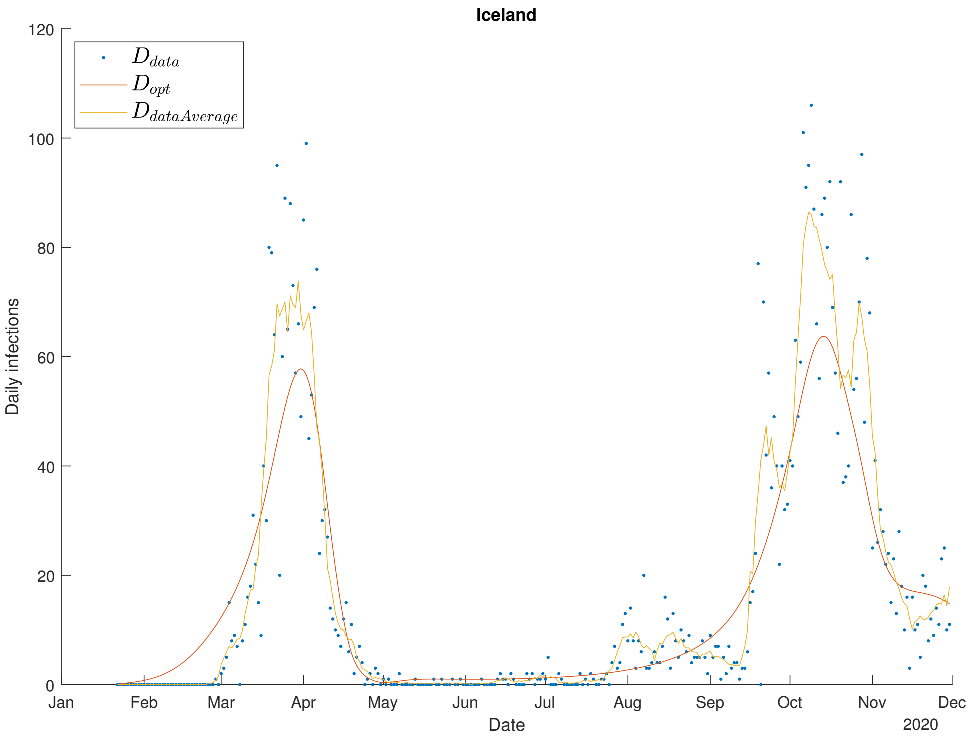

(a) Fit based on $\beta_o pt$ over the training time period. Blue points represent daily infections, the yellow line the seven-day moving average of daily infections, and the red line is the fit obtained by substituting the $\beta_{opt}(t)$ into the SEIR model.

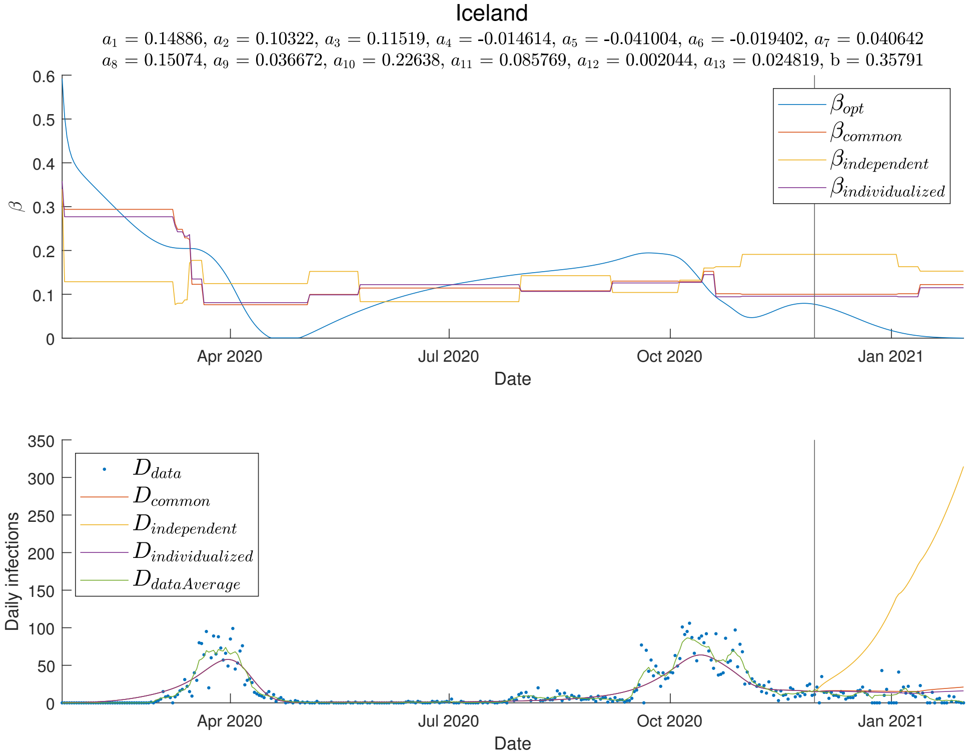

(b) Estimation results. Top panel: fitting a function of restrictions to the signal β_{opt}. Bottom panel: Ability of the model to predict daily infections. The black vertical line indicates the beginning of the validation period. As the number of daily infections is highly variable, a seven day moving average is also presented

Supplementary Figure 60. Prediction results - Iceland.

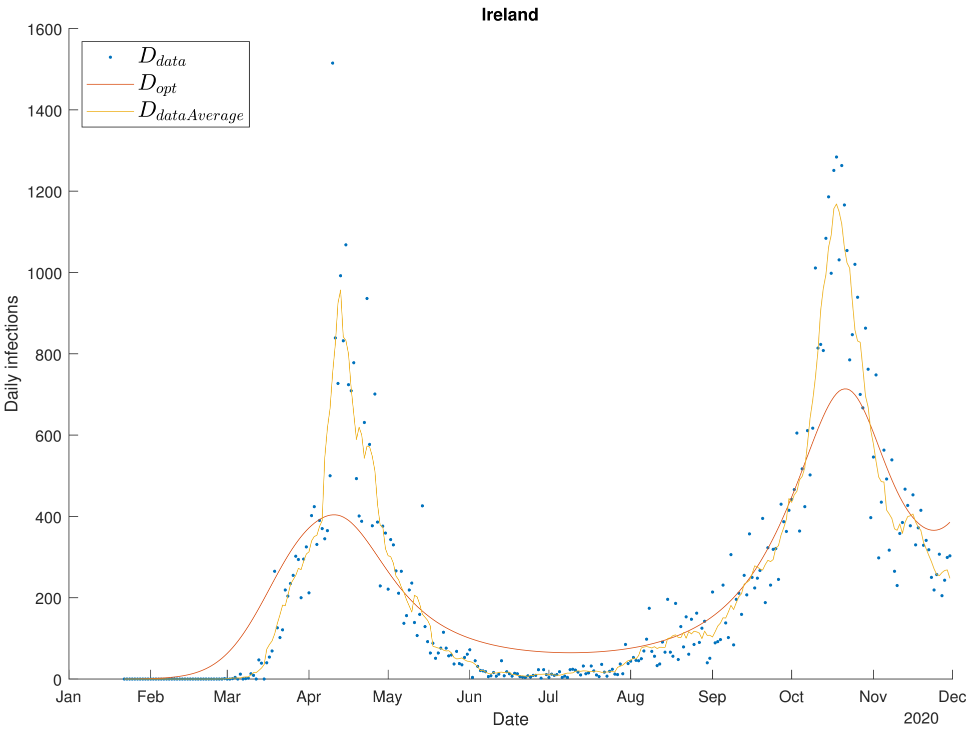

(a) Fit based on $\beta_o pt$ over the training time period. Blue points represent daily infections, the yellow line the seven-day moving average of daily infections, and the red line is the fit obtained by substituting the $\beta_{opt}(t)$ into the SEIR model.

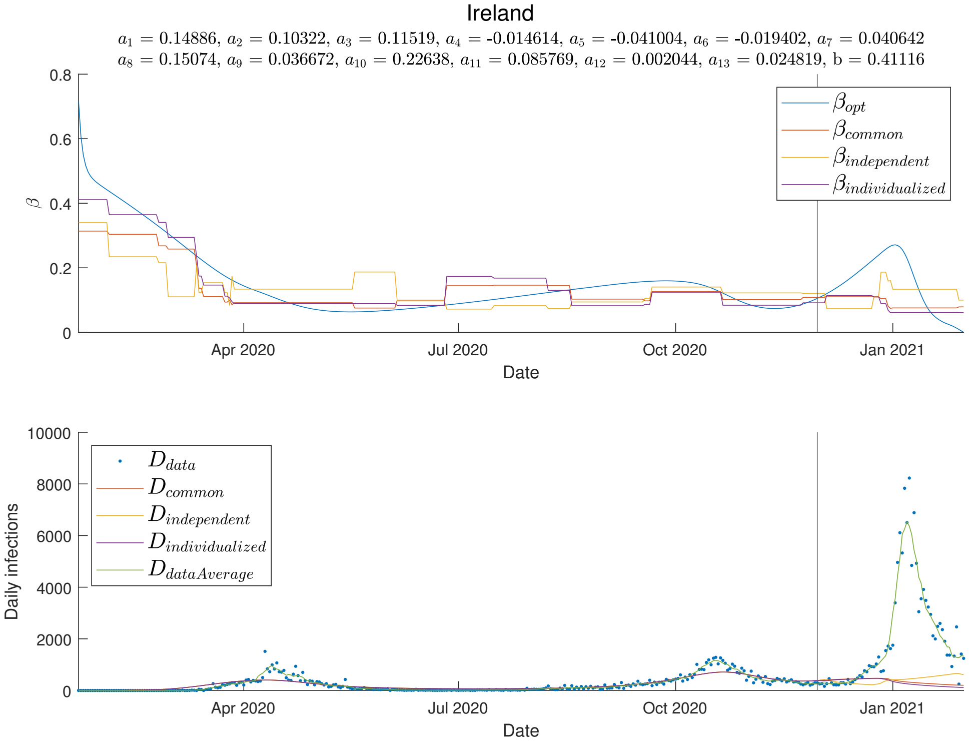

(b) Estimation results. Top panel: fitting a function of restrictions to the signal β_{opt}. Bottom panel: Ability of the model to predict daily infections. The black vertical line indicates the beginning of the validation period. As the number of daily infections is highly variable, a seven day moving average is also presented

Supplementary Figure 61. Prediction results - Ireland.

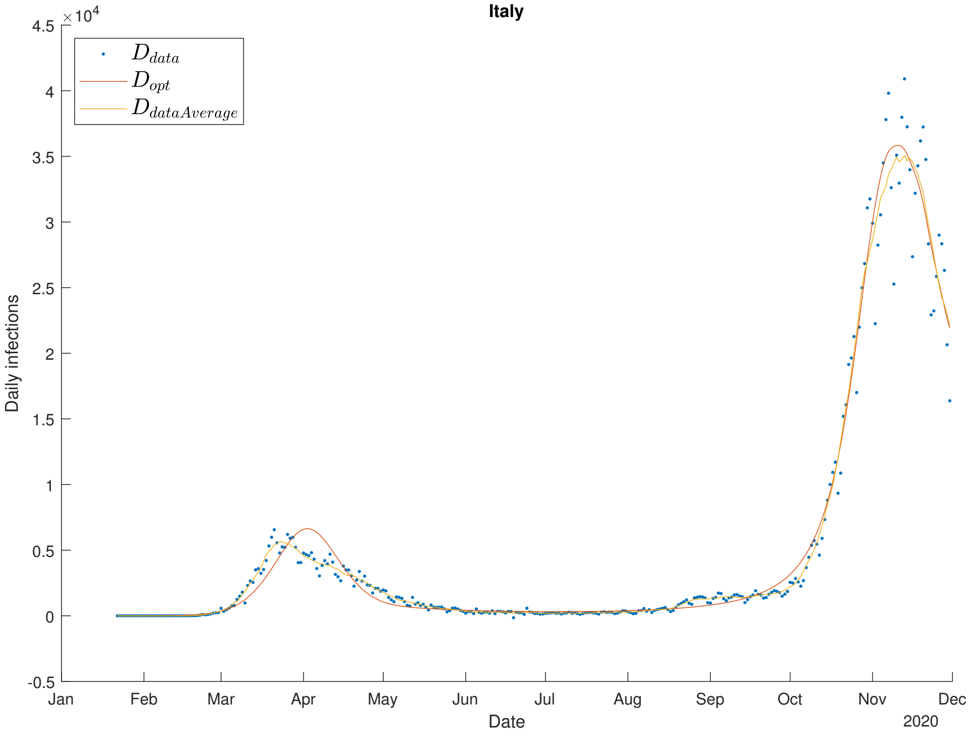

(a) Fit based on $\beta_o pt$ over the training time period. Blue points represent daily infections, the yellow line the seven-day moving average of daily infections, and the red line is the fit obtained by substituting the $\beta_{opt}(t)$ into the SEIR model.

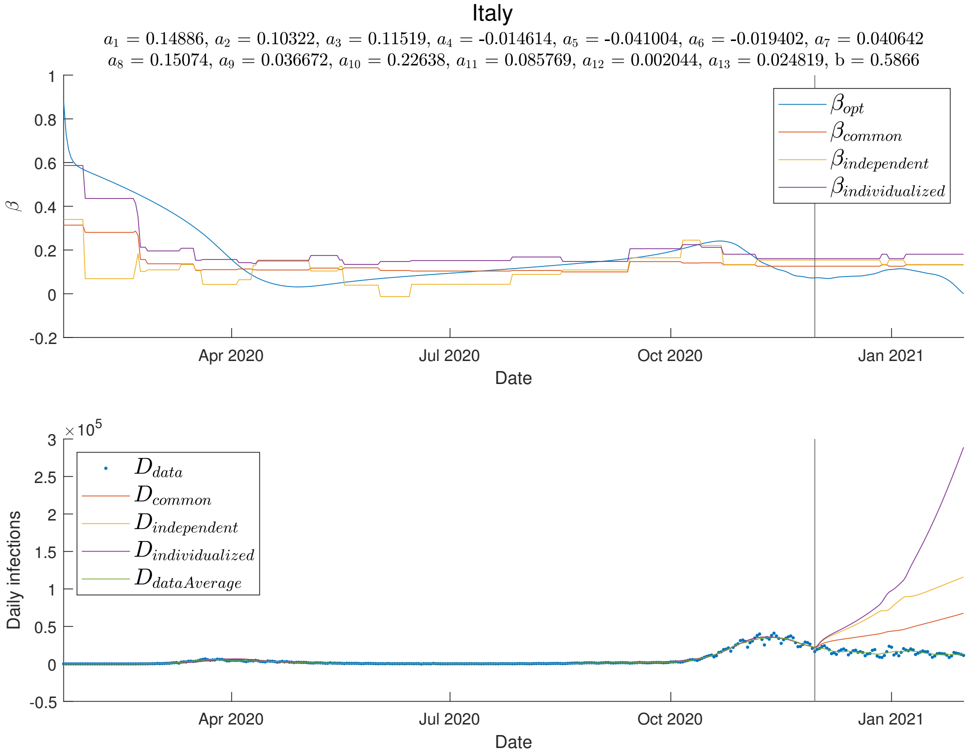

(b) Estimation results. Top panel: fitting a function of restrictions to the signal β_{opt}. Bottom panel: Ability of the model to predict daily infections. The black vertical line indicates the beginning of the validation period. As the number of daily infections is highly variable, a seven day moving average is also presented

Supplementary Figure 62. Prediction results - Italy

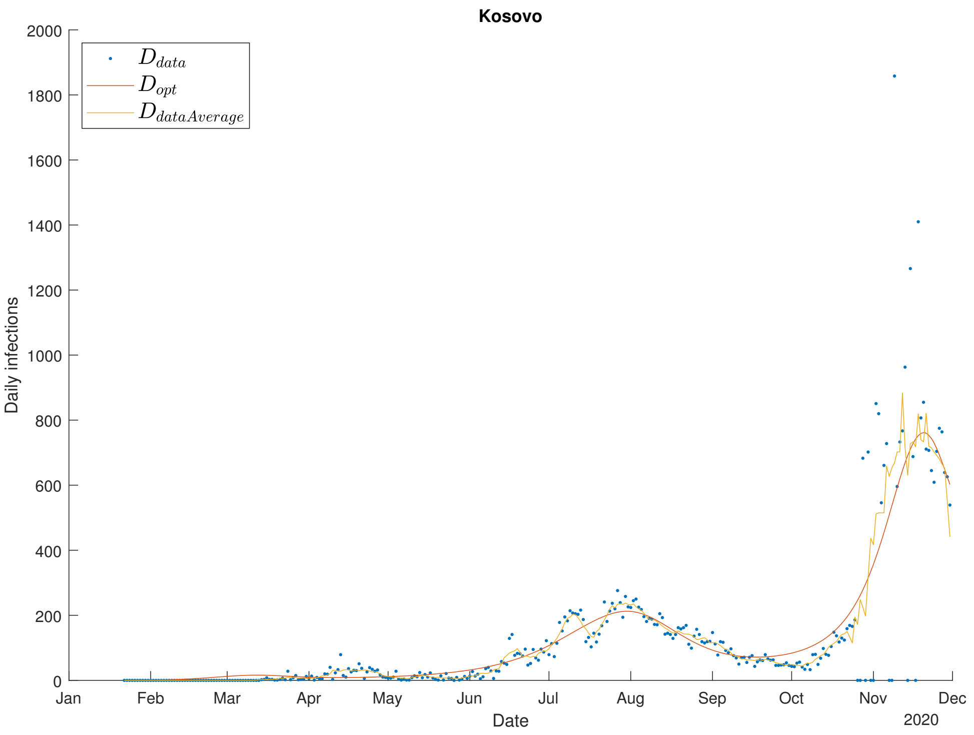

(a) Fit based on $\beta_o pt$ over the training time period. Blue points represent daily infections, the yellow line the seven-day moving average of daily infections, and the red line is the fit obtained by substituting the $\beta_{opt}(t)$ into the SEIR model.

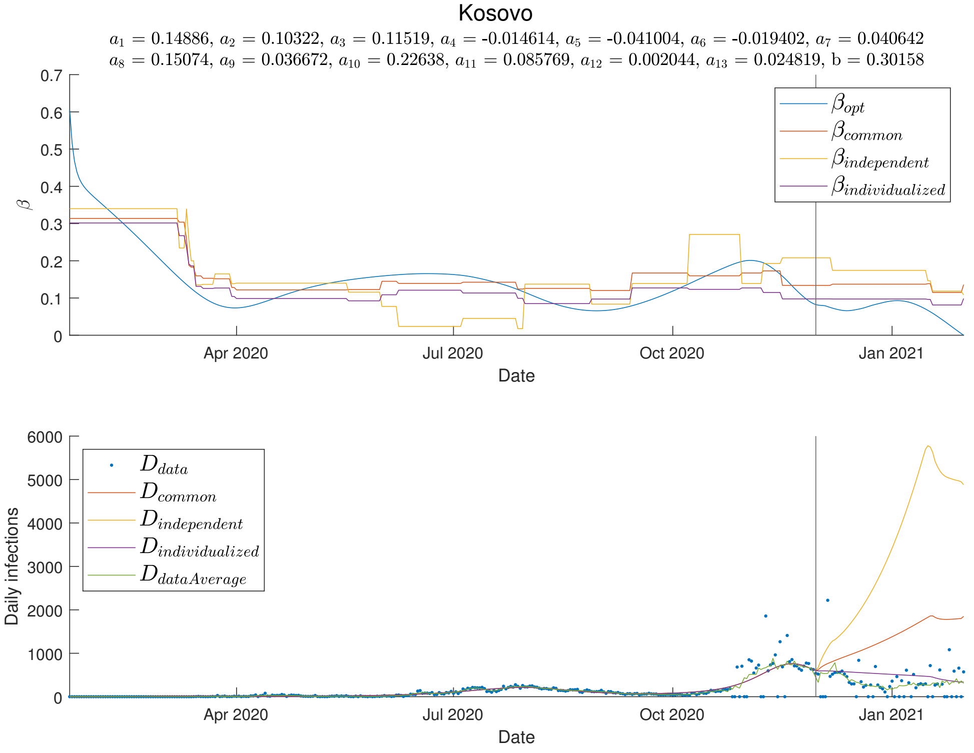

(b) Estimation results. Top panel: fitting a function of restrictions to the signal β_{opt}. Bottom panel: Ability of the model to predict daily infections. The black vertical line indicates the beginning of the validation period. As the number of daily infections is highly variable, a seven day moving average is also presented

Supplementary Figure 63. Prediction results - Kosovo.

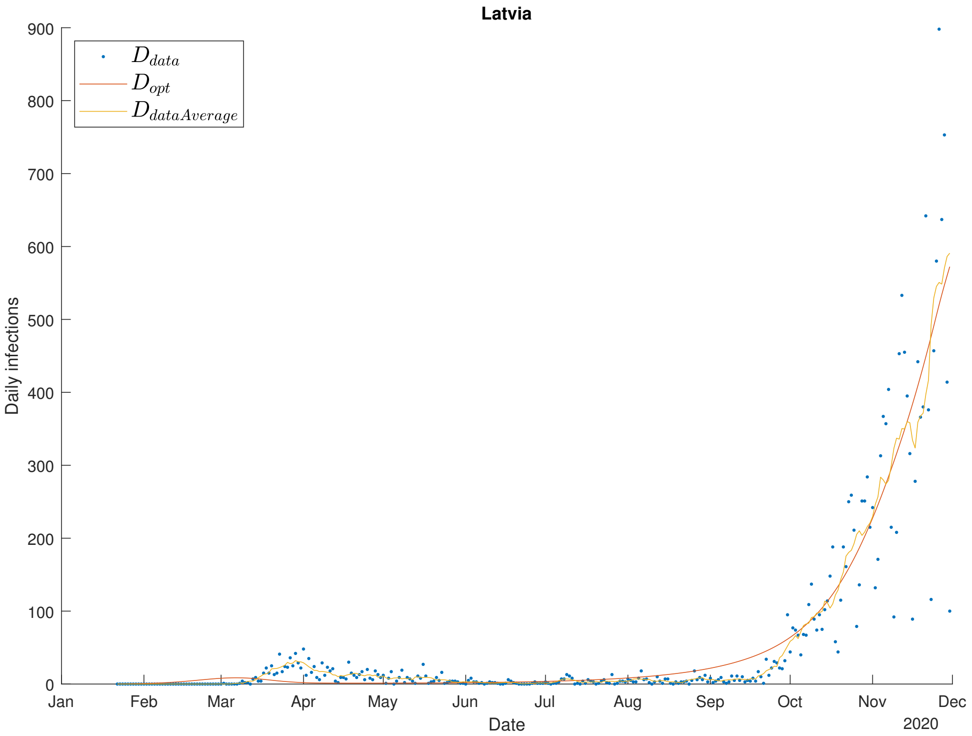

(a) Fit based on $\beta_o pt$ over the training time period. Blue points represent daily infections, the yellow line the seven-day moving average of daily infections, and the red line is the fit obtained by substituting the $\beta_{opt}(t)$ into the SEIR model.

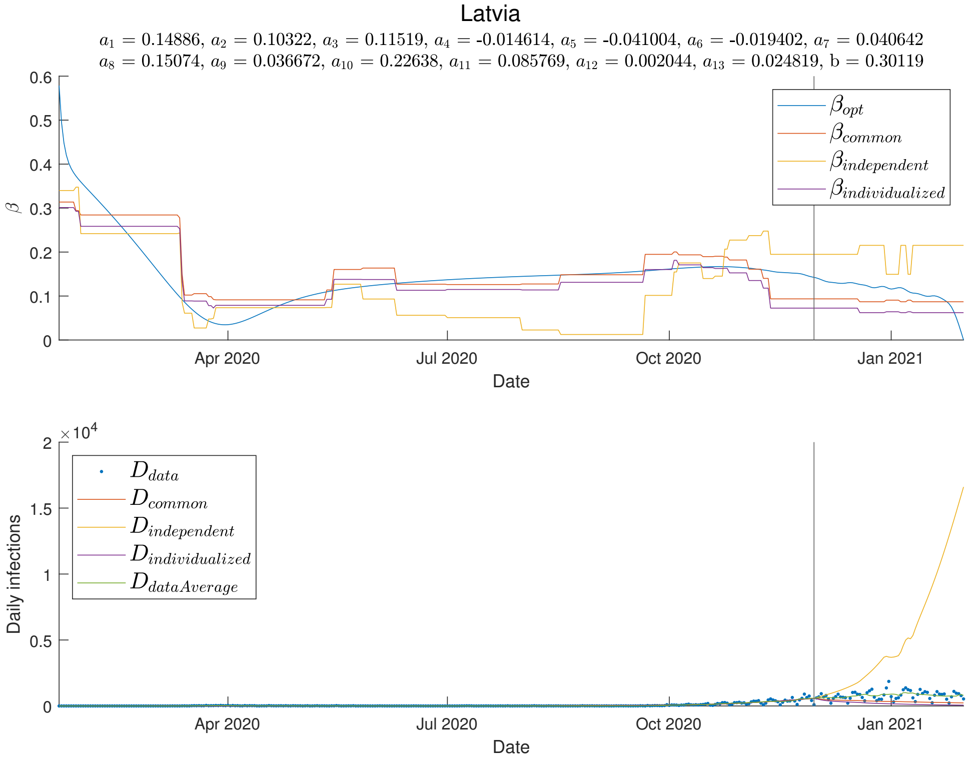

(b) Estimation results. Top panel: fitting a function of restrictions to the signal β_{opt}. Bottom panel: Ability of the model to predict daily infections. The black vertical line indicates the beginning of the validation period. As the number of daily infections is highly variable, a seven day moving average is also presented

Supplementary Figure 64. Prediction results - Latvia.

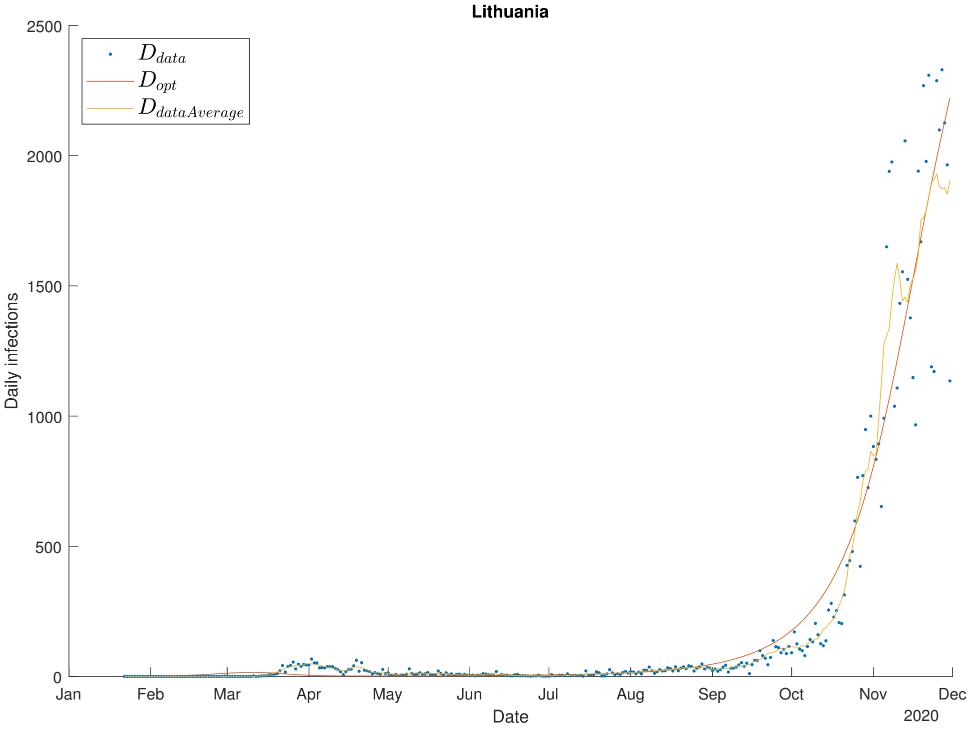

(a) Fit based on $\beta_o pt$ over the training time period. Blue points represent daily infections, the yellow line the seven-day moving average of daily infections, and the red line is the fit obtained by substituting the $\beta_{opt}(t)$ into the SEIR model.

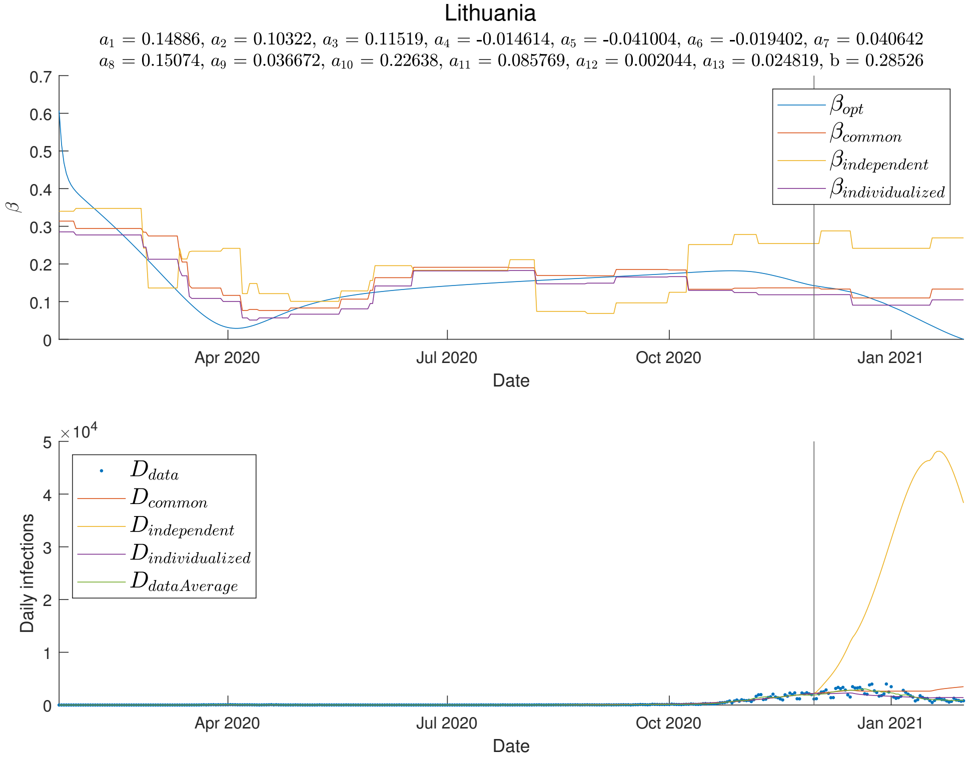

(b) Estimation results. Top panel: fitting a function of restrictions to the signal β_{opt}. Bottom panel: Ability of the model to predict daily infections. The black vertical line indicates the beginning of the validation period. As the number of daily infections is highly variable, a seven day moving average is also presented

Supplementary Figure 65. Prediction results - Lithuania.

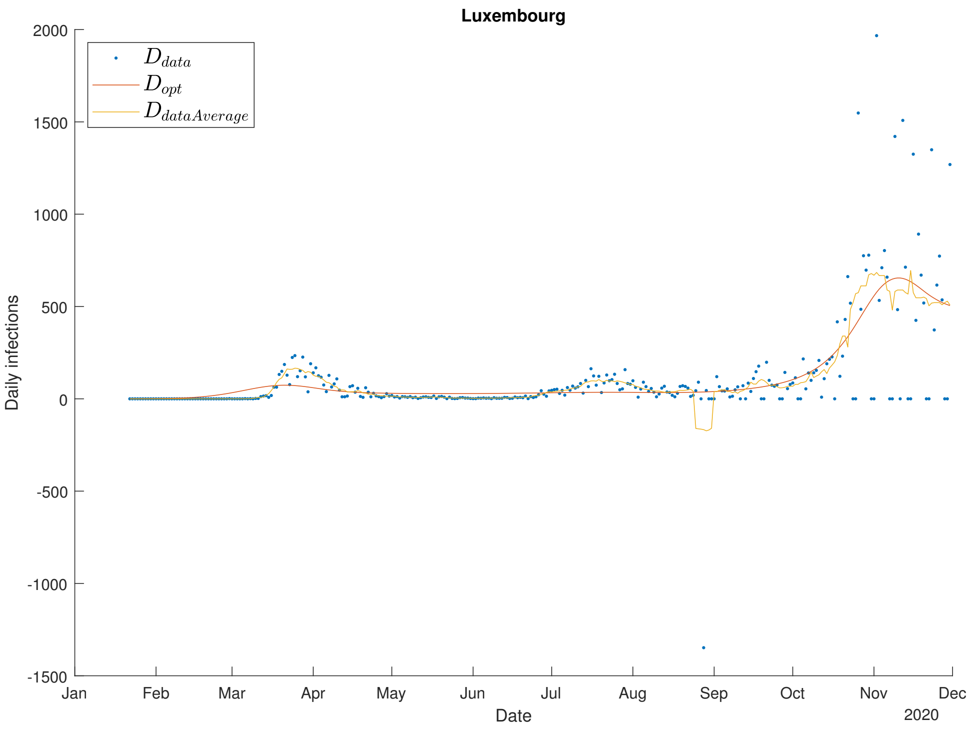

(a) Fit based on $\beta_o pt$ over the training time period. Blue points represent daily infections, the yellow line the seven-day moving average of daily infections, and the red line is the fit obtained by substituting the $\beta_{opt}(t)$ into the SEIR model.

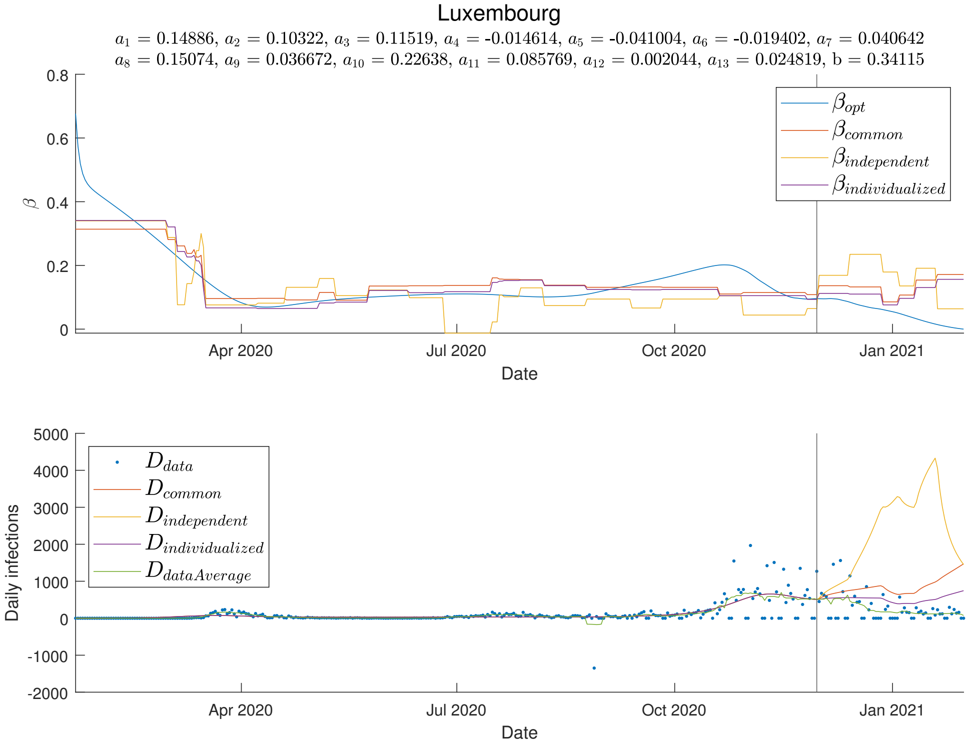

(b) Estimation results. Top panel: fitting a function of restrictions to the signal β_{opt}. Bottom panel: Ability of the model to predict daily infections. The black vertical line indicates the beginning of the validation period. As the number of daily infections is highly variable, a seven day moving average is also presented

Supplementary Figure 66. Prediction results - Luxembourg.

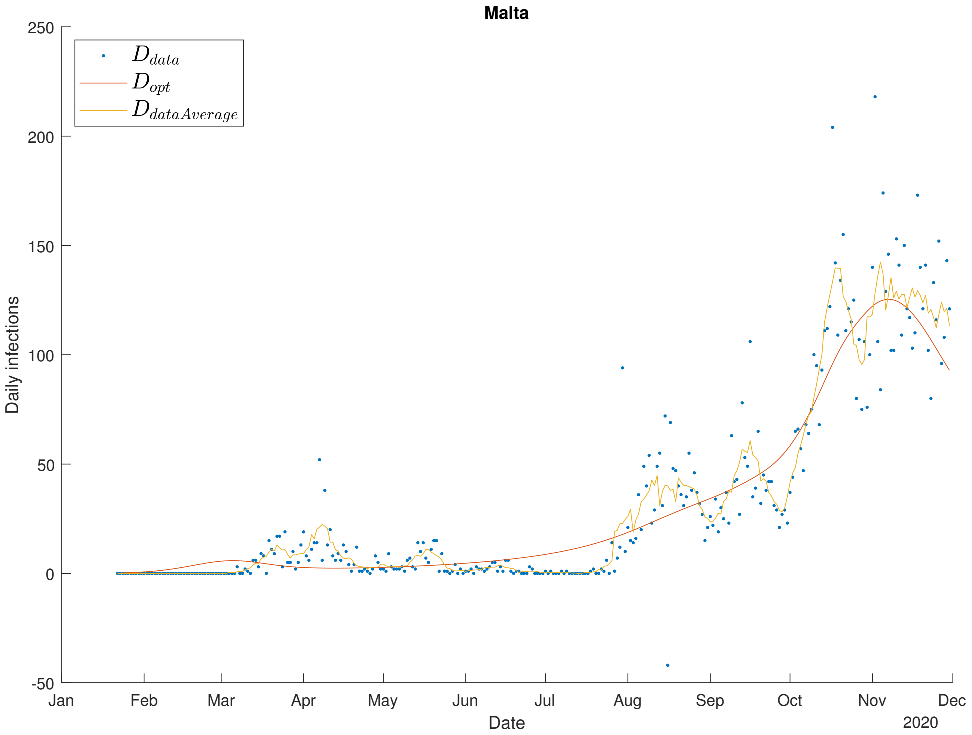

(a) Fit based on $\beta_o pt$ over the training time period. Blue points represent daily infections, the yellow line the seven-day moving average of daily infections, and the red line is the fit obtained by substituting the $\beta_{opt}(t)$ into the SEIR model.

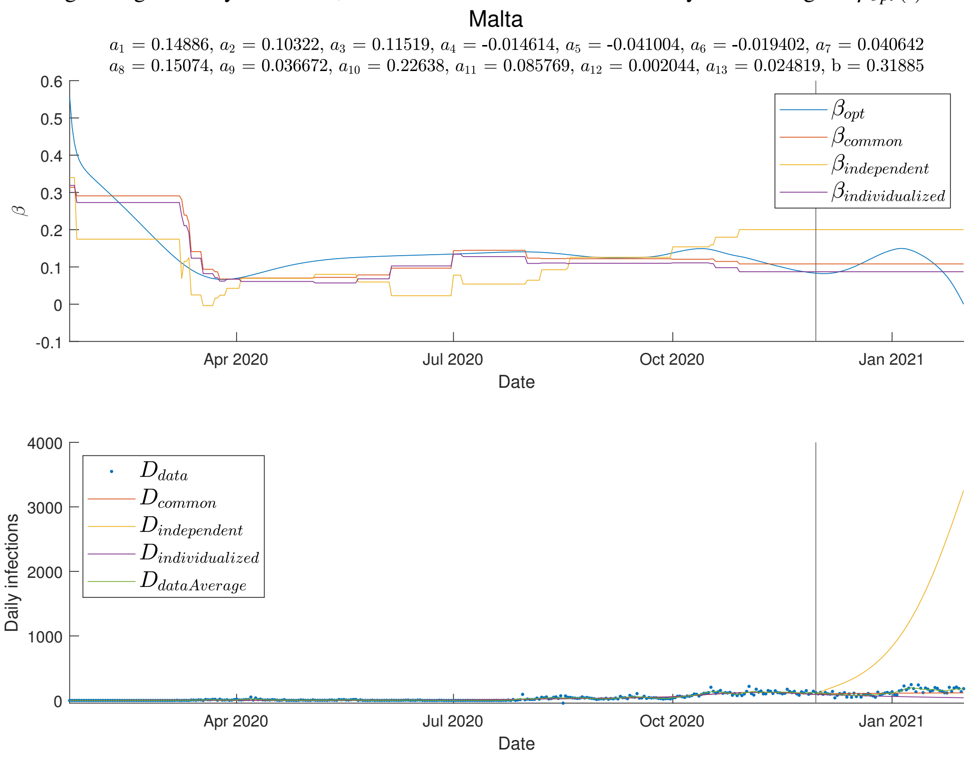

(b) Estimation results. Top panel: fitting a function of restrictions to the signal β_{opt}. Bottom panel: Ability of the model to predict daily infections. The black vertical line indicates the beginning of the validation period. As the number of daily infections is highly variable, a seven day moving average is also presented

Supplementary Figure 67. Prediction results - Malta.

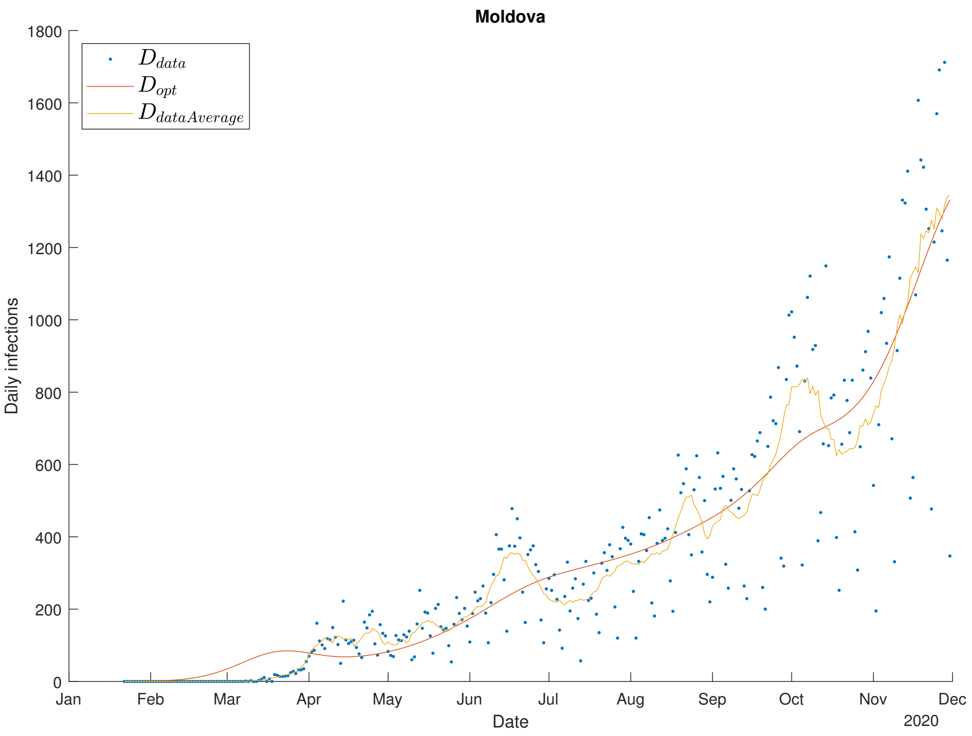

(a) Fit based on $\beta_o pt$ over the training time period. Blue points represent daily infections, the yellow line the seven-day moving average of daily infections, and the red line is the fit obtained by substituting the $\beta_{opt}(t)$ into the SEIR model.

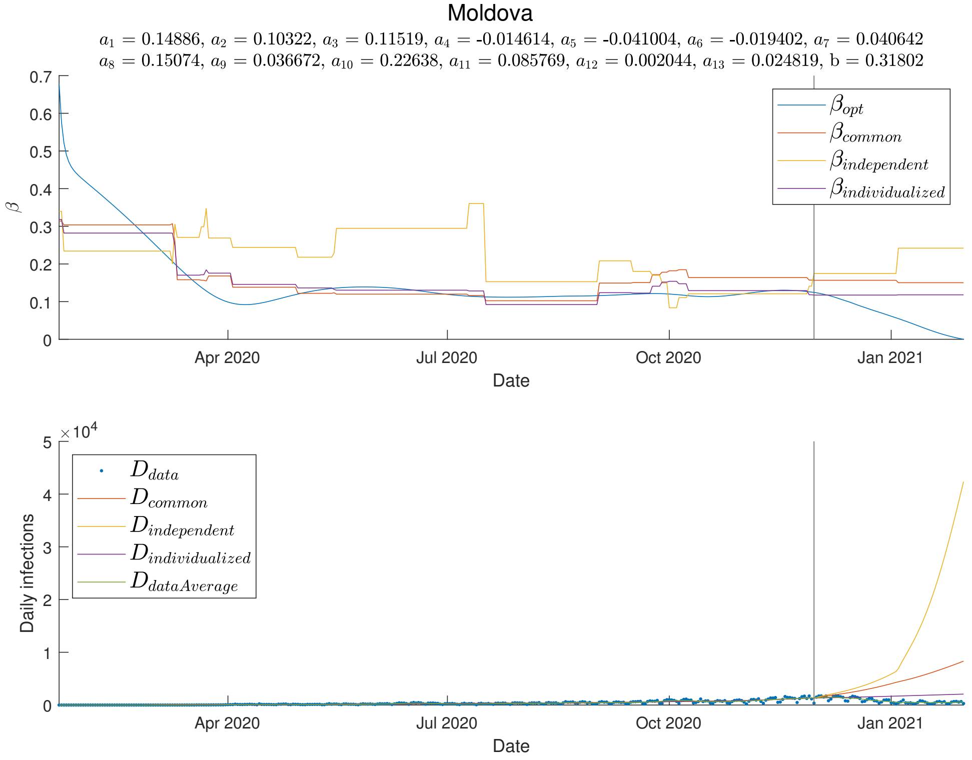

(b) Estimation results. Top panel: fitting a function of restrictions to the signal β_{opt}. Bottom panel: Ability of the model to predict daily infections. The black vertical line indicates the beginning of the validation period. As the number of daily infections is highly variable, a seven day moving average is also presented

Supplementary Figure 68. Prediction results - Moldova.

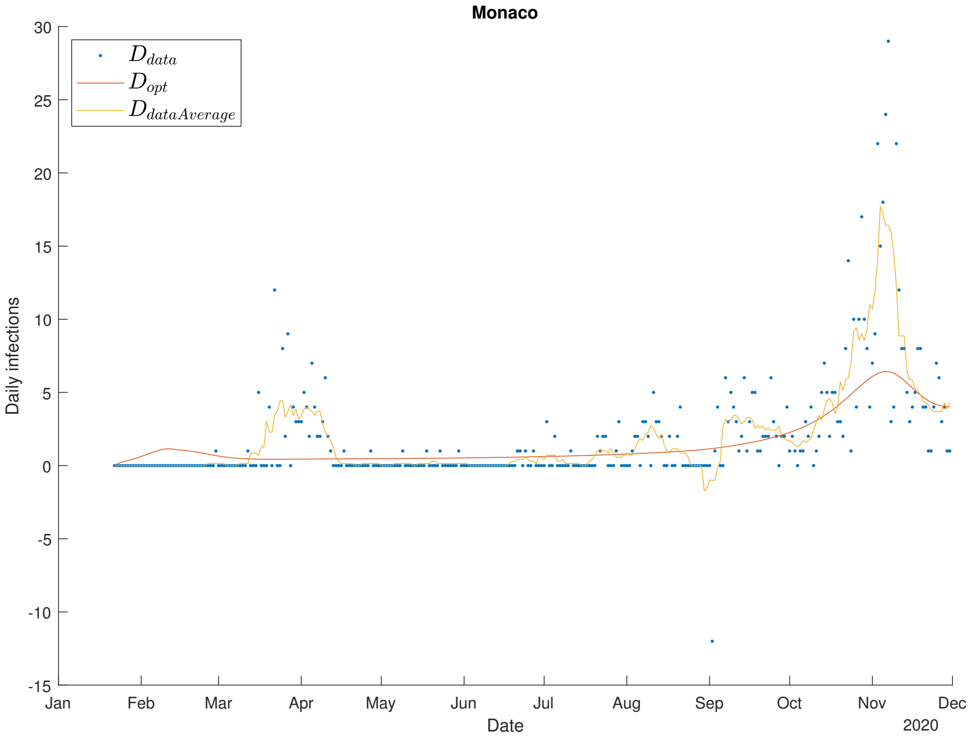

(a) Fit based on $\beta_o pt$ over the training time period. Blue points represent daily infections, the yellow line the seven-day moving average of daily infections, and the red line is the fit obtained by substituting the $\beta_{opt}(t)$ into the SEIR model.

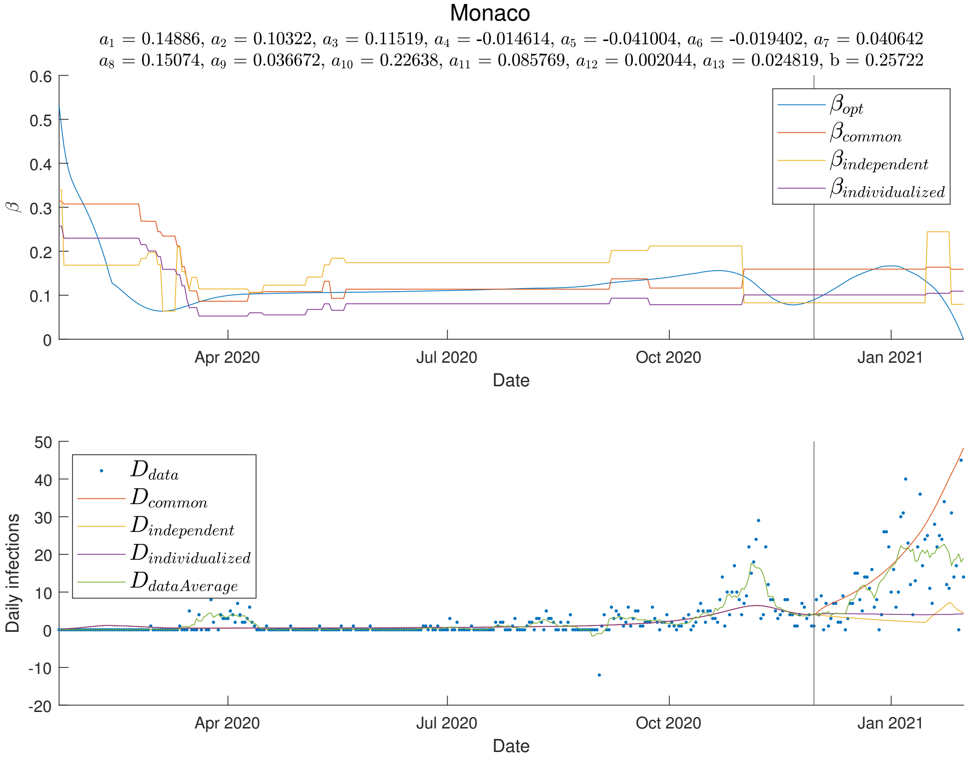

(b) Estimation results. Top panel: fitting a function of restrictions to the signal β_{opt}. Bottom panel: Ability of the model to predict daily infections. The black vertical line indicates the beginning of the validation period. As the number of daily infections is highly variable, a seven day moving average is also presented

Supplementary Figure 69. Prediction results - Monaco.

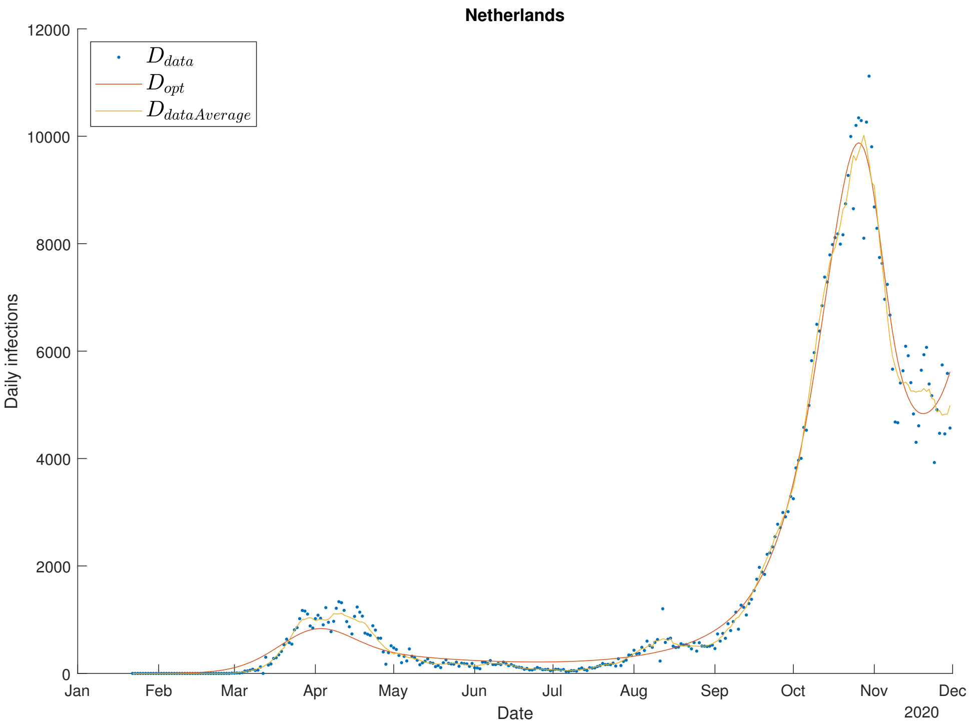

(a) Fit based on $\beta_o pt$ over the training time period. Blue points represent daily infections, the yellow line the seven-day moving average of daily infections, and the red line is the fit obtained by substituting the $\beta_{opt}(t)$ into the SEIR model.

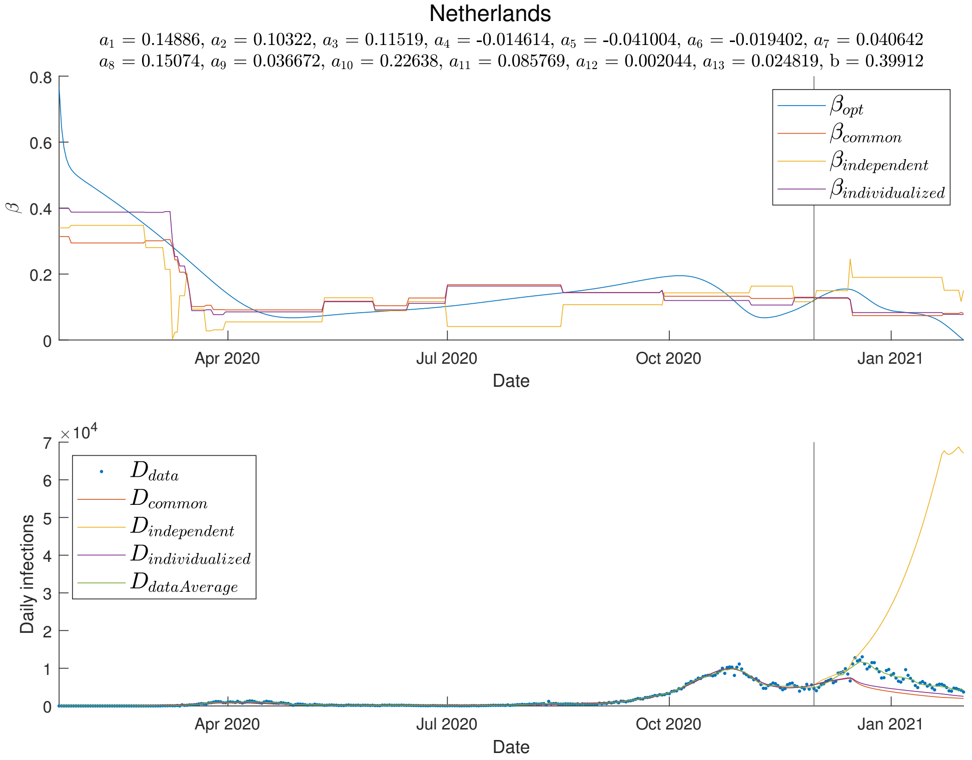

(b) Estimation results. Top panel: fitting a function of restrictions to the signal β_{opt}. Bottom panel: Ability of the model to predict daily infections. The black vertical line indicates the beginning of the validation period. As the number of daily infections is highly variable, a seven day moving average is also presented

Supplementary Figure 70. Prediction results - Netherlands.

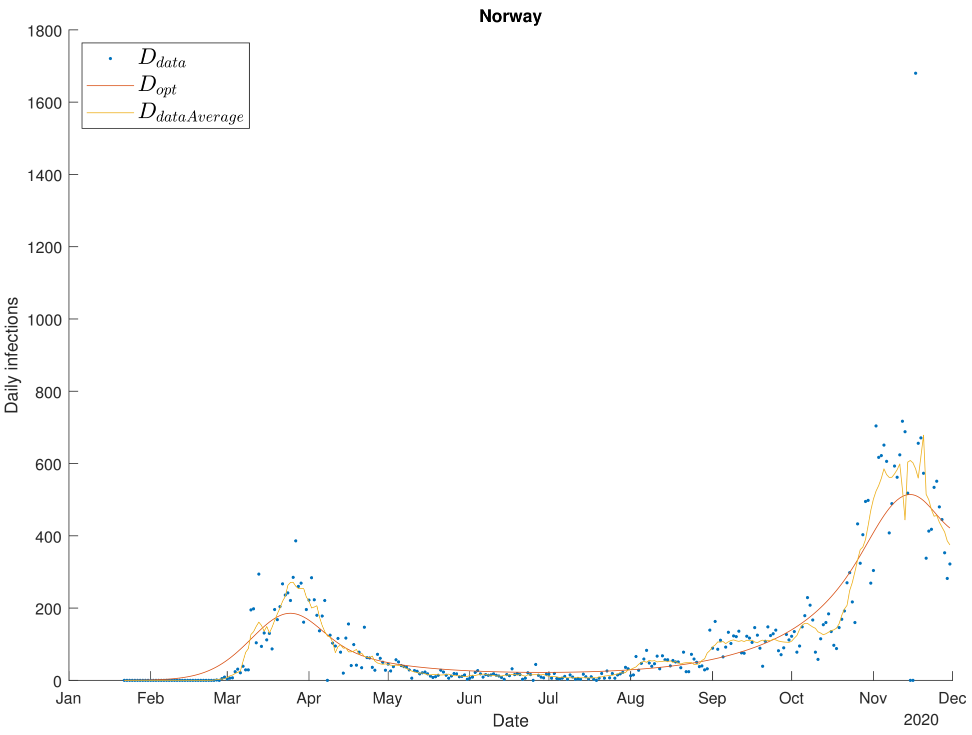

(a) Fit based on $\beta_o pt$ over the training time period. Blue points represent daily infections, the yellow line the seven-day moving average of daily infections, and the red line is the fit obtained by substituting the $\beta_{opt}(t)$ into the SEIR model.

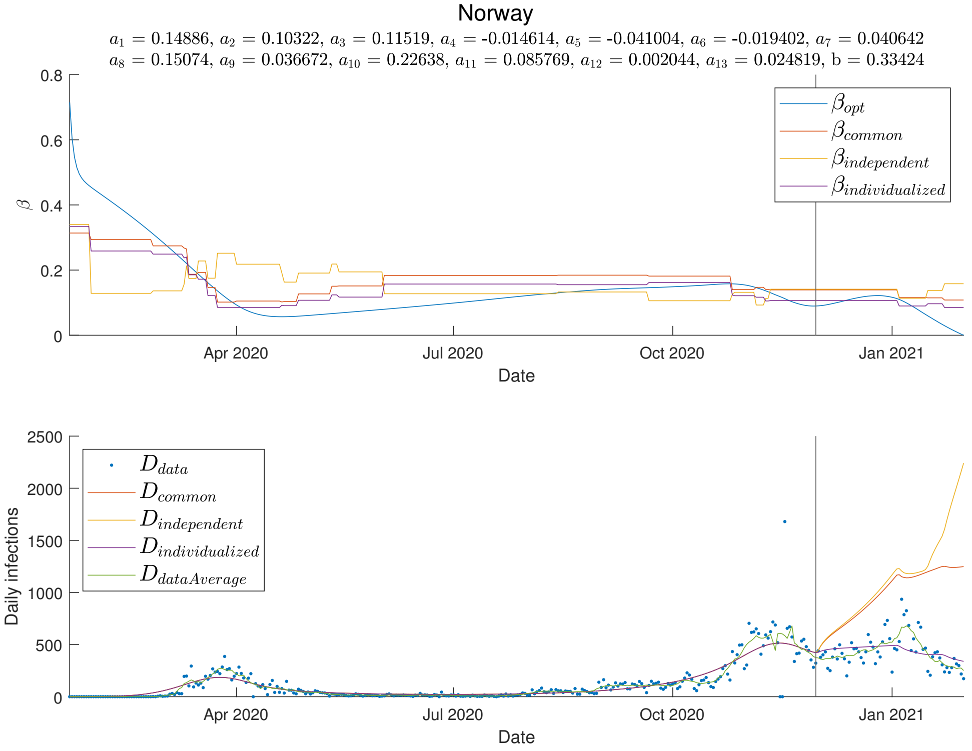

(b) Estimation results. Top panel: fitting a function of restrictions to the signal β_{opt}. Bottom panel: Ability of the model to predict daily infections. The black vertical line indicates the beginning of the validation period. As the number of daily infections is highly variable, a seven day moving average is also presented

Supplementary Figure 71. Prediction results - Norway.

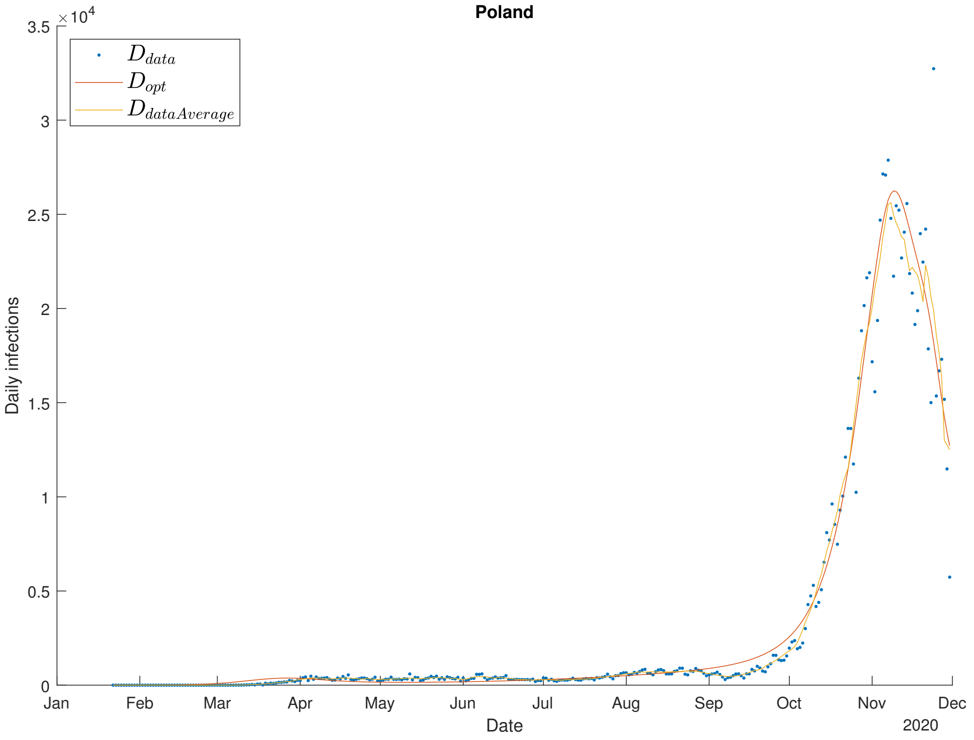

(a) Fit based on $\beta_o pt$ over the training time period. Blue points represent daily infections, the yellow line the seven-day moving average of daily infections, and the red line is the fit obtained by substituting the $\beta_{opt}(t)$ into the SEIR model.

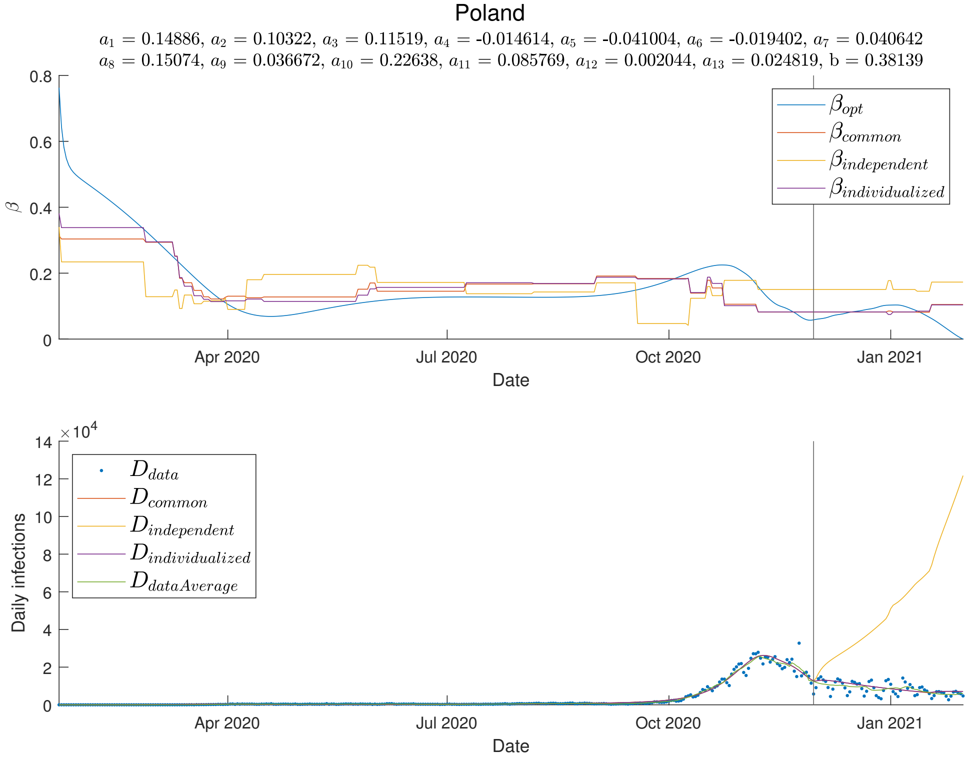

(b) Estimation results. Top panel: fitting a function of restrictions to the signal β_{opt}. Bottom panel: Ability of the model to predict daily infections. The black vertical line indicates the beginning of the validation period. As the number of daily infections is highly variable, a seven day moving average is also presented

Supplementary Figure 72. Prediction results - Poland.

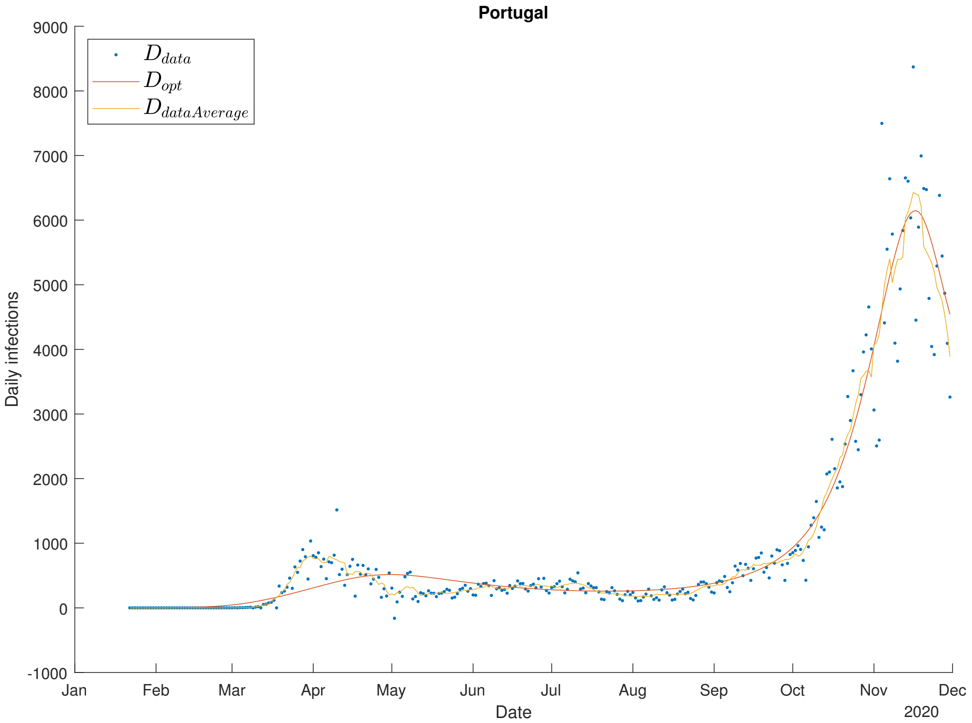

(a) Fit based on $\beta_o pt$ over the training time period. Blue points represent daily infections, the yellow line the seven-day moving average of daily infections, and the red line is the fit obtained by substituting the $\beta_{opt}(t)$ into the SEIR model.

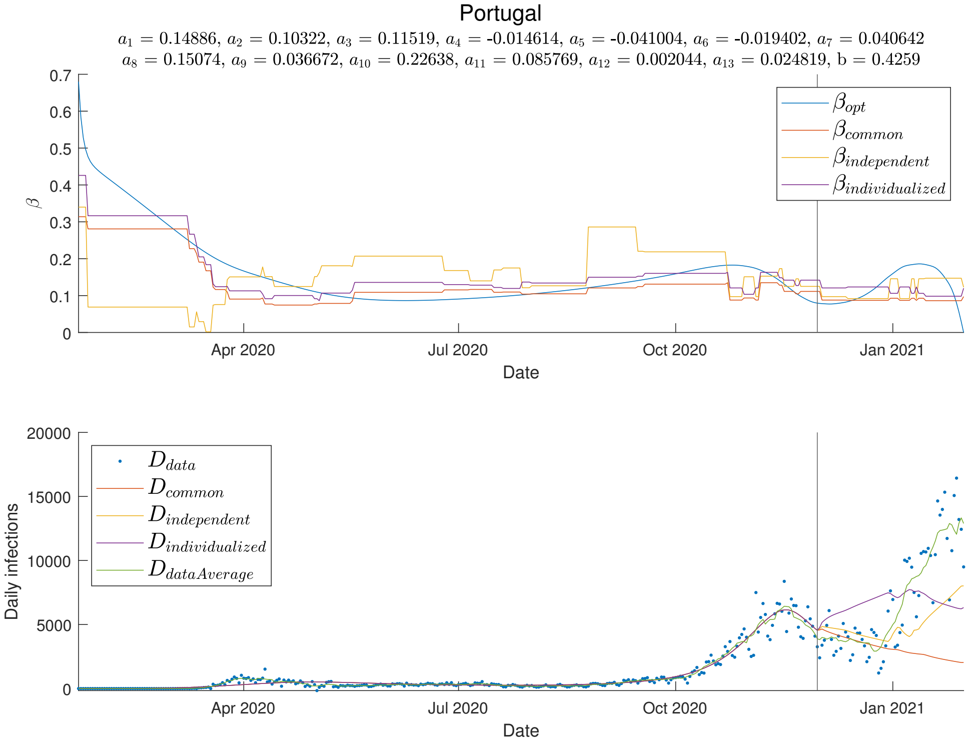

(b) Estimation results. Top panel: fitting a function of restrictions to the signal β_{opt}. Bottom panel: Ability of the model to predict daily infections. The black vertical line indicates the beginning of the validation period. As the number of daily infections is highly variable, a seven day moving average is also presented

Supplementary Figure 73. Prediction results - Portugal.

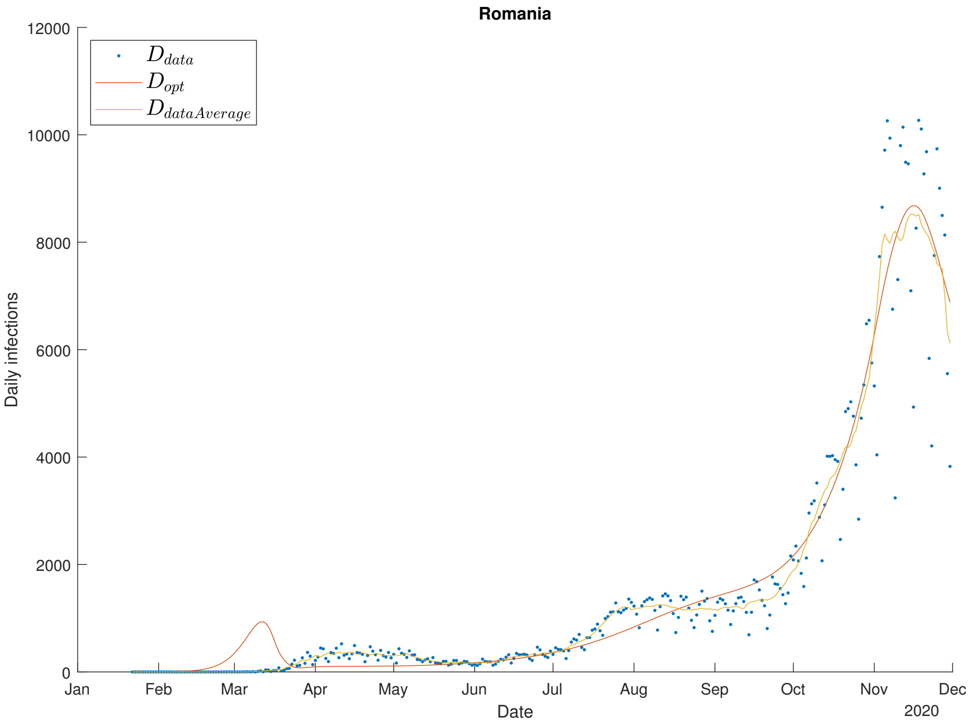

(a) Fit based on $\beta_o pt$ over the training time period. Blue points represent daily infections, the yellow line the seven-day moving average of daily infections, and the red line is the fit obtained by substituting the $\beta_{opt}(t)$ into the SEIR model.

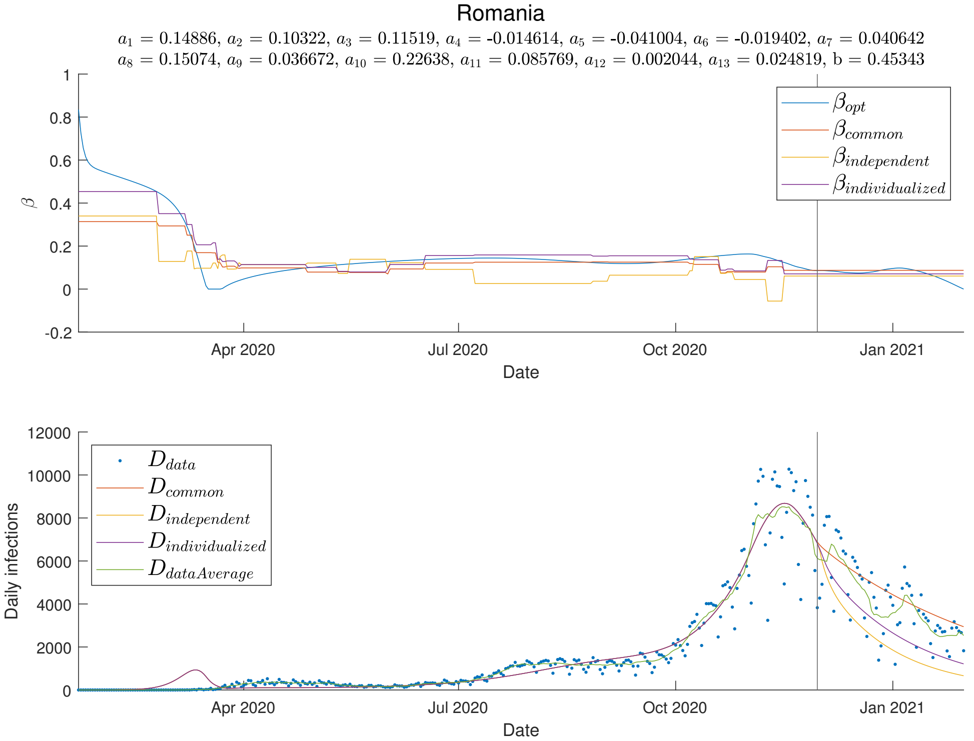

(b) Estimation results. Top panel: fitting a function of restrictions to the signal β_{opt}. Bottom panel: Ability of the model to predict daily infections. The black vertical line indicates the beginning of the validation period. As the number of daily infections is highly variable, a seven day moving average is also presented

Supplementary Figure 74. Prediction results - Romania.

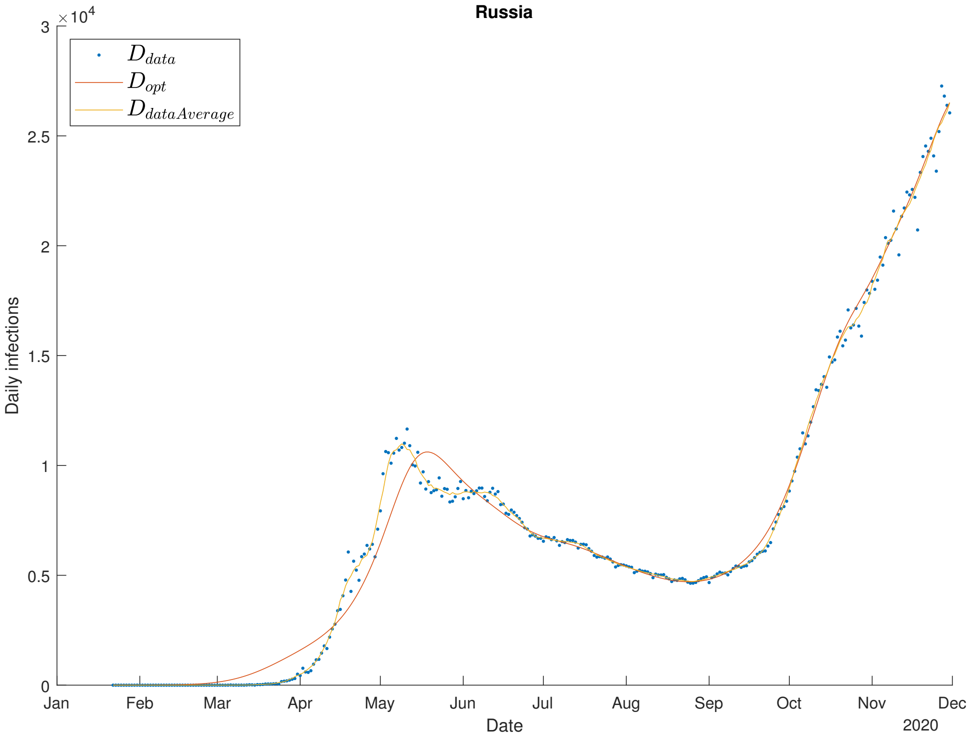

(a) Fit based on $\beta_o pt$ over the training time period. Blue points represent daily infections, the yellow line the seven-day moving average of daily infections, and the red line is the fit obtained by substituting the $\beta_{opt}(t)$ into the SEIR model.

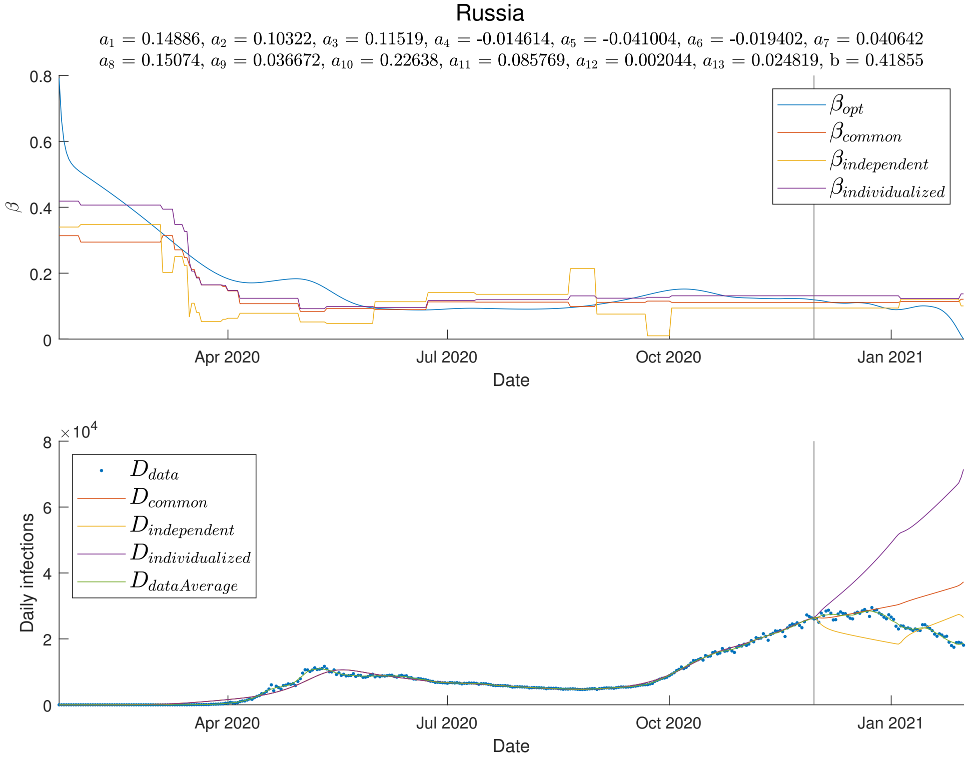

(b) Estimation results. Top panel: fitting a function of restrictions to the signal β_{opt}. Bottom panel: Ability of the model to predict daily infections. The black vertical line indicates the beginning of the validation period. As the number of daily infections is highly variable, a seven day moving average is also presented

Supplementary Figure 75. Prediction results - Russia.

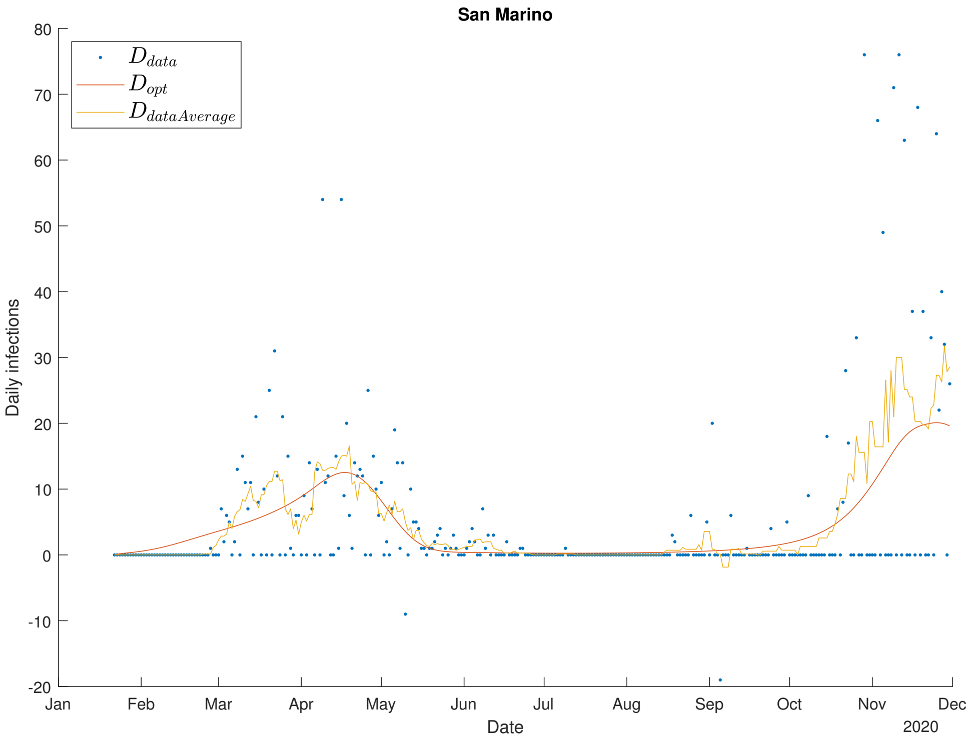

(a) Fit based on β_opt over the training time period. Blue points represent daily infections, the yellow line the seven-day moving average of daily infections, and the red line is the fit obtained by substituting the $\beta_{opt}(t)$ into the SEIR model.

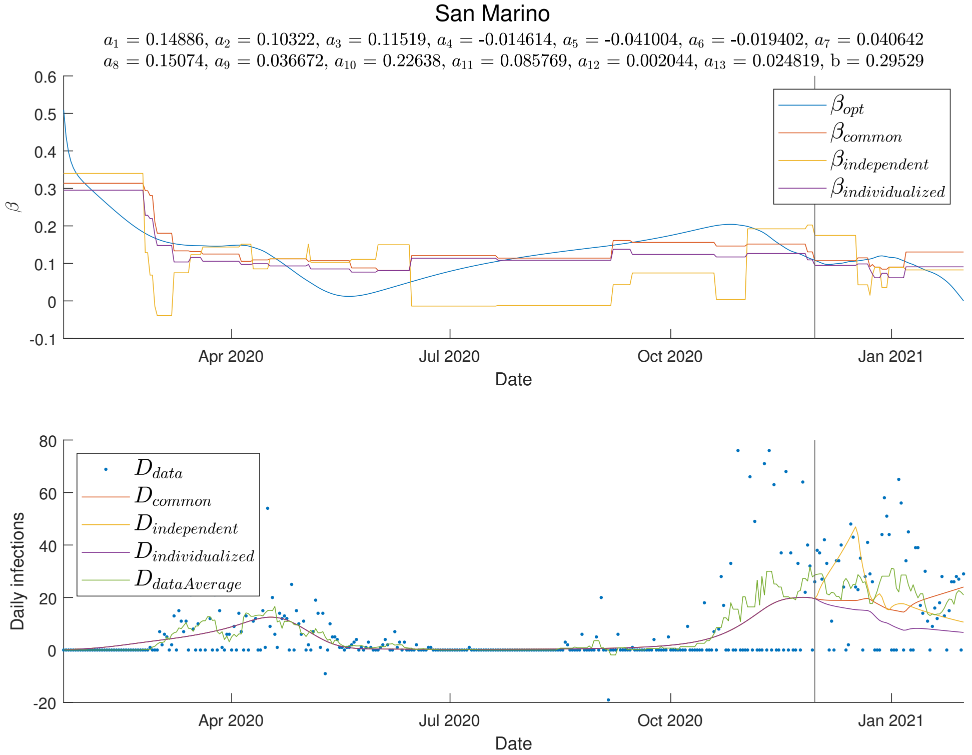

(b) Estimation results. Top panel: fitting a function of restrictions to the signal β_{opt}. Bottom panel: Ability of the model to predict daily infections. The black vertical line indicates the beginning of the validation period. As the number of daily infections is highly variable, a seven day moving average is also presented

Supplementary Figure 76. Prediction results - San Marino.

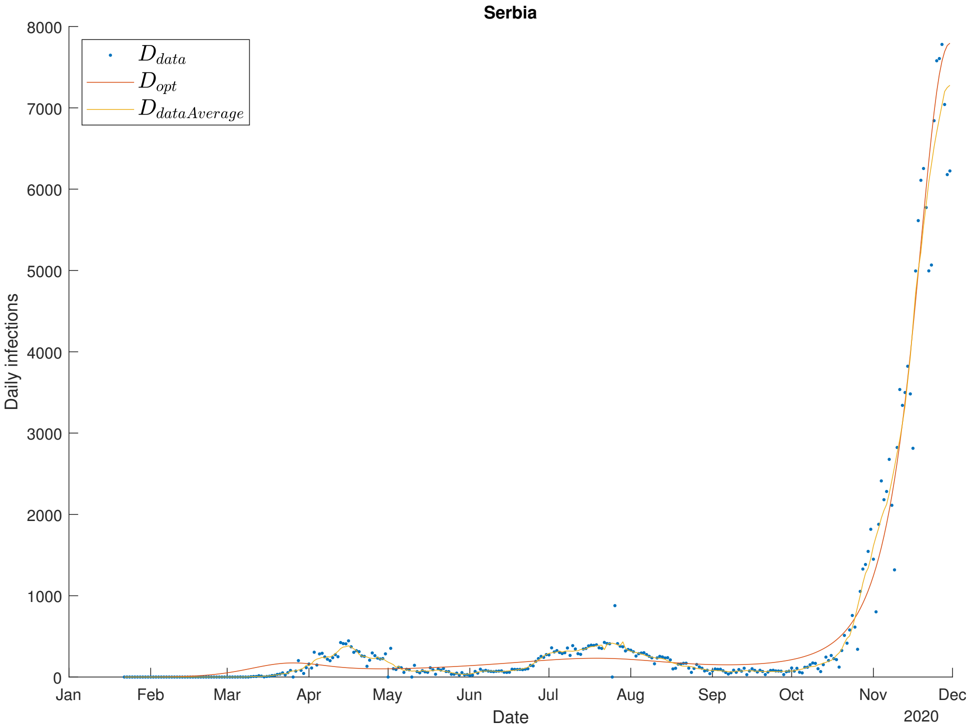

(a) Fit based on β_opt over the training time period. Blue points represent daily infections, the yellow line the seven-day moving average of daily infections, and the red line is the fit obtained by substituting the $\beta_{opt}(t)$ into the SEIR model.

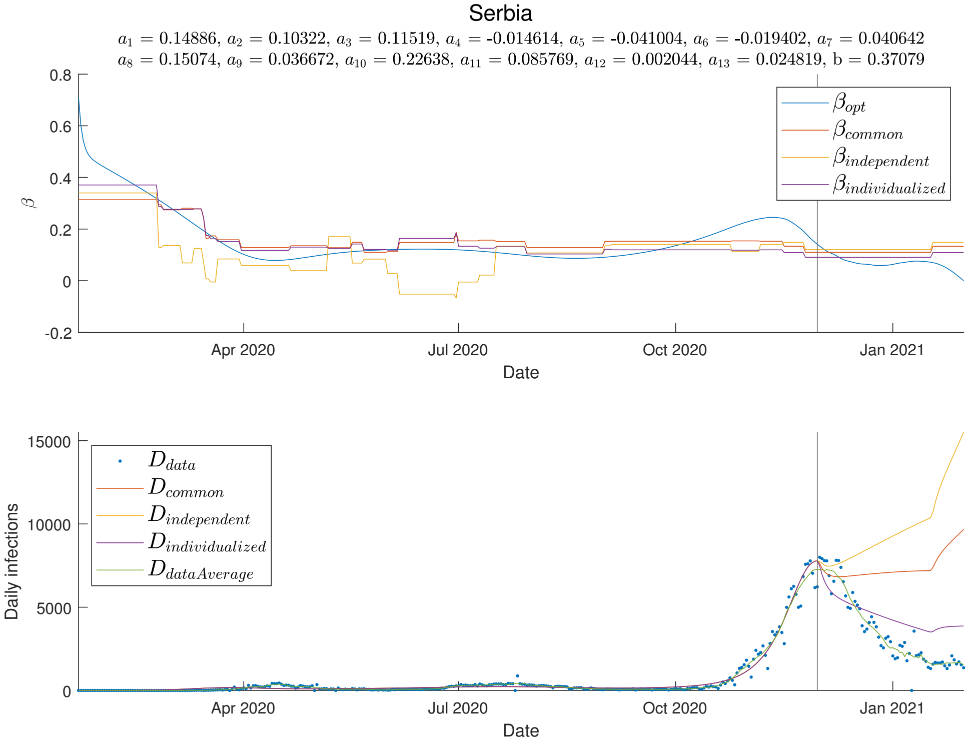

(b) Estimation results. Top panel: fitting a function of restrictions to the signal β_{opt}. Bottom panel: Ability of the model to predict daily infections. The black vertical line indicates the beginning of the validation period. As the number of daily infections is highly variable, a seven day moving average is also presented

Supplementary Figure 77. Prediction results - Serbia.

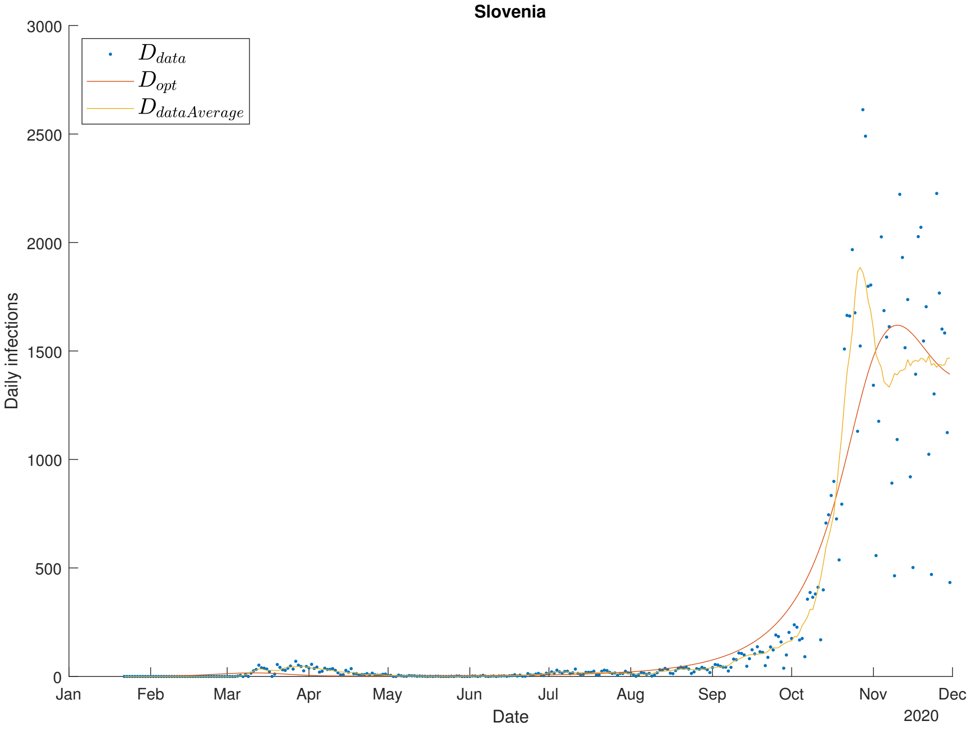

(a) Fit based on $\beta_o pt$ over the training time period. Blue points represent daily infections, the yellow line the seven-day moving average of daily infections, and the red line is the fit obtained by substituting the $\beta_{opt}(t)$ into the SEIR model.

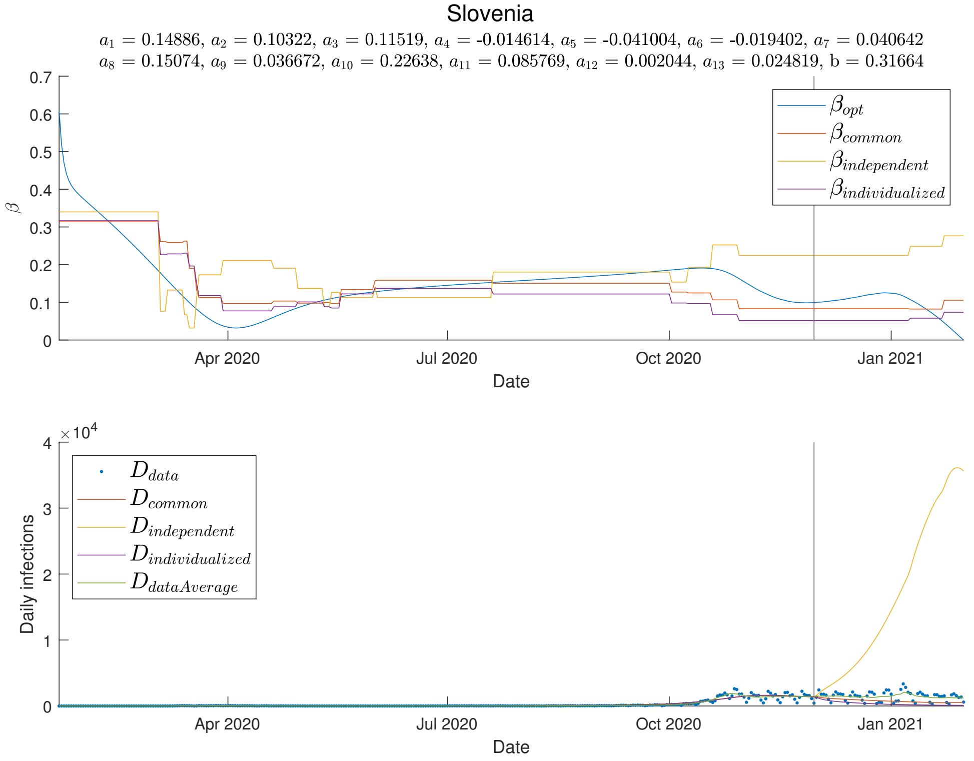

(b) Estimation results. Top panel: fitting a function of restrictions to the signal β_{opt}. Bottom panel: Ability of the model to predict daily infections. The black vertical line indicates the beginning of the validation period. As the number of daily infections is highly variable, a seven day moving average is also presented

Supplementary Figure 78. Prediction results - Slovenia.

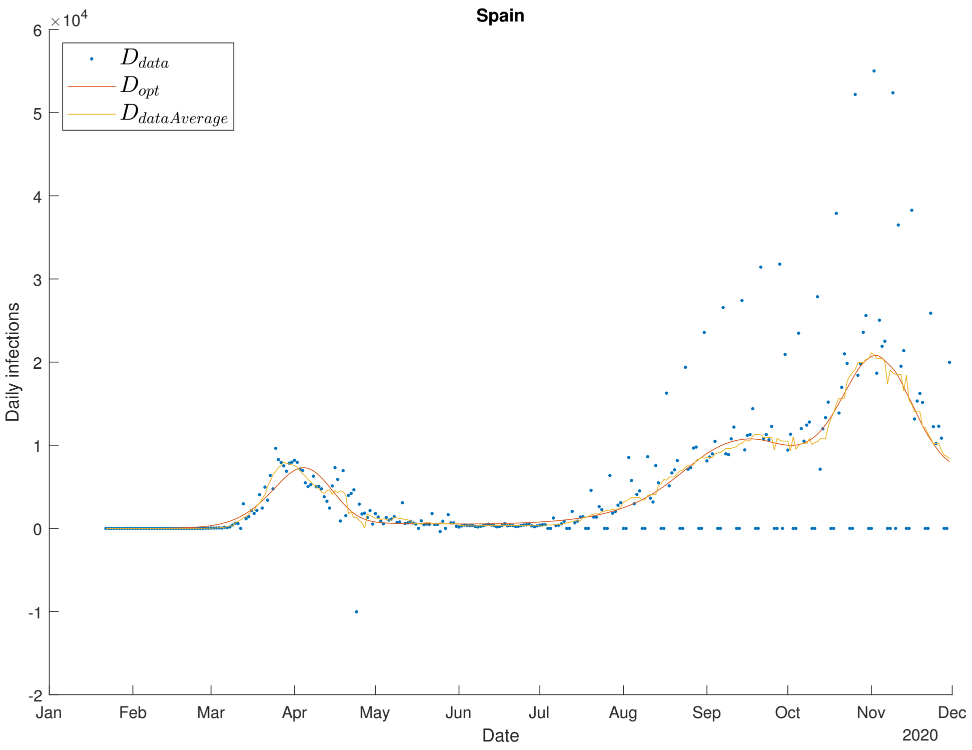

(a) Fit based on $\beta_o pt$ over the training time period. Blue points represent daily infections, the yellow line the seven-day moving average of daily infections, and the red line is the fit obtained by substituting the $\beta_{opt}(t)$ into the SEIR model.

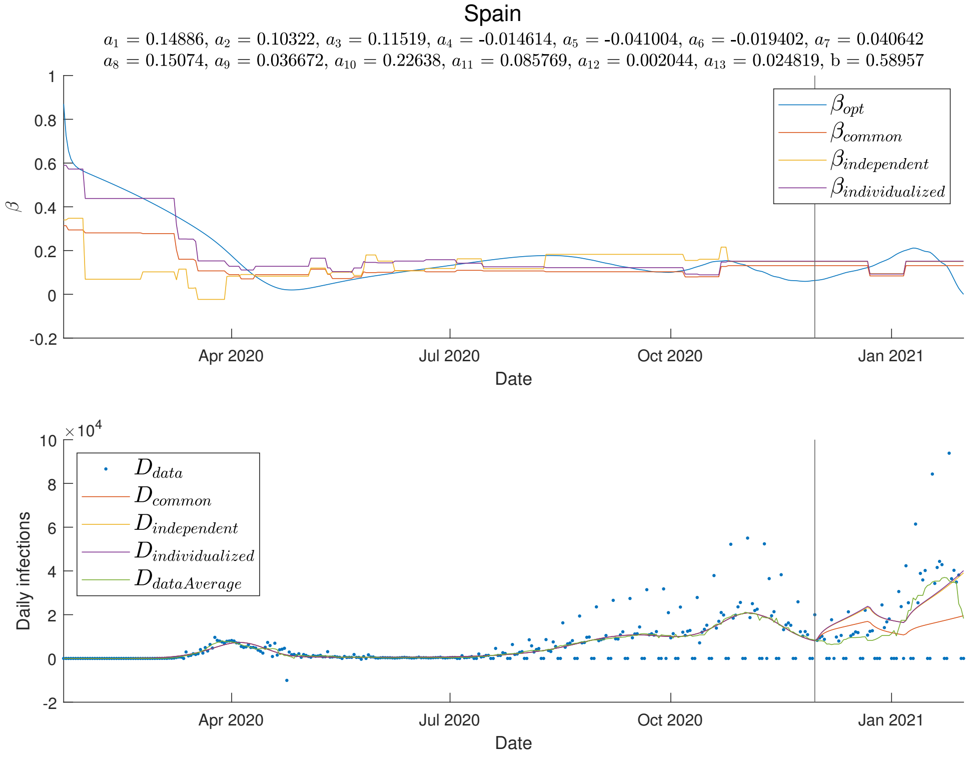

(b) Estimation results. Top panel: fitting a function of restrictions to the signal β_{opt}. Bottom panel: Ability of the model to predict daily infections. The black vertical line indicates the beginning of the validation period. As the number of daily infections is highly variable, a seven day moving average is also presented

Supplementary Figure 79. Prediction results - Spain.

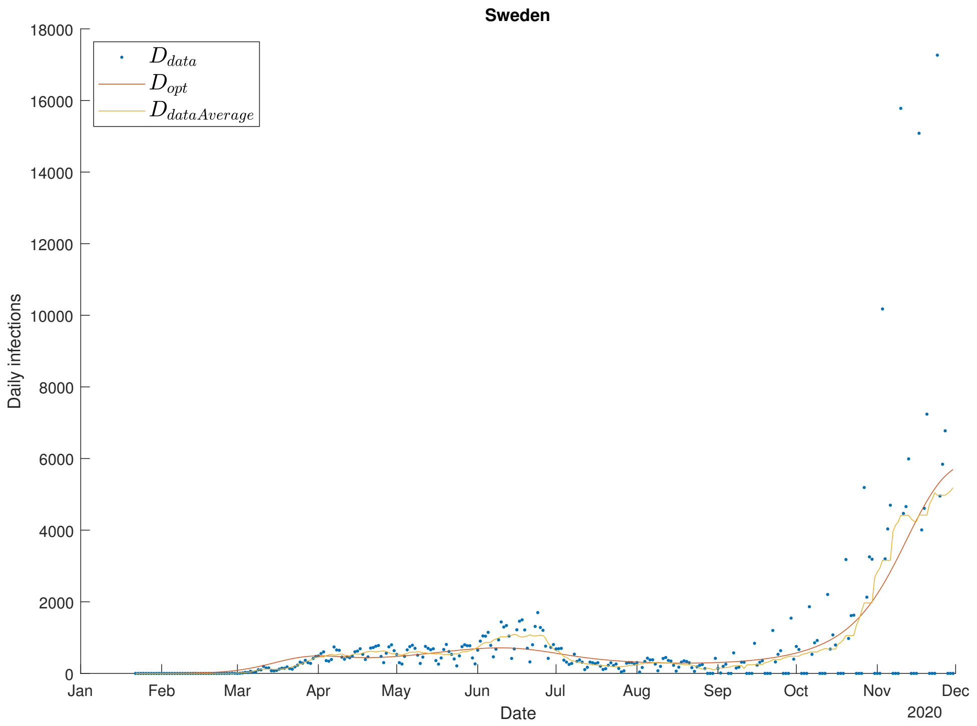

(a) Fit based on β_opt over the training time period. Blue points represent daily infections, the yellow line the seven-day moving average of daily infections, and the red line is the fit obtained by substituting the $\beta_{opt}(t)$ into the SEIR model.

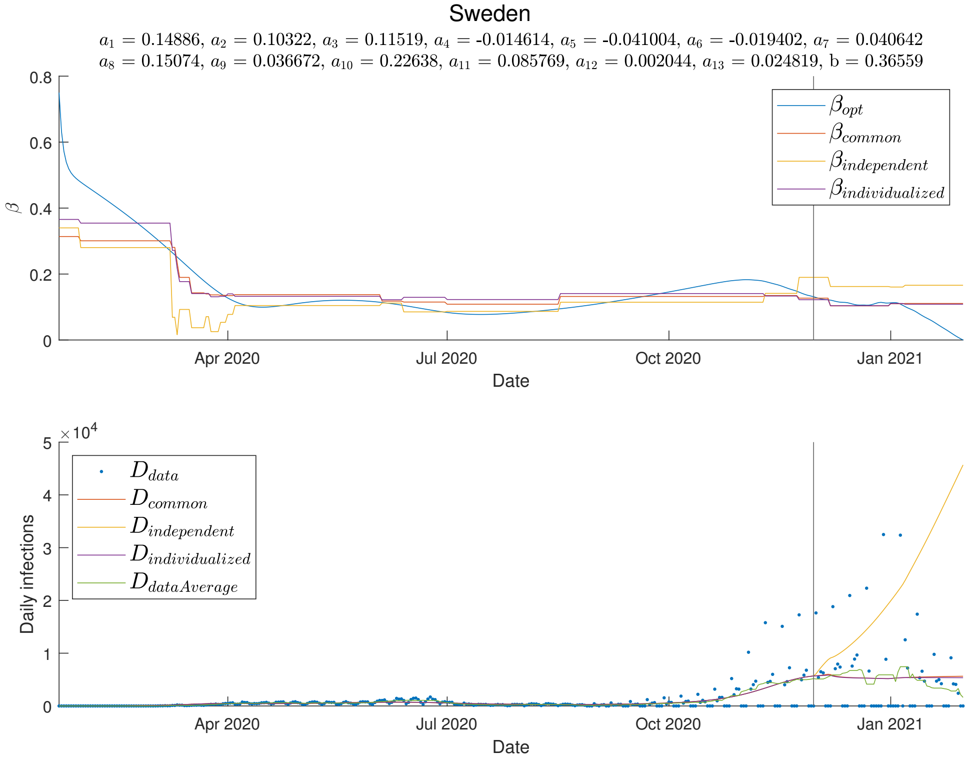

(b) Estimation results. Top panel: fitting a function of restrictions to the signal β_{opt}. Bottom panel: Ability of the model to predict daily infections. The black vertical line indicates the beginning of the validation period. As the number of daily infections is highly variable, a seven day moving average is also presented

Supplementary Figure 80. Prediction results - Sweden.

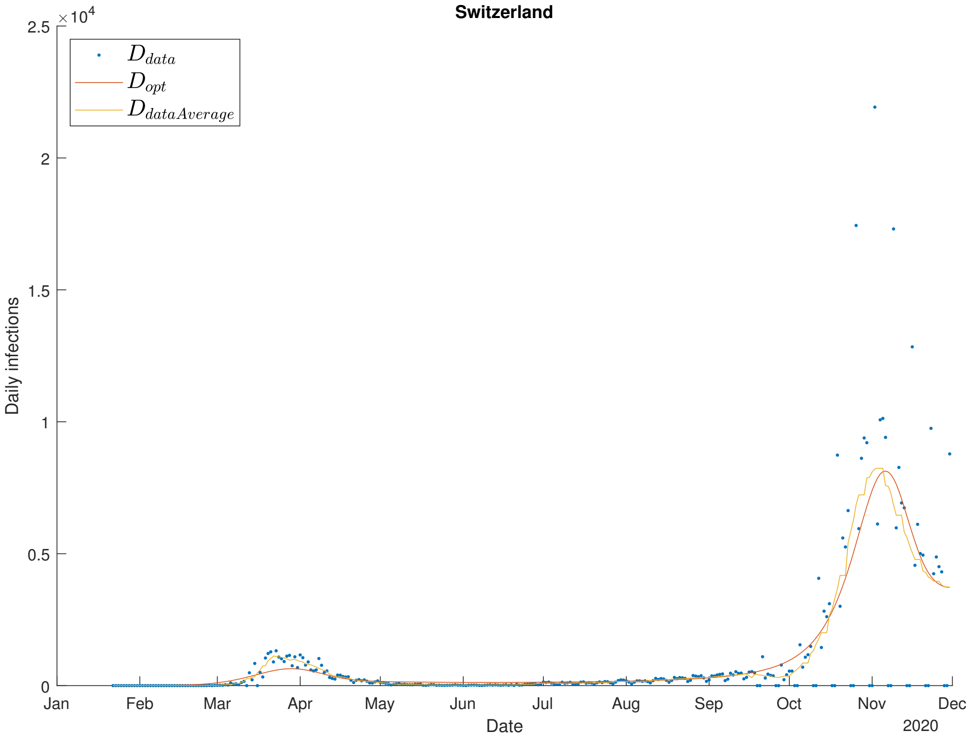

(a) Fit based on $\beta_o pt$ over the training time period. Blue points represent daily infections, the yellow line the seven-day moving average of daily infections, and the red line is the fit obtained by substituting the $\beta_{opt}(t)$ into the SEIR model.

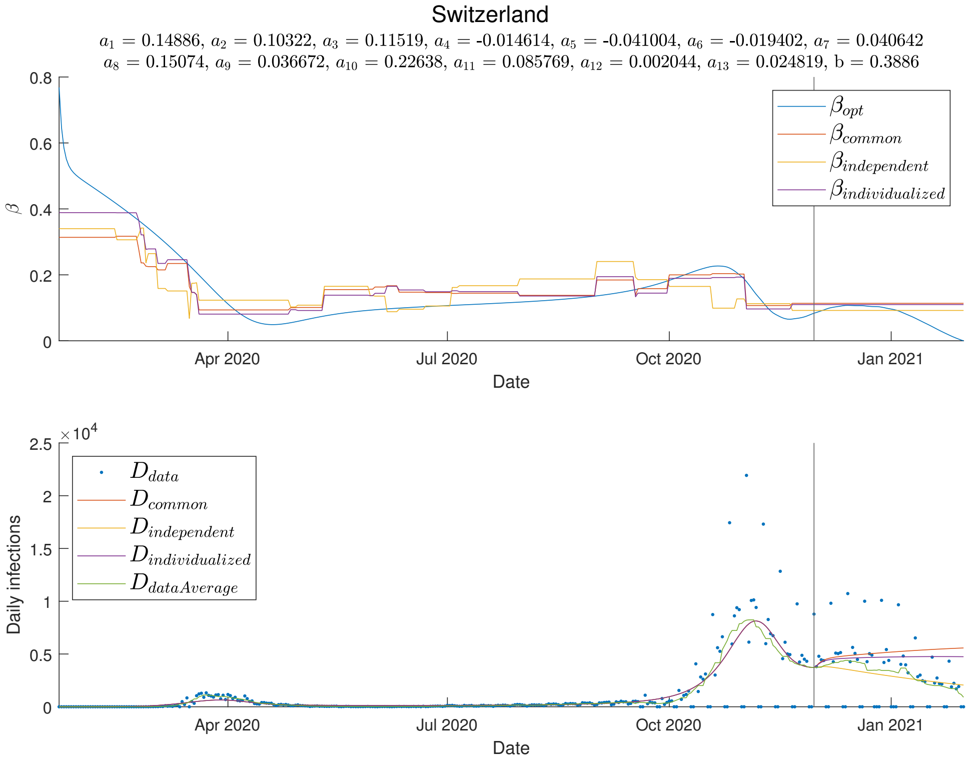

(b) Estimation results. Top panel: fitting a function of restrictions to the signal β_{opt}. Bottom panel: Ability of the model to predict daily infections. The black vertical line indicates the beginning of the validation period. As the number of daily infections is highly variable, a seven day moving average is also presented

Supplementary Figure 81. Prediction results - Switzerland.

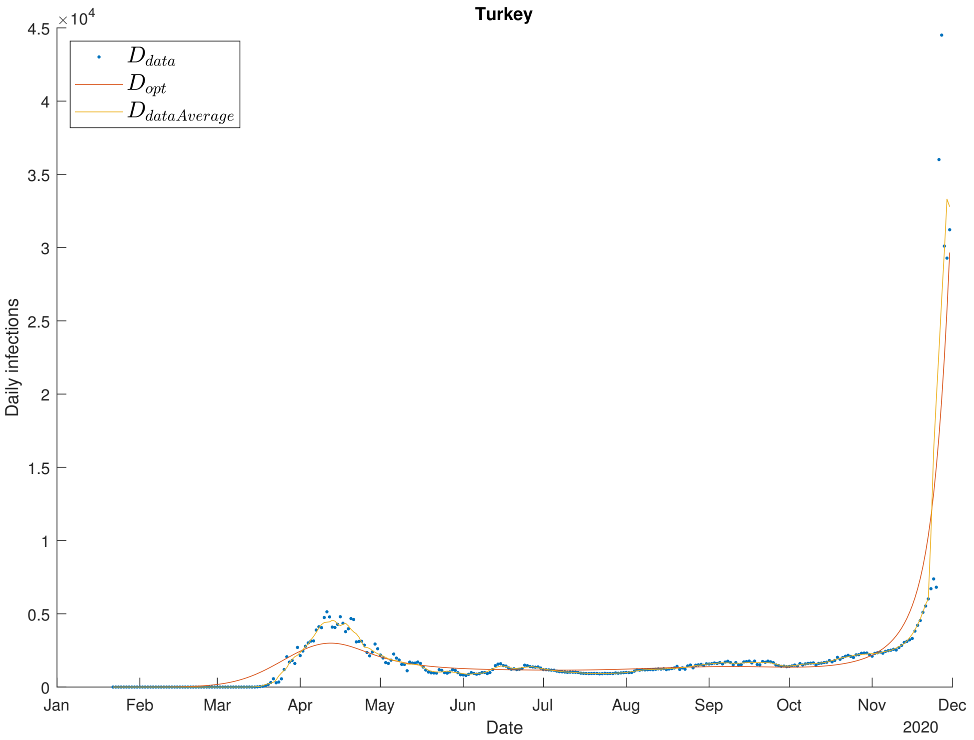

(a) Fit based on $\beta_o pt$ over the training time period. Blue points represent daily infections, the yellow line the seven-day moving average of daily infections, and the red line is the fit obtained by substituting the $\beta_{opt}(t)$ into the SEIR model.

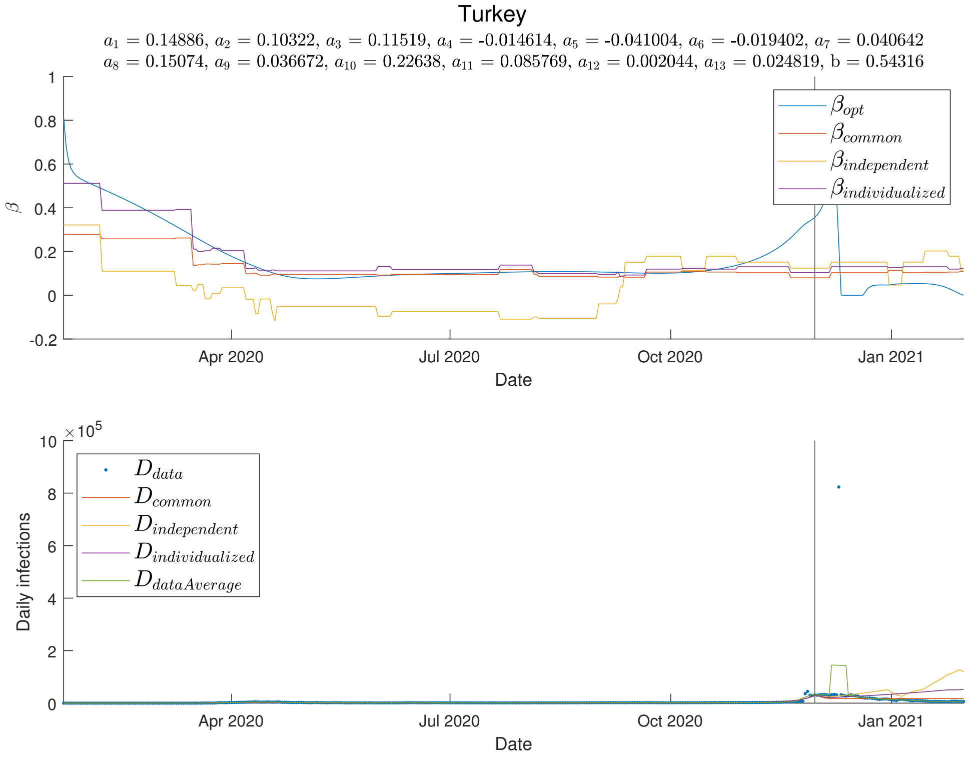

(b) Estimation results. Top panel: fitting a function of restrictions to the signal β_{opt}. Bottom panel: Ability of the model to predict daily infections. The black vertical line indicates the beginning of the validation period. As the number of daily infections is highly variable, a seven day moving average is also presented

Supplementary Figure 82. Prediction results - Turkey.

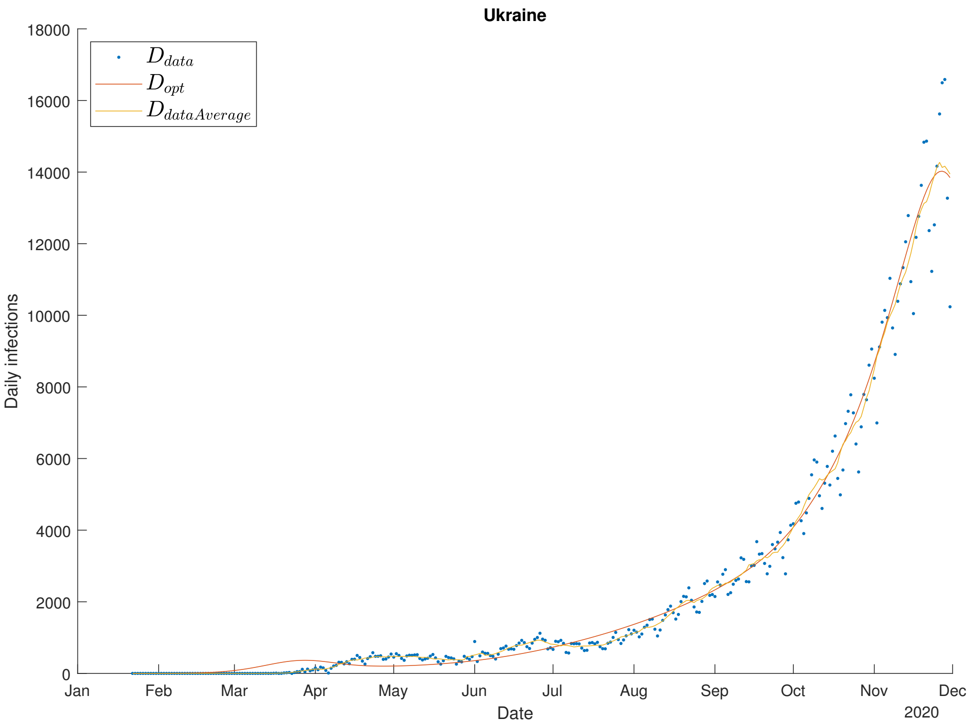

(a) Fit based on $\beta_o pt$ over the training time period. Blue points represent daily infections, the yellow line the seven-day moving average of daily infections, and the red line is the fit obtained by substituting the $\beta_{opt}(t)$ into the SEIR model.

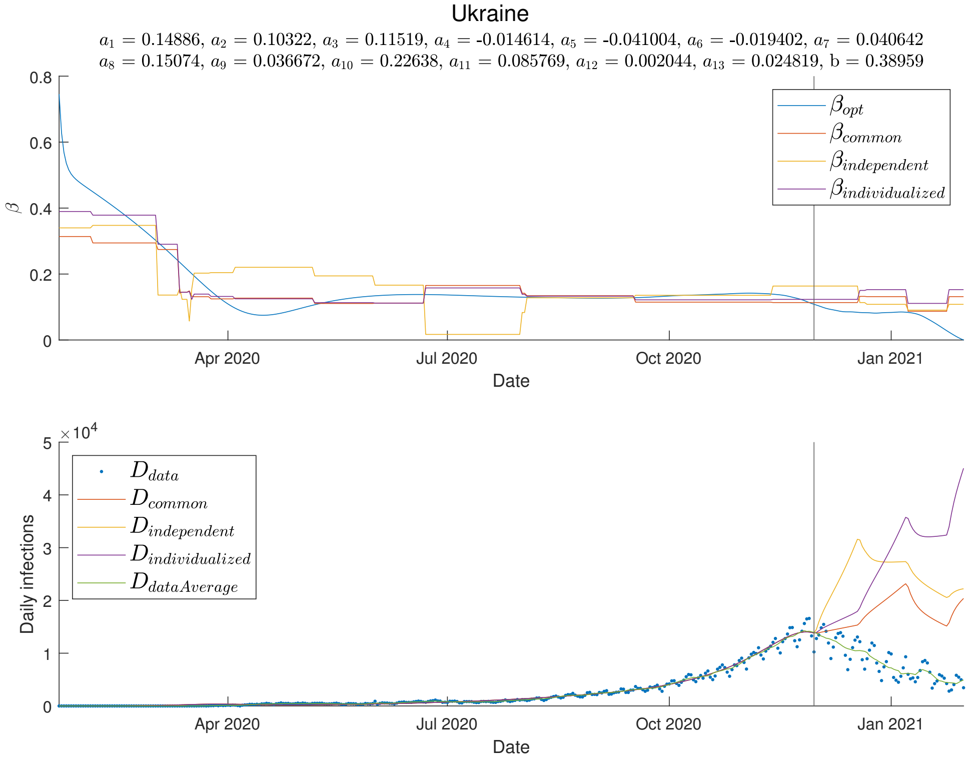

(b) Estimation results. Top panel: fitting a function of restrictions to the signal β_{opt}. Bottom panel: Ability of the model to predict daily infections. The black vertical line indicates the beginning of the validation period. As the number of daily infections is highly variable, a seven day moving average is also presented

Supplementary Figure 83. Prediction results - Ukraine.

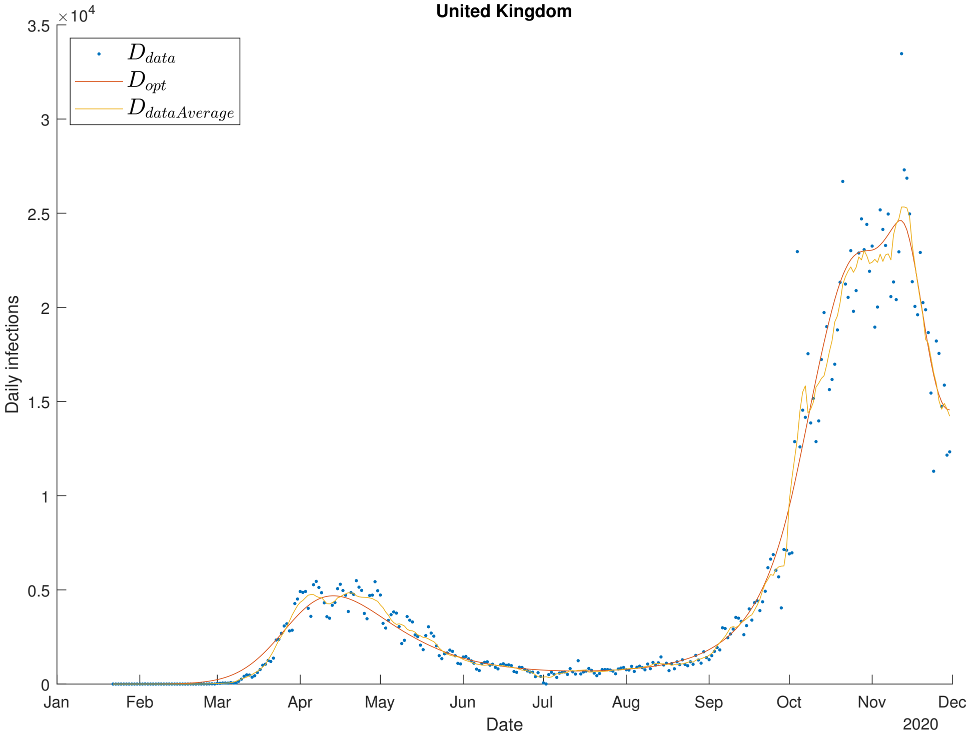

(a) Fit based on $\beta_o pt$ over the training time period. Blue points represent daily infections, the yellow line the seven-day moving average of daily infections, and the red line is the fit obtained by substituting the $\beta_{opt}(t)$ into the SEIR model.

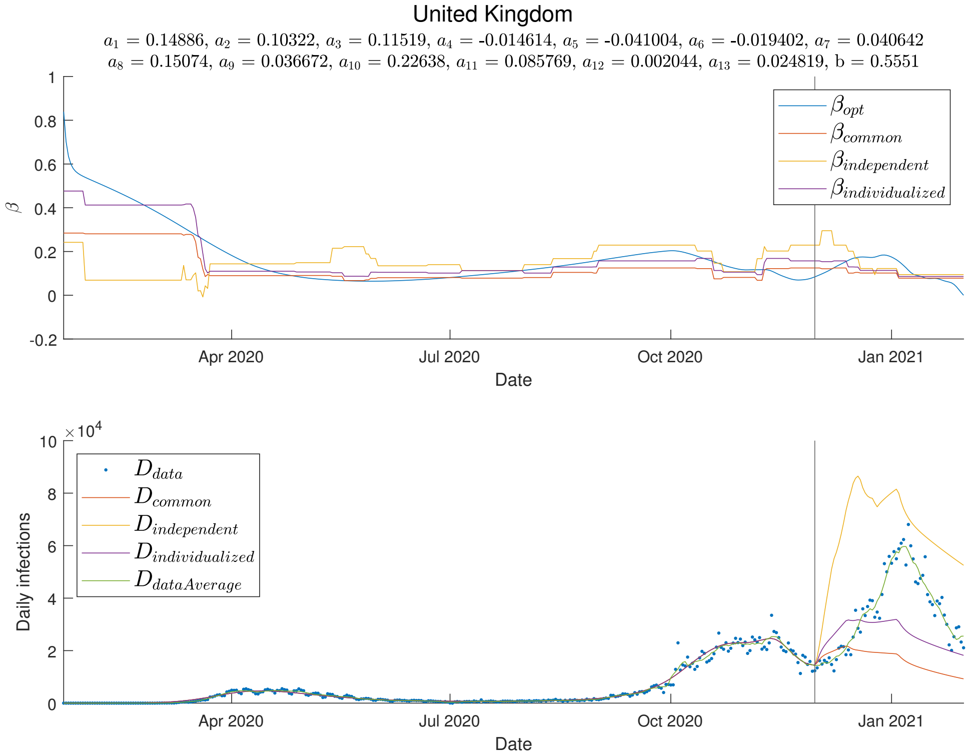

(b) Estimation results. Top panel: fitting a function of restrictions to the signal β_{opt}. Bottom panel: Ability of the model to predict daily infections. The black vertical line indicates the beginning of the validation period. As the number of daily infections is highly variable, a seven day moving average is also presented

Supplementary Figure 84. Prediction results - United Kingdom.
